# Novel Pyridazin-3(2*H*)-one-Based Guanidine Derivatives as Potential DNA Minor Groove Binders with Anticancer Activity

**DOI:** 10.1021/acsmedchemlett.1c00633

**Published:** 2022-02-10

**Authors:** María Carmen Costas-Lago, Noemí Vila, Adeyemi Rahman, Pedro Besada, Isabel Rozas, José Brea, María Isabel Loza, Elisa González-Romero, Carmen Terán

**Affiliations:** † Departamento de Química Orgánica, Universidade de Vigo, 36310 Vigo, España; ‡ Instituto de Investigación Sanitaria Galicia Sur, Hospital Álvaro Cunqueiro, 36213 Vigo, España; § School of Chemistry, Trinity Biomedical Sciences Institute, Trinity College Dublin, 152-160 Pearse Street, Dublin 2, Ireland; ∥ Drug Screening Platform/Biofarma Research Group, CIMUS Research Center. Departamento de Farmacoloxía, Farmacia e Tecnoloxía Farmacéutica. Universidade de Santiago de Compostela, 15782 Santiago de Compostela, España; ⊥ Departamento de Química Analítica y Alimentaria, Universidade de Vigo, 36310 Vigo, España

**Keywords:** pyridazin-3(2H)-one, guanidinium, DNA, antiproliferative activity

## Abstract

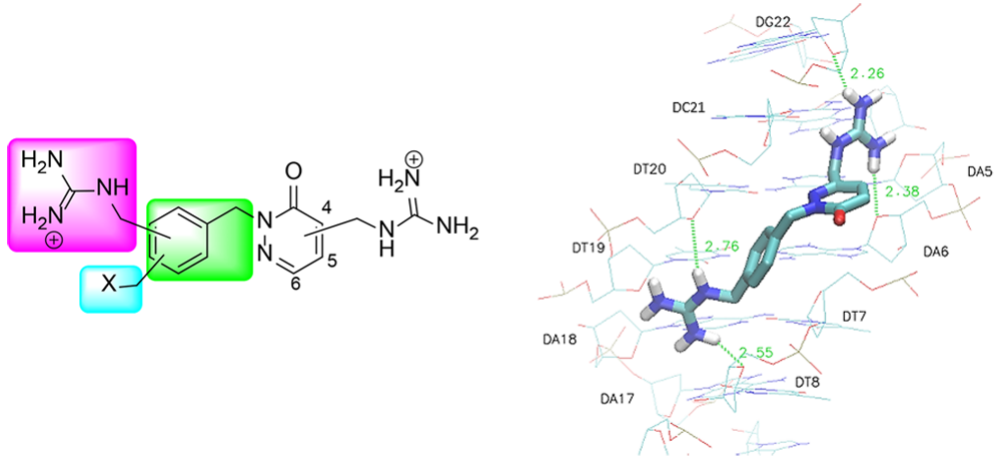

Novel aryl guanidinium analogues containing the pyridazin-3(2*H*)-one core were proposed as minor groove binders (MGBs) with the support of molecular docking studies. The target dicationic or monocationic compounds, which show the guanidium group at different positions of the pyridazinone moiety, were synthesized using the corresponding silyl-protected pyridazinones as key intermediates. Pyridazinone scaffolds were converted into the adequate bromoalkyl derivatives, which by reaction with *N*,*N*’-di-Boc-protected guanidine followed by acid hydrolysis provided the hydrochloride salts **1**–**14** in good yields. The ability of new pyridazin-3(2*H*)-one-based guanidines as DNA binders was studied by means of DNA UV-thermal denaturation experiments. Their antiproliferative activity was also explored in three cancer cell lines (NCI-H460, A2780, and MCF-7). Compounds **1**–**4** with a bis-guanidinium structure display a weak DNA binding affinity and exhibit a reasonable cellular viability inhibition percentage in the three cancer cell lines studied.

Deoxyribonucleic acid (DNA) is a key molecular target for chemotherapy since inhibition of its normal functions, such as replication or gene expression and, hence, cell growth and division, has potential therapeutic application in a wide set of pathologies from infectious diseases to cancer.^1,2^ There are several mechanisms by which drugs can target the DNA double helix, with intercalation, alkylation, strand cleavage, and binding to the minor groove being the most common.^1^ Minor groove binders (MGBs) usually show a planar and concave structure to fit the groove curvature.^3,4^ They are aromatic compounds containing hydrophobic regions, which remove the hydration spine along the groove. Additionally, they display cationic groups under physiological pH, suitable for ionic interactions with the negative potential of the minor groove and to form hydrogen bonds (HBs) with specific DNA base sequences at the groove floor.^5^ The structural changes caused in the DNA helix by MGBs can disrupt essential protein or transcription factor–DNA interactions.^6,7^

The discovery of the anti-infective and cytotoxic activity of naturally occurring netropsin^8^ and distamycin,^9^ inspired the development of synthetic MGBs therapeutically applicable in cancer or infectious diseases.^1,2,10−12^ Although the antimicrobial activity of aromatic diamidines such as pentamidine (Figure 1) was described in the 1940s,^13^ knowledge of amidinium oligoamides targeting the DNA minor groove has significantly enhanced the development of small aromatic and heteroaromatic amidine compounds as MGBs.^12,14,15^ Readily ionizable amidine-like functionalities, such as guanidine, 2-aminoimidazoline,^16^ or isourea,^17^ are also present in these types of analogues. Examples of classical amidine MGBs include the previously cited pentamidine, beneril, furamidine, or its prodrug pafuramide (Figure 1), with all of them therapeutically relevant against a range of microbial and parasitic diseases.^2,10,11^ In addition, furamidine and several furamidine analogues, such as the benzimidazole derivative BD293 (Figure 1), have also displayed good antiproliferative effects on different tumor cell lines.^18,19^

**Figure 1:**
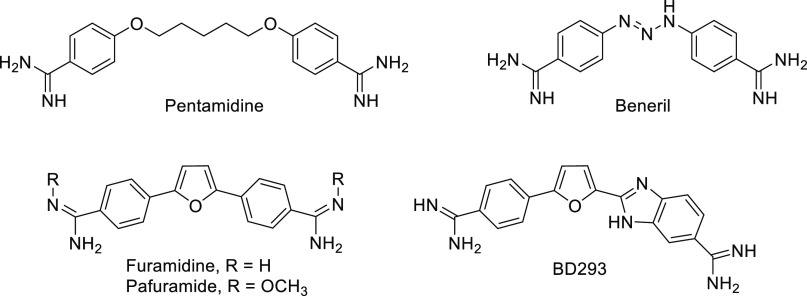
Examples of classical amidine MGBs with antibacterial, antiparasitic, or anticancer activities.

Over the past few years, Rozas’ group has been performing extensive work in the field of MGBs.^20−23^ Several families of symmetric and asymmetric diaryl guanidine-like analogues with potential antineoplastic or antiparasitic activity were obtained. Some of these analogues in which the diaryl fragments are connected by different linkers (Figure 2) exhibited strong affinity by DNA and good sequence selectivity.

**Figure 2:**
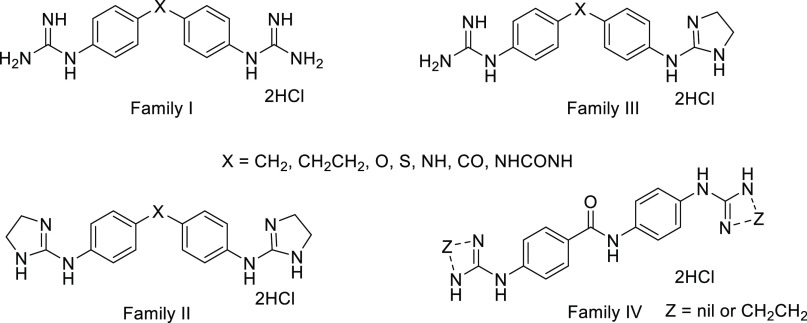
General structure of some guanidine and 2-aminoimidazole dicationic prototypes previously reported by Rozas’ group.

Hence, looking for new guanidine derivatives as MGBs, we have explored the potential of the pyridazin-3(*2H*)-one core, a significant scaffold in Medicinal Chemistry,^24^ that could possibly establish extra interactions with DNA (i.e., with the nucleobases or the phosphate-sugar strand). Thus, we have designed a series of bis-guanidinium analogues related to Family I (X = CH_2_ in Figure 2), in which one of the phenyl groups was replaced by a pyridazin-3(2*H*)-one moiety with the attached guanidinium placed at different positions of the diazine ring (compounds **1**–**4**, Figure 3). Our hypothesis is that the benzene/pyridazin-3(*2H*)-one replacement could enhance the ability of these compounds to establish HBs in the DNA minor groove, an important factor for the drug–DNA complex stabilization. Likewise, the location of the guanidinium in different positions of the pyridazin-3(2*H*)-one system will allow the investigation of how the different distance and orientation of these cations would affect their DNA binding affinity.

**Figure 3:**
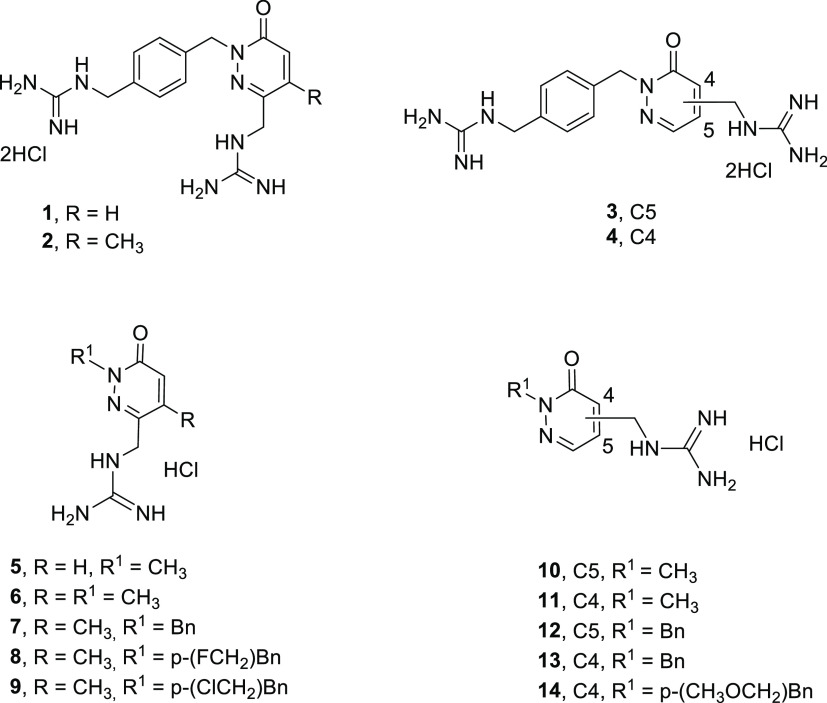
Compounds proposed in this study as potential MGBs.

In addition, to extend this initial set of pyridazin-3(*2H*)-one-based bis-guanidinium derivatives, we have also studied a series of monocationic analogues devoid or not of the phenyl core (compounds **5**–**14**, Figure 3). The novel monocationic analogues would allow us to analyze the significance of different molecule parts in pyridazinone-based guanidinium compounds for the interaction with DNA.

First, we carried out docking studies of the compounds proposed in a model of the DNA minor groove (a dodecanucleotide d(CGCGAATTCGCG)_2_ complexed with the drug pentamidine, PDB: 1D64, resolution of 2.1 Å^25^) to assess their potential as MGBs. The structures of all proposed ligands (**1**–**14**) were optimized at DFT level (using the M06-2X functional and the 6-31+G(d,p) basis set) with the SMD solvation model for water as implemented in Gaussian16^26^ (see Supporting Information (SI), Figure S1). Then, docking studies were performed with the Autodock Vina program^27^ and the optimized ligand structures were docked to the oligonucleotide model in a rigid-flexible approach.

Figure 4 shows the best docking pose of compound **1** in the mentioned model of the DNA minor groove indicating the HBs formed. Figures S2–S14 (SI) display the best docking poses for the rest of target compounds (**2**–**14**), and Table S1 (SI) illustrates the distances, angles, and atoms involved in the weak/medium interactions formed in each case.

**Figure 4:**
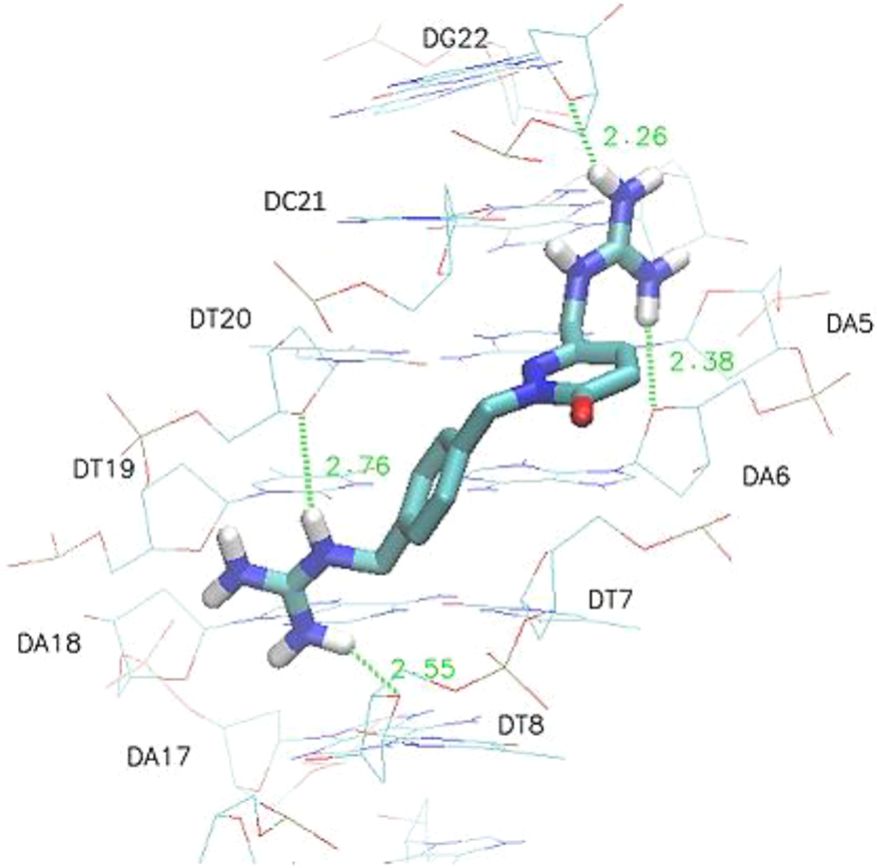
Best pose obtained in the docking of compound **1** to the DNA minor groove model (dodecanucleotide d(CGCGAATTCGCG)_2_, PDB: 1D64), with a G-score of −8.9 kcal/mol, using a flexible-rigid approach and the Autodock Vina program. Green lines and numbers indicate HBs, and HB distances are in Å.

Compounds **1**–**4** showed the stronger G-scores when binding to the minor groove model (> –7.7 kcal/mol), in agreement with the formation of weak HBs (average HB distances 2.47 Å) between one or both guanidinium cations and O atoms in the oligonucleotide strands (mostly of the sugar moieties). Compounds **5**–**6** and **10**–**11**, which are monoaryl guanidinium systems, have the poorest G-scores (≤-6.8 kcal/mol) with a small number of HB interactions through their guanidinium functionality and a thymidine base. In general, diaryl monoguanidinium systems (**7**–**9** and **12**–**14**) showed slightly higher G-scores (between −6.3 to −7.4 kcal/mol) than those of the monoaryl derivatives but lower scores than those of the bis-guanidinium compounds **1**–**4**; this group of compounds also showed a small number of weak HBs formed between the guanidinium group and different bases (guanine, thymine, or adenine).

Considering that the outcome of the docking studies was generally positive, all the compounds proposed were synthesized, in moderate to good yields and purities ≥ 94%, using the adequate silyl-protected pyridazinones as key scaffolds. Thus, the pyridazin-3(2*H*)-one core was obtained from simple furan derivatives (**15**–**17**), whose conversion into the appropriate silyl-protected hydroxyalkylfuran (**18**, **19** and **20**), followed by oxidation with singlet oxygen in specific conditions provides γ-methoxy (**21**) or γ-hydroxy (**22**–**24**) butenolides. These butenolides react with hydrazine or monosubstituted hydrazines, resulting in the desired diazinone scaffolds **25**–**35** (Scheme S1, SI).^28−30^

The simultaneous inclusion of the two guanidine fragments, by using the corresponding bis-bromoalkyl derivatives and *N*,*N*’-di-Boc-protected guanidine was attempted to synthesize the bis-guanidinium derivatives **1**–**4**, (Scheme S2, SI and Scheme 1). Direct incorporation of a 4-bromomethylbenzyl group via alkylation of silyl-protected pyridazinones **25**, **26**, **30**, and **31** with α,α’-dibromo-*p*-xylene, followed by alcohol deprotection and bromination, would provide the desired bromine analogues. However, the significant reactivity differences observed in hydroxyl deprotection when the *p*-(bromomethyl)benzyl fragment was located at N2 of the pyridazinone core led us to utilize methyl 4-bromomethyl benzoate as the alkylating agent (Scheme S2, SI). Treatment of pyridazinones **25**, **26**, **30**, and **31** with methyl 4-bromomethyl benzoate and NaH in DMF at room temperature provided esters **36**–**39**, respectively, in very good yields (76–96%). Next, treatment with DIBAL-H in THF at –78°C to yield alcohols **40**–**43**, cleavage of the silyl ether with TBAF in THF, and bromination of diol analogues **44**–**47** by refluxing with carbon tetrabromide and triphenylphosphine in methylene chloride successfully provided the dibromide analogues **48**–**51** (Scheme S2, SI). Now it was possible to prepare the desired bis-guanidinium salts **1**–**4** in good yields by the reaction of derivatives **48**–**51** with 1,3-bis(*tert*-butoxycarbonyl)guanidine in the presence of K_2_CO_3_ to yield Boc-protected guanidines **52**–**55**, that were then deprotected using 4 M HCl/1,4-dioxane (Scheme 1).

**Scheme 1:**
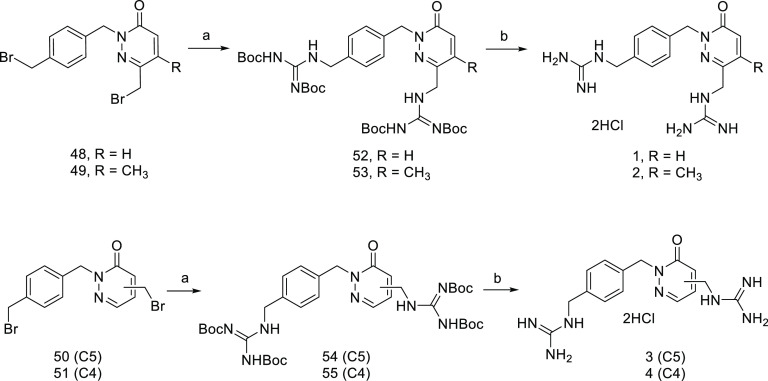
Preparation of Bis-guanidinium Derivatives **1**–**4** Reagents and conditions: (a) 1,3-bis(*tert*-butoxycarbonyl)guanidine, K_2_CO_3_, DMF, 50 °C, 2 h, 60% (**52** and **53**), 61% (**54**), 70% (**55**); (b) HCl 4 M in 1,4-dioxane, dioxane, 55 °C, 5 h, 80% (**1**), 83% (**2**), 85% (**3**), 94% (**4**).

The synthesis of monoguanidinium analogues **5**–**14** (Figure 3) was performed in a similar way from the corresponding silyl-protected pyridazinones substituted at N2. Alcohol deprotection in pyridazinones **27**–**29** and **32**–**35** was successfully accomplished using standard conditions, thus providing the corresponding hydroxymethyl derivatives **56**–**62**. These were then converted into the desired bromomethyl pyridazinones **63**–**69** in moderate to good yield by treatment with carbon tetrabromide and triphenylphosphine (Scheme S3, SI). The silyl protected pyridazinones derivatized with a 4-bromomethylbenzyl group at N2 (**70**–**71**) were obtained from analogues **25** and **31** by using α,α’-dibromo-*p*-xylene as the alkylating agent (Scheme S4, SI). Treatment of **70** with TBAF in THF allowed the hydroxyl group deprotection, also causing a bromine to fluorine exchange at the benzylic position, even when the reaction was performed at 0 °C, to give compound **72** in moderate yield (68%). However, in the same deprotection conditions, the C4-substituted analogue **71** gave a complex mixture of products. Therefore, **71** was alternatively deprotected with a catalytic amount of bromotrimethylsilane (TMBS) in methanol at reflux, causing in this case a bromine/methoxy replacement and providing the alcohol **73** in 75% yield. Subsequent bromination of compounds **72** and **73** with carbon tetrabromide and triphenylphosphine afforded the corresponding bromo analogues **74** and **75** in moderate to excellent yields (Scheme S4, SI). Finally, monobromo derivatives **63**–**69**, **74**, and **75** were reacted with guanidine 1,3-bis-Boc protected followed by acid hydrolysis, providing the hydrochloride salts **5**–**14** in moderate to very good yields (Scheme 2).

**Scheme 2:**
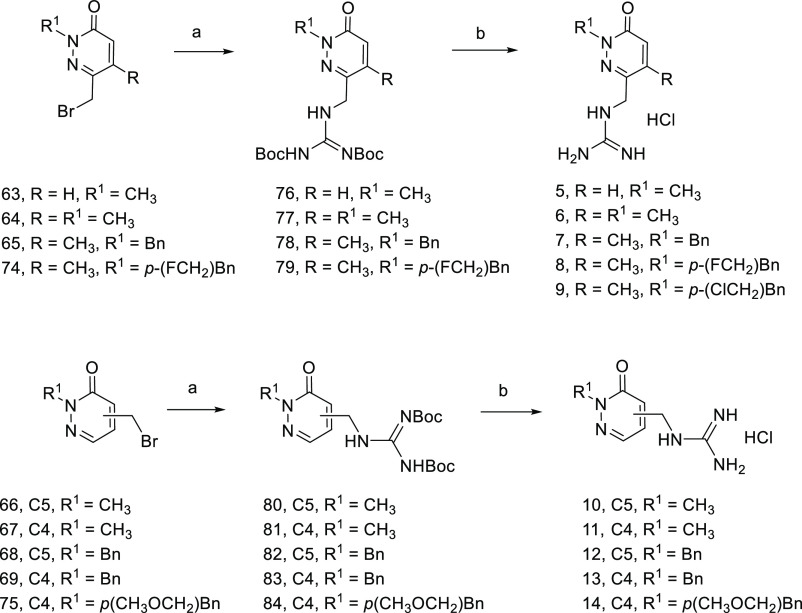
Preparation of Monoguanidinium Derivatives **5**–**14** Reagents and conditions: (a) 1,3-bis(*tert*-butoxycarbonyl)guanidine, K_2_CO_3_, DMF, 50 °C, 2 h, 57% (**76**), 55% (**77)**, 59% (**78**), 60% (**79**), 94% (**80**), 84% (**81**), 90% (**82**), 83% (**83**),62% (**84**); (b) HCl 4 M in 1,4-dioxane, dioxane, 55 °C, 5 h, 77% (**5**), 92% (**6**), 86% (**7**), 33% (**8**) and 46% (**9**), 92% (**10**), 89% (**11**), 92% (**12**), 90% (**13**), 99% (**14**).

In the case of bromofluoro derivative **74**, with two possible reactive positions, it is worth noting that guanidine fragment inclusion occurs exclusively on the benzylic carbon adjacent to bromine, even when the reaction was accomplished with 2 equiv of 1,3-bis(*tert*-butoxycarbonyl) guanidine, giving rise to the di-Boc-protected monoguanidine analogue **79** in moderate yield. However, the acid removal of the Boc groups in **79** induced a partial replacement of fluorine by chlorine, providing the mixture of halo substituted guanidinum salts **8** and **9**, in which the chlorine analogue **9** predominates. Compounds **8** and **9** were purified, successfully separated by reverse phase column chromatography, and unequivocally characterized by NMR and mass spectroscopic data.

Once all proposed bis- and monoguanidinium salts had been prepared, their ability as DNA binders was explored through a fast and reliable screening of UV-thermal denaturation, which was performed using unspecific salmon testes DNA (68% adenine-thymine base pair content, st-DNA).^20,21^ Stated briefly, the DNA duplex denaturation assay was performed by heating the sample in a temperature range of 30–90 °C. The thermal melting temperature (*T*_m_) was calculated from the increase in UV absorbance caused by the double helix splitting in two individual strands. Thus, the interaction of target compounds with st-DNA was analyzed by comparing the *T*_m_ of st-DNA alone and in the presence of every compound. The *T*_m_ increase (Δ*T*_m_) is directly related to the ligand–DNA binding affinity and consequently with the stability of the complex formed.

A weak increase in DNA *T*_m_, was observed for bis-guanidinium derivatives **1**–**4**, hardly affected by the change in the location of the guanidinium fragment in the pyridazinone core, with Δ*T*_m_ values ranging from 1.1 to 1.4 °C (Table 1 and Figure S15, SI). In addition, and in agreement with the G-score values obtained in the docking studies, no variations in *T*_m_ of DNA were observed for the monoguanidium analogues **5**–**14**, suggesting a lack of DNA binding. This may be explained because even though compounds **8**, **9**, and **14**, in which the second guanidium moiety was replaced by neutral HB acceptor groups, show a similar molecular shape to **1**–**4**, they lack the second cationic system that seems essential to DNA binding.

**Table 1:** DNA Binding Affinity (Δ*T*_m_) for Compounds **1**–**4**

compd	Δ*T*_m_, st-DNA (°C)^a^
**1**	1.4
**2**	1.2
**3**	1.2
**4**	1.1

a The increment in DNA thermal melting (Δ*T*_m_, °C) was measured in unspecific salmon sperm DNA. The melting temperature of salmon sperm DNA in phosphate buffer (10 mM) was 67.4 °C.

Overall, a decrease in DNA binding affinity was detected for these novel bis-guanidinium-like derivatives with respect to diphenyl dicationic analogues previously described (Family I, Figure 2), which could be related to the higher hydrophilicity of the diazinone core.

Despite these disappointing results in terms of DNA binding, we proceeded to assess the effect of a representative sample of synthesized compounds (i.e., **2**, **3**, **5**–**14**) on the cell viability of a number of cancer cell lines such as NCI-H460 (human lung carcinoma), A2780 (human ovarian carcinoma), and MCF-7 (human breast adenocarcinoma) using cisplatin as the reference drug, and the obtained results are presented in Table 2.

**Table 2:** Effect on the Cell Viability of Cancer Cells NCI-H460 (Human Lung Carcinoma), A2780 (Human Ovarian Carcinoma), and MCF-7 (Human Breast Adenocarcinoma), Expressed as Inhibition Percentage of Cell Viability at 100 μM, for a Selection of Pyridazin-3(2*H*)-one-Based Guanidine Derivatives and Reference Drug (Cisplatin)

compd	NCI-H460 (%)^a^	A2780 (%)^a^	MCF-7 (%)^a^
**2**	34 ± 3	59 ± 2	22 ± 2
**3**	35 ± 3	33 ± 3	23 ± 2
**5**	4 ± 1	11 ± 1	20 ± 2
**6**	2 ± 2	20 ± 3	12 ± 2
**7**	2 ± 1	23 ± 1	18 ± 2
**8**	5 ± 2	34 ± 2	13 ± 2
**9**	7 ± 1	54 ± 2	25 ± 1
**10**	13 ± 4	24 ± 3	2 ± 2
**11**	1 ± 1	18 ± 4	1 ± 2
**12**	1 ± 1	40 ± 2	13 ± 2
**13**	15 ± 3	41 ± 2	46 ± 4
**14**	1 ± 1	38 ± 1	22 ± 2
**Cisplatin**	62 ± 4	97 ± 1	84 ± 2

a Values are means of three experiments.

As it can be seen, the studied compounds, with exception of compounds **2** and **9**, show inhibition percentages of cell proliferation lesser than 50% at 100 μM in the three cancer cell lines.

However, depending on the cancer cell line, different trends were observed. In general, the best percentage inhibition was observed for the ovarian cancer A2780 cell line (11–59%) and the worst percentage inhibition values were obtained for the NCI-H460 cancer cell line (1–35%). In the case of the MCF-7 breast cancer cell line, similarly poor percentage inhibition is observed for most of the compounds tested (1–25%) with the exception of diaryl monoguanidinium derivative **13** with an inhibition percentage of 46%.

Regarding the A2780 ovarian cancer cell line, as was previously mentioned, the best results were obtained for compounds **2** (bis-guanidinium) and **9** (diaryl monoguanidinium), with values of 59% (IC_50_ = 21 ± 1 μM) and 54% (IC_50_ > 100 μM), respectively, followed by compounds **3** (bis-guanidinium derivative), **8** and **12**–**14** (diaryl monoguanidinium analogues) with percentage inhibition values between 33 and 41%. The rest of the monoguanidinium analogues (**5**–**7**, **10**, and **11**), which are all monoaryl derivatives, showed poor inhibitory values (10–20%). Interestingly, the presence of the diaryl core seems to correlate with the inhibition observed since those compounds lacking one of the aromatic systems showed the worst percentage inhibition in the A2780 cell line.

In addition, compounds **2**, **3** (bis-guanidinium analogues) and **9**, **12**–**14** (diaryl monoguanidinium derivatives) also provided the best inhibition percentage in the MCF-7 cell line.

Finally, in the case of the NCI-H460 cell line, the worst inhibition values (<10%) were observed for most of the monoguanidinium salts (i.e., **5**–**9**, **11**, **12**, and **14**) compared to the bis-guanidinium derivatives **2** and **3** that showed 34–35% inhibition. This is in agreement with the docking and DNA binding results.

In conclusion, new aryl guanidinium compounds of dicationic or monocationic structure and with the guanidinium group placed at different positions of the pyridazinone core were synthesized and studied as potential MGBs. The ability of target compounds to bind to DNA was assessed by UV-thermal melting experiments using unspecific st-DNA, and their antiproliferative activity was screened against three cancer cell lines (NCI-H460, A2780, and MCF-7). Among all proposed compounds, only bis-guanidinium analogues exhibited a weak DNA-binding affinity, revealing that the two terminal guanidinium moieties are essential for binding to DNA. These bis-guanidinium analogues exhibited a moderate antiproliferative effect in the three cancer cell lines, and it is worth mentioning compound **2**, with an IC_50_ value of 21 ± 1 μM in the A2780 cell line. From the biophysical experiments, we cannot conclude that this activity is a consequence of DNA binding. In addition, the presence of the diaryl core seems to correlate with the inhibition observed since most of the diaryl monoguanidinium analogues also provided a moderate inhibition percentage, in particular in A2780 and MCF-7 cell lines.

## Supporting Information Available

Molecular modeling experimental data, additional figures illustrating optimized structures and docking poses, tabulated docking study data, additional schemes and experimental procedures, biophysical and biological assays, NMR spectra of target compounds (PDF)

## Abbreviations

A2780: human ovarian carcinoma
Boc: *tert*-butyloxycarbonyl
DFT: density functional theory
DIBAL-H: diisobutylaluminum hydride
DMF: *N*,*N*-dimethylformamide
HBs: hydrogen bonds
MCF-7: human breast adenocarcinoma
MGBs: minor groove binders
NCI-H460: human lung carcinoma
PDB: protein data bank
SMD: solvation model based on density
st-DNA: salmon testes DNA
TBAF: tetra-*n*-butylammonium fluoride
Tm: thermal melting temperature
TMBS: bromotrimethylsilane
