# Supplementary material for: Novel Pyridazin-3(2H)-one-Based Guanidine Derivatives as Potential DNA Minor Groove Binders with Anticancer Activity
Source: ACS Med Chem Lett. 2022 Feb 10;13(3):463–9. doi: 10.1021/acsmedchemlett.1c00633 (PMC8919506; doi:10.1021/acsmedchemlett.1c00633)
Supplement: Supplementary file 1 — ml1c00633_si_001.pdf [file ml1c00633_si_001.pdf]

SUPPORTING INFORMATION

Novel pyridazin-3(2H)-one-based guanidine derivatives as potential DNA minor groove binders with anticancer activity

María Carmen Costas-Lago,[a,b] Noemí Vila,[a,b] Adeyemi Rahman,[c] Pedro Besada,[a,b] Isabel Rozas,[c] José Brea,[d] María Isabel Loza,[d] Elisa González-Romero,[e] Carmen Terán*[a,b]

[a]Universidade de Vigo, Departamento de Química Orgánica, 36310 Vigo, España
[b]Instituto de Investigación Sanitaria Galicia Sur, Hospital Álvaro Cunqueiro, 36213 Vigo, España
[c]School of Chemistry, Trinity Biomedical Sciences Institute, Trinity College Dublin, 152-160 Pearse street, Dublin 2, Ireland
[d]Drug Screening Platform/Biofarma Research Group, CIMUS Research Center. Departamento de Farmacoloxía, Farmacia e Tecnoloxía Farmacéutica. Universidade de Santiago de Compostela, 15782 Santiago de Compostela, España
[e]Universidade de Vigo, Departamento de Química Analítica y Alimentaria, 36310 Vigo, España

Table of Contents

Molecular modelling studies…………………………………………………………...S1
Optimised structures of compounds **1-14**………………………………………………...S2
Molecular docking figures…………………………………………………………...S4
Tabulated data of docking study……………………………………………………...S17
Schemes of the preparation of scaffolds and precursors needed……………………...S18
Synthesis of compounds **1-14**…………………………………………………………S21
Biophysical studies……………………………………………………………………S41
Biological studies……………………………………………………………………..S42
NMR spectra of pyridazin-3(2H)-one-based guanidine derivatives **1-14**…………….....S43
References…………………………………………………………………………….....S50

Molecular modelling studies

Ligand optimization

All ligands were fully optimized at DFT level (M06-2X functional) with the 6-311+G* basis set using the Gaussian16 program [1]. Frequency calculations were performed at the same computational level to confirm that the resulting optimized structures were energetic minima. The effect of water solvation was accounted using the

SCRF-SMD approach implemented in the Gaussian16 package including dispersing, repulsing and cavitation energy terms of the solvent in the optimization. Optimised structures of all the ligands studied are shown in Figure S1.

Docking experiments

The program Autodock Vina 4.2 was used to carry out docking studies [2]. The ligands were flexibly docked into the rigid DNA minor groove model (crystal structure of a pentamidine-oligonucleotide complex, PDB: 1D64 [3]). Scores (G-scores) were measured in kcal/mol and are only indicative of the quality of the interaction with the target; they do not provide a quantitative measure of binding. Poses obtained from the docking were visualised with VMD [4].

Best poses obtained for the docking of compounds **2-14** into the minor groove model (dodecanucleotide d(CGCGAATTCGCG)$_2$ complexed with the drug pentamidine, PDB: 1D64) are shown in Figures S2 to S14.

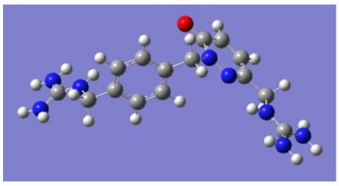

Compound **1**
E (Hartree) = -1097.479658

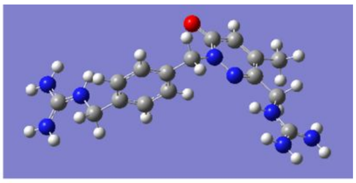

Compound **2**
E (Hartree) = -1136.782542

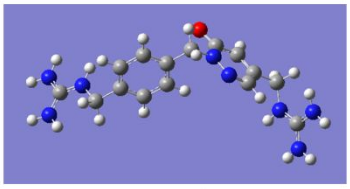

Compound **3**
E (Hartree) = -1097.477518

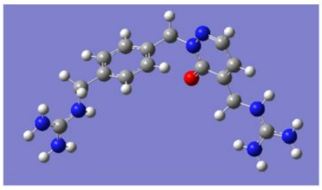

Compound **4**
E (Hartree) = -1097.478082

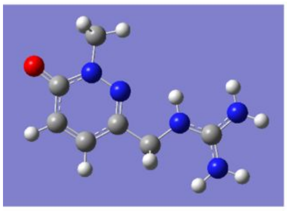

Compound **5**
E (Hartree) = -622.615278

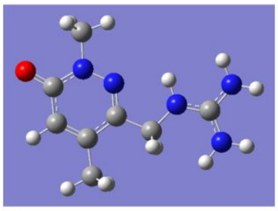

Compound **6**
E (Hartree) = -661.917902

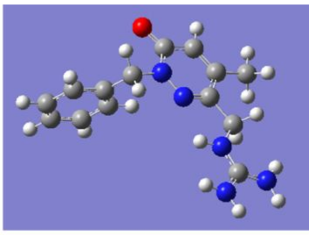

Compound **7**
E (Hartree) = -892.887432

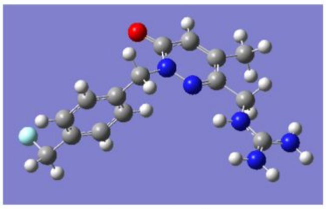

Compound **8**
E (Hartree) = -1031.395244

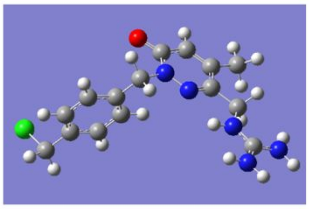

Compound **9**
E (Hartree) = -1391.756791

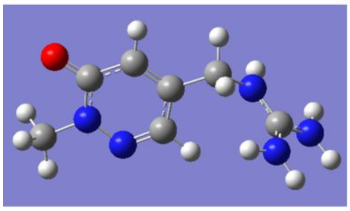

Compound **10**
E (Hartree) = -622.612587

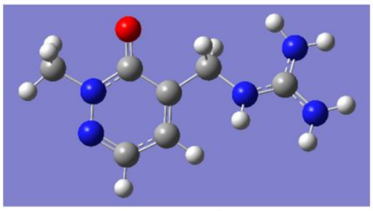

Compound **11**
E (Hartree) = -622.613456

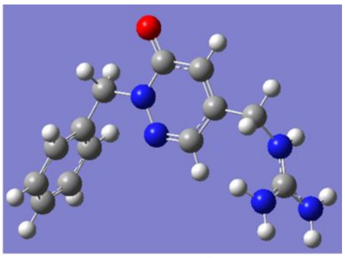

Compound **12**
E (Hartree) = -853.579658

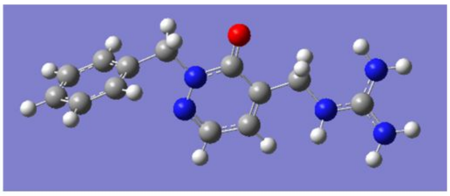

Compound **13**
E (Hartree) = -853.580237

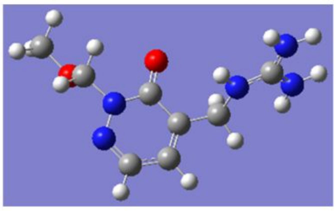

Compound **14**
E (Hartree) = -737.092870

Figure S1. Optimised structures of all compounds investigated using DFT (M06-2X, 6-31+G(d.p), SMD= water). Total energies in Hartrees are also indicated.

Compound 2

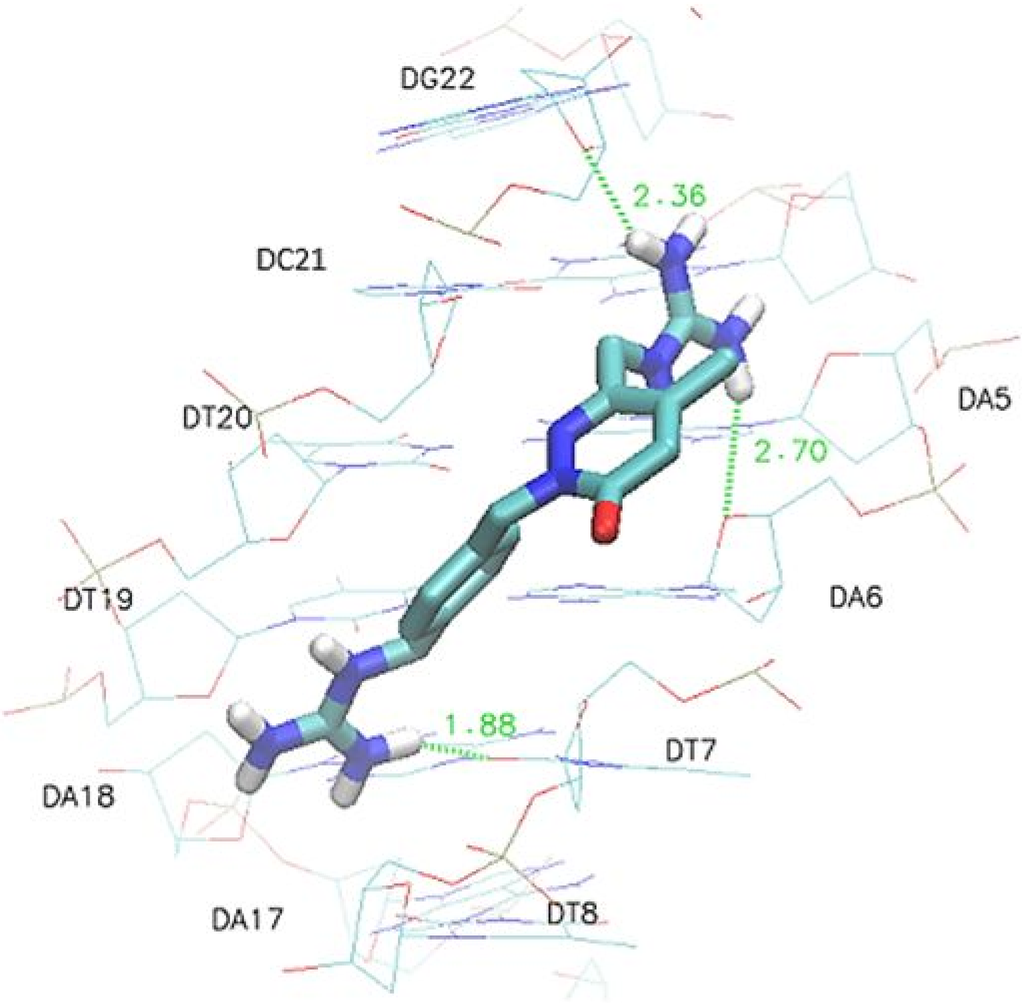

Figure S2.- Best pose obtained for the docking of compound **2** into the minor groove model (dodecanucleotide d(CGCGAATTCGCG)$_2$ PDB: 1D64).

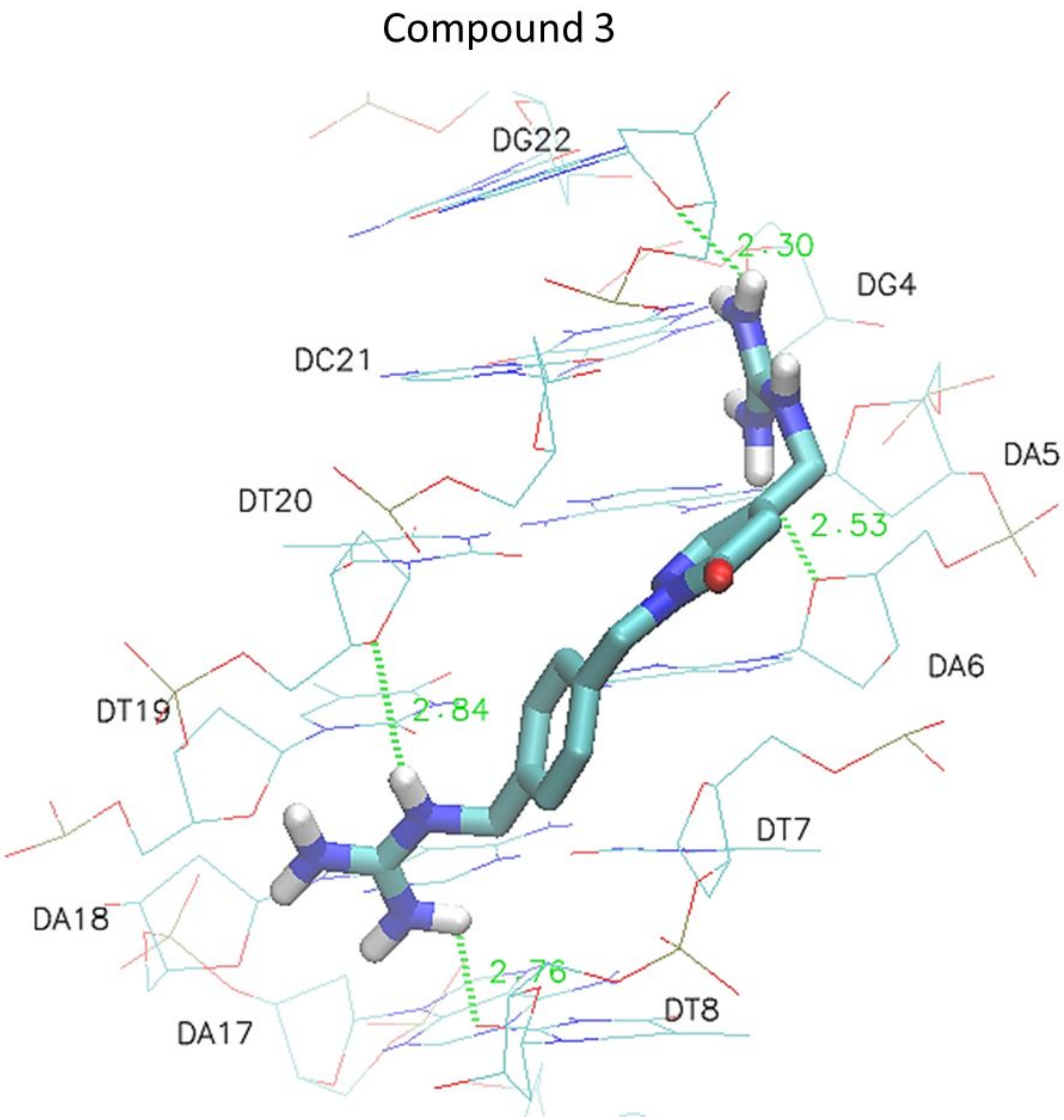

Figure S3.- Best pose obtained for the docking of compound **3** into the minor groove model (dodecanucleotide d(CGCGAATTCGCG)$_2$ PDB: 1D64).

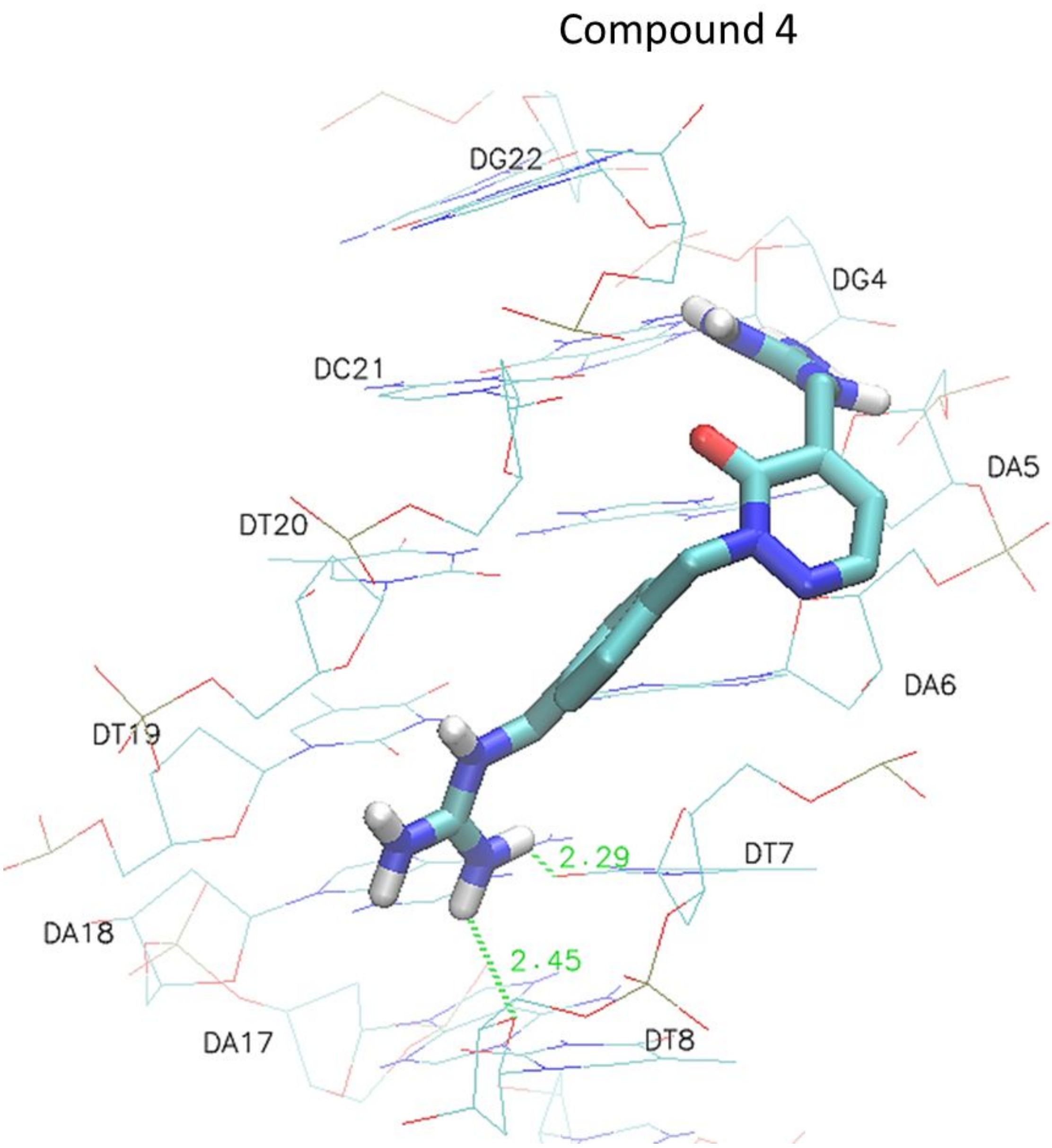

Figure S4.- Best pose obtained for the docking of compound **4** into the minor groove model (dodecanucleotide d(CGCGAATTCGCG)$_2$ PDB: 1D64).

Compound 5

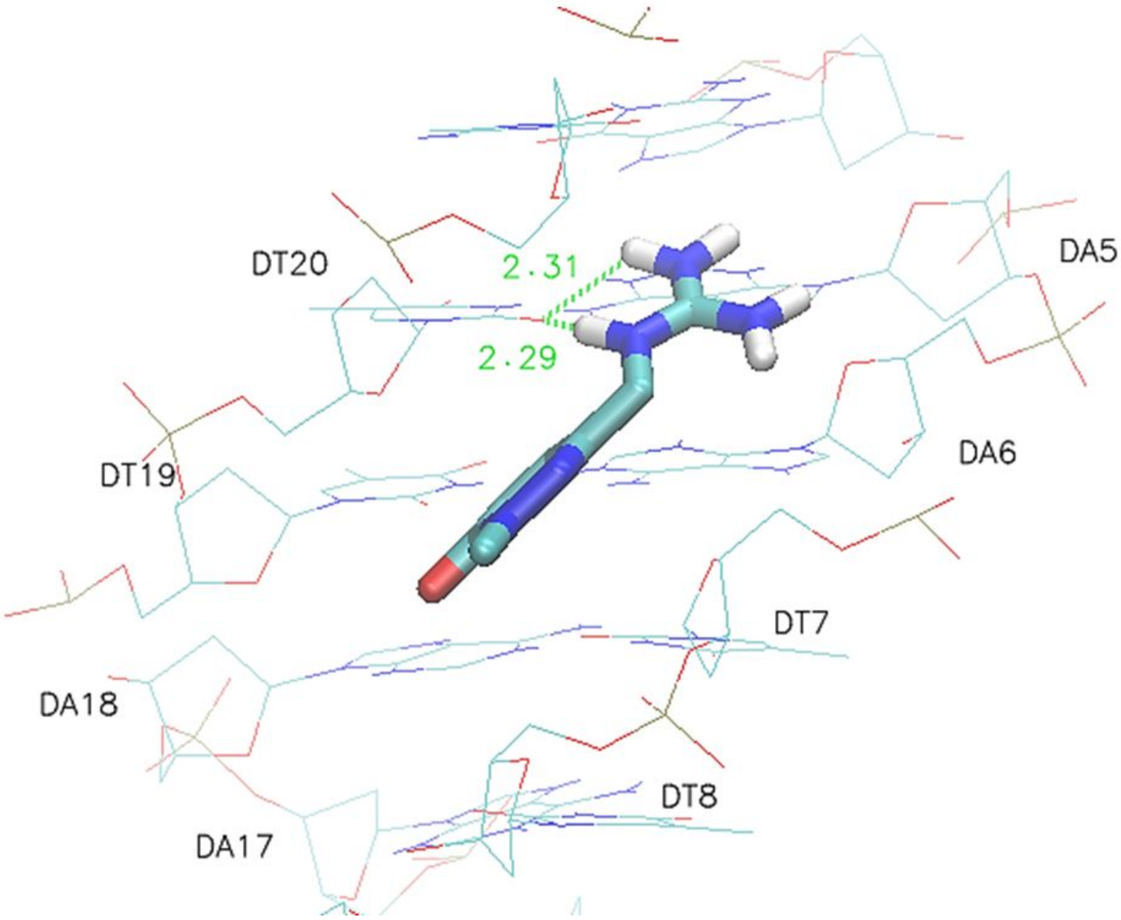

Figure S5.- Best pose obtained for the docking of compound **5** into the minor groove model (dodecanucleotide d(CGCGAATTCGCG)$_2$ PDB: 1D64).

Compound 6

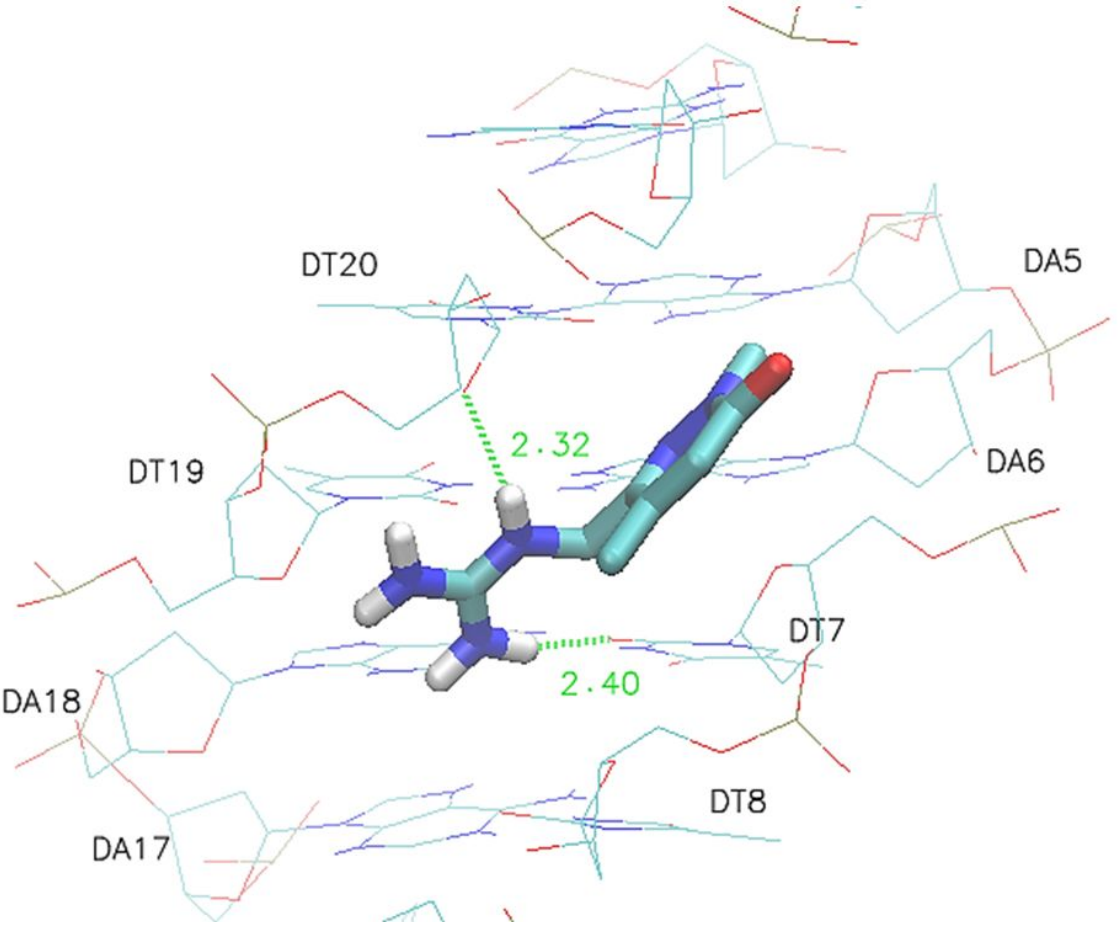

Figure S6.- Best pose obtained for the docking of compound **6** into the minor groove model (dodecanucleotide d(CGCGAATTCGCG)$_2$ PDB: 1D64).

Compound 7

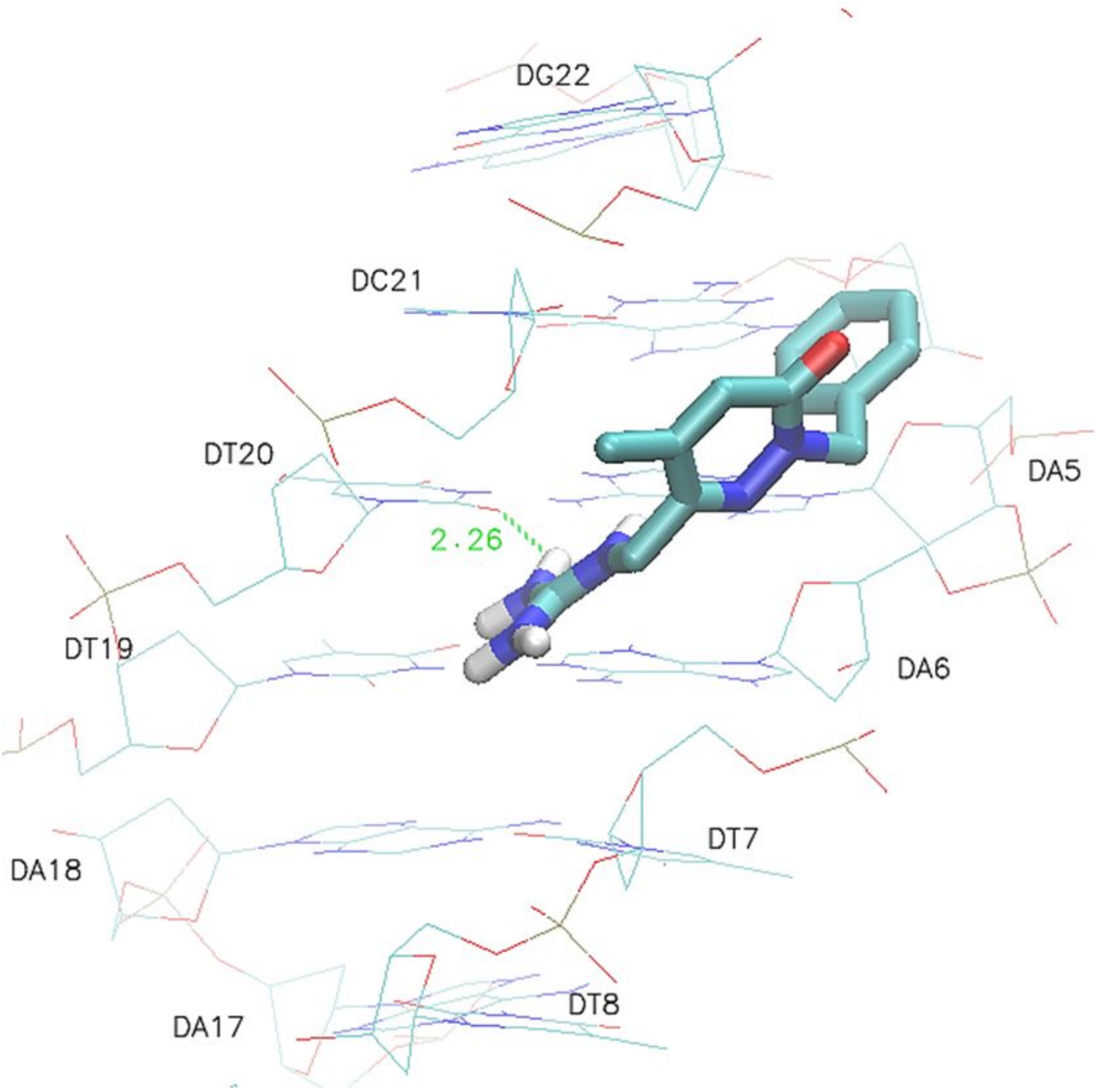

Figure S7.- Best pose obtained for the docking of compound **7** into the minor groove model (dodecanucleotide d(CGCGAATTCGCG)$_2$ PDB: 1D64).

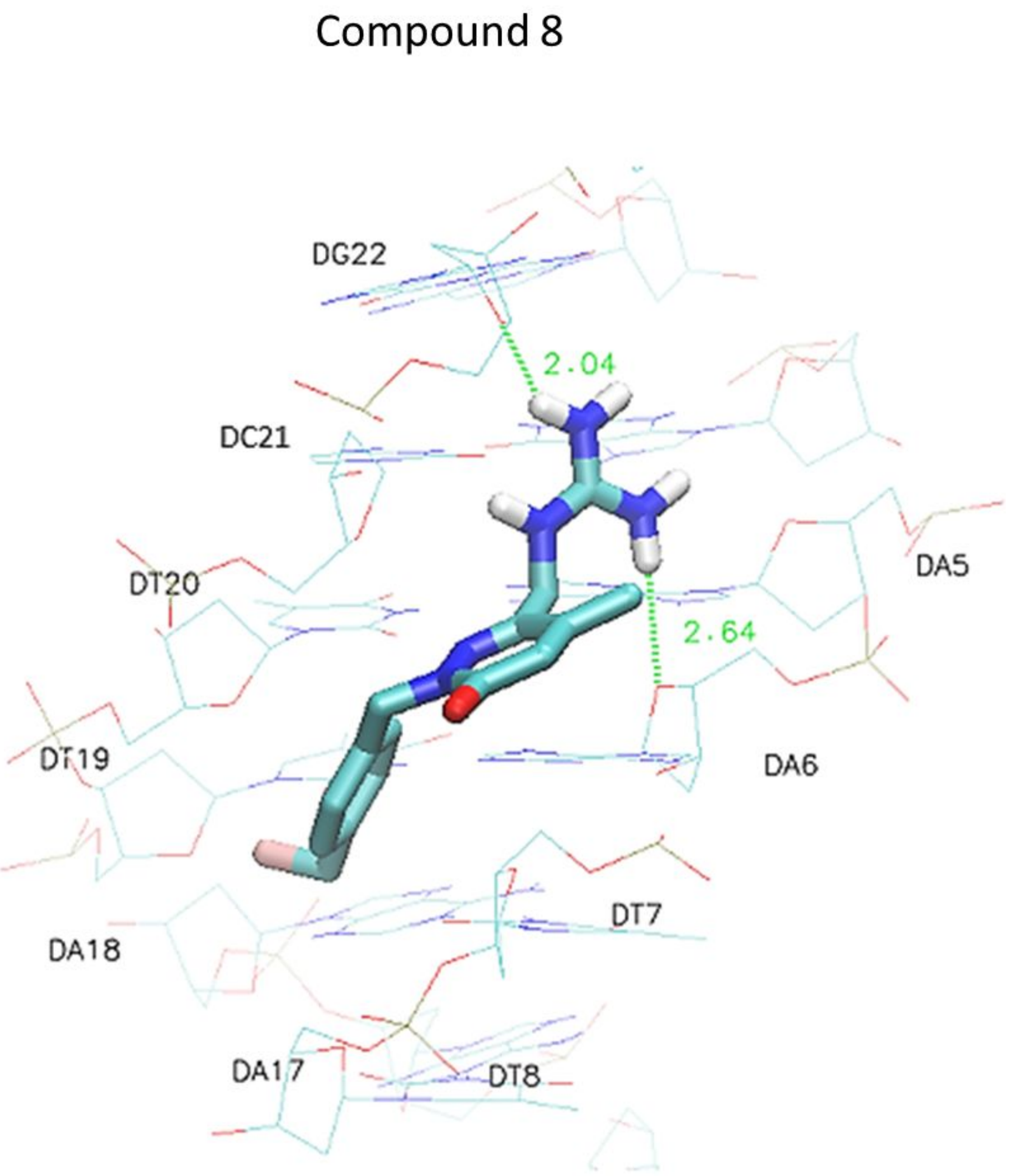

Figure S8.- Best pose obtained for the docking of compound **8** into the minor groove model (dodecanucleotide d(CGCGAATTCGCG)$_2$ PDB: 1D64).

Compound 9

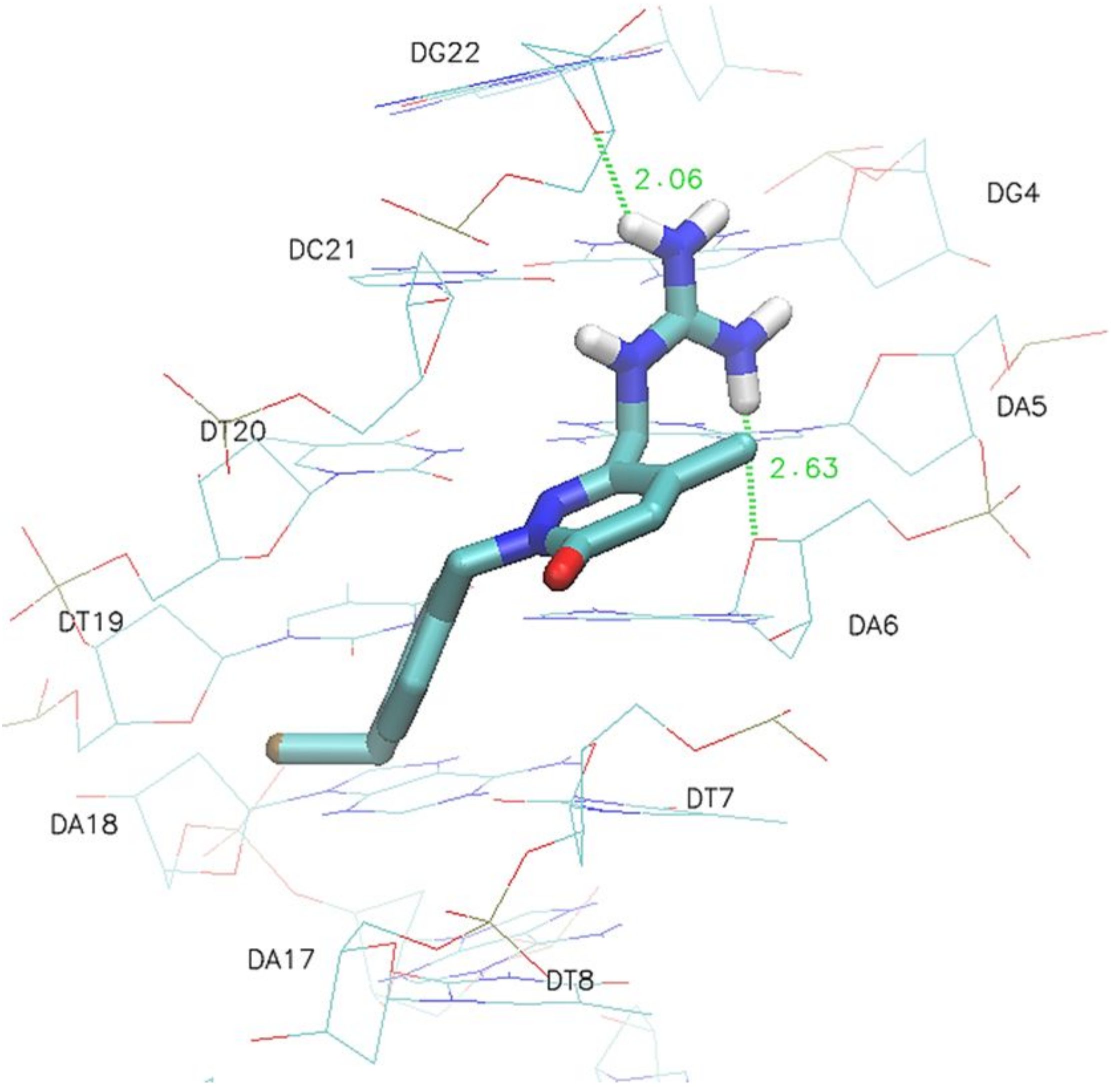

Figure S9.- Best pose obtained for the docking of compound **9** into the minor groove model (dodecanucleotide d(CGCGAATTCGCG)$_2$ PDB: 1D64).

Compound 10

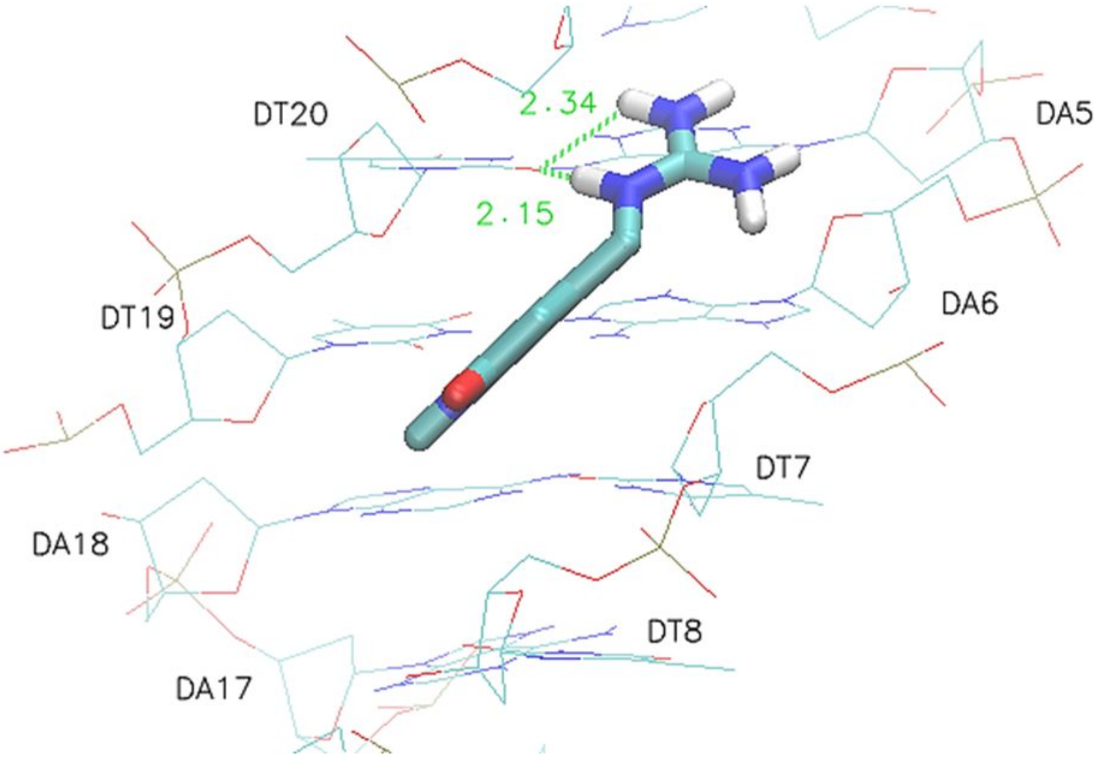

Figure S10.- Best pose obtained for the docking of compound **10** into the minor groove model (dodecanucleotide d(CGCGAATTCGCG)$_2$ PDB: 1D64).

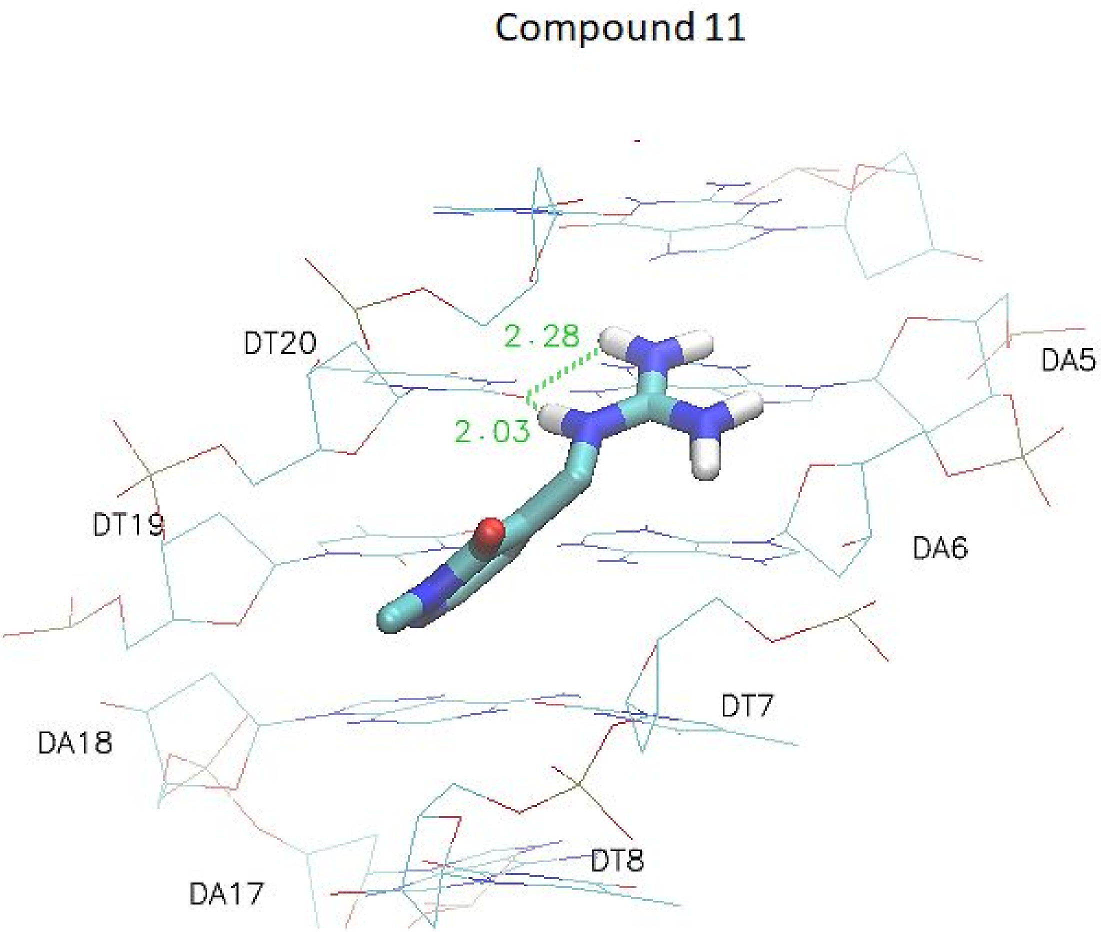

Figure S11.- Best pose obtained for the docking of compound **11** into the minor groove model (dodecanucleotide d(CGCGAATTCGCG)$_2$ PDB: 1D64).

Compound 12

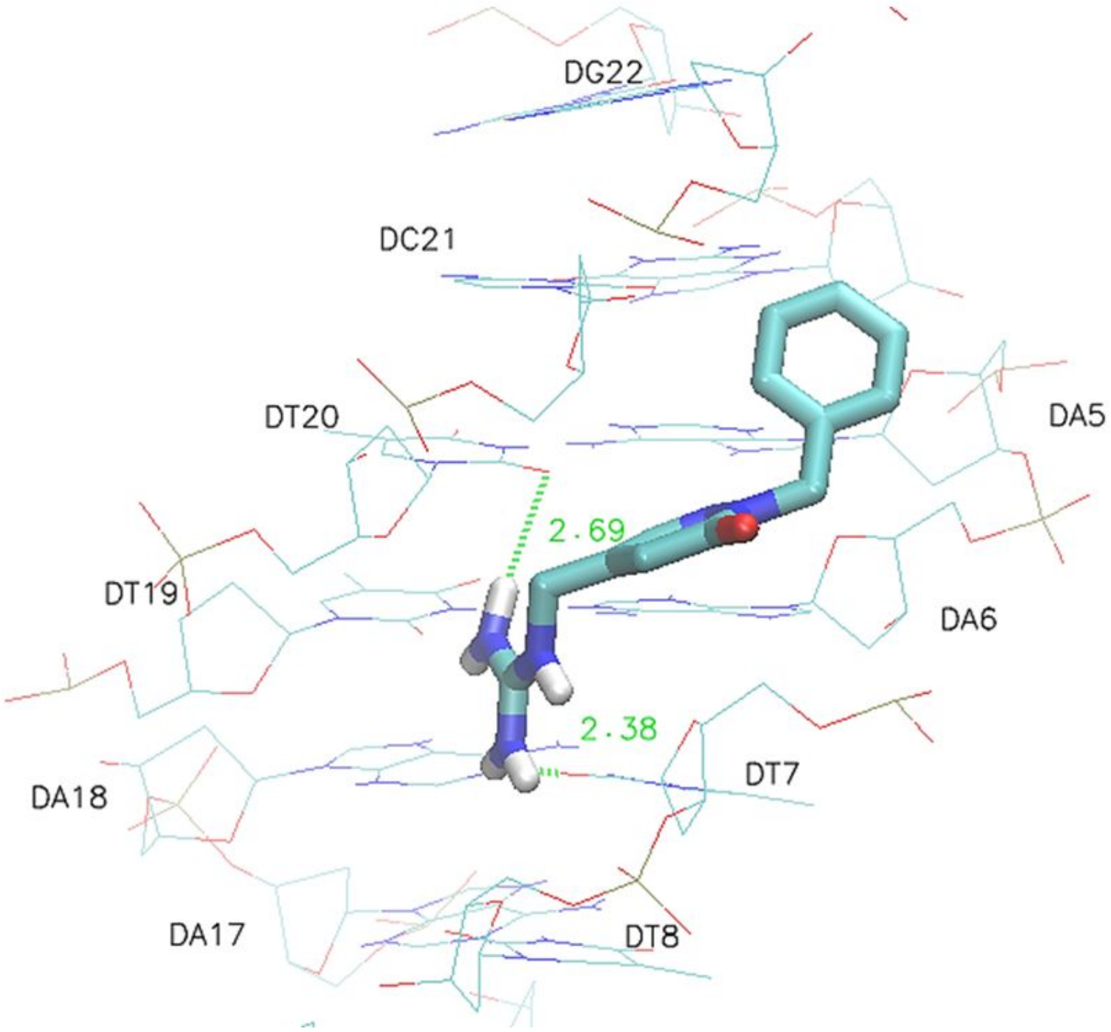

Figure S12.- Best pose obtained for the docking of compound **12** into the minor groove model (dodecanucleotide d(CGCGAATTCGCG)$_2$ PDB: 1D64).

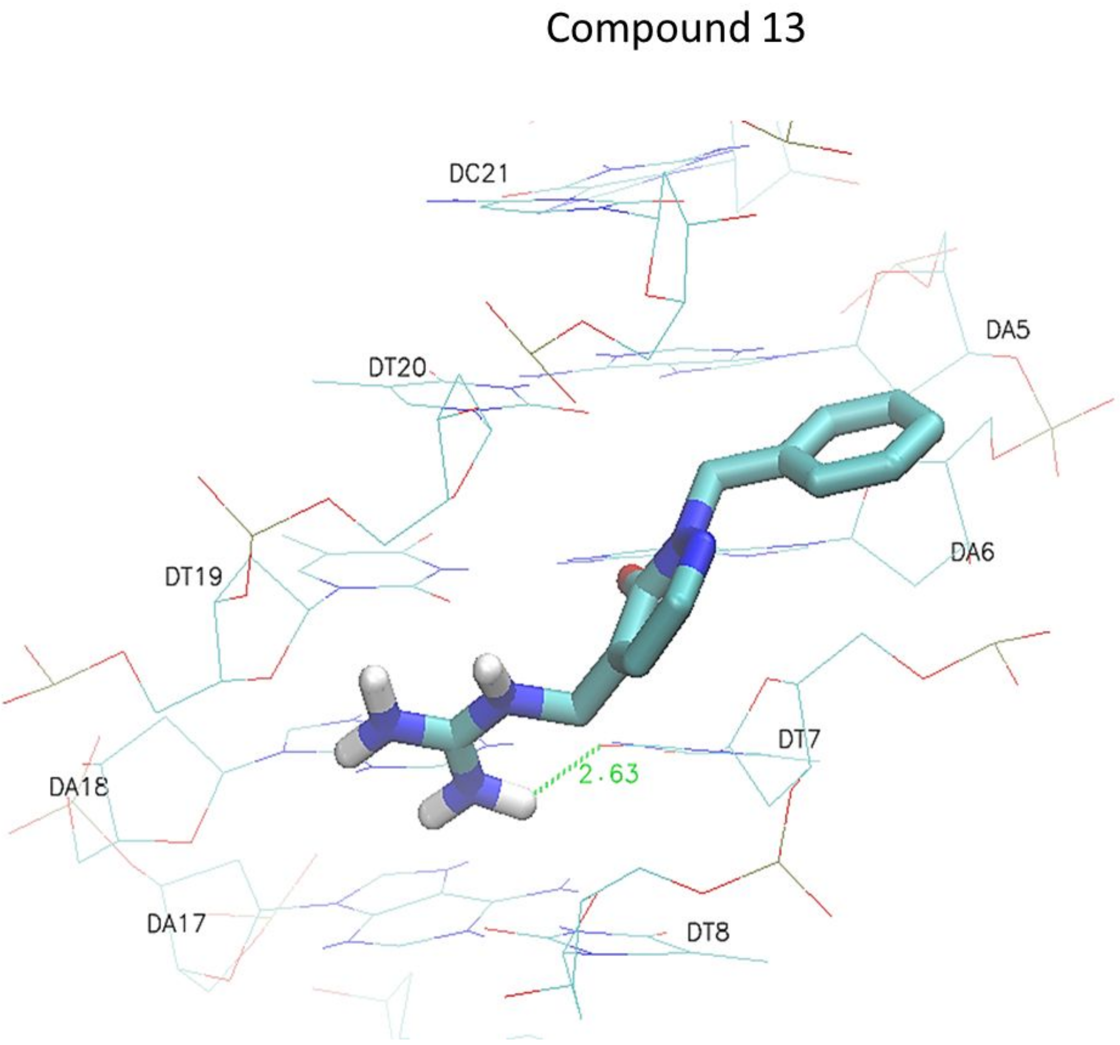

Figure S13.- Best pose obtained for the docking of compound **13** into the minor groove model (dodecanucleotide d(CGCGAATTCGCG)$_2$ PDB: 1D64).

Compound 14

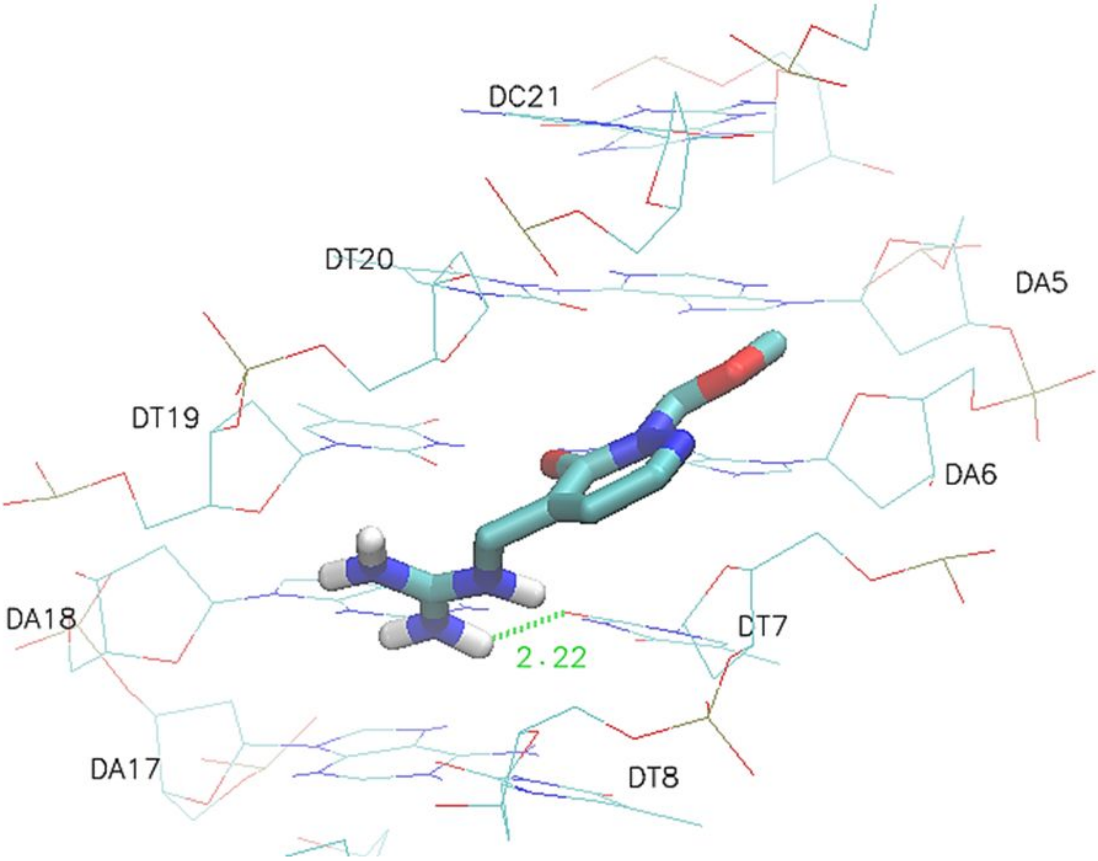

Figure S14.- Best pose obtained for the docking of compound **14** into the minor groove model (dodecanucleotide d(CGCGAATTCGCG)$_2$ PDB: 1D64).

Table S1.- Distances (Å), angles (°), atoms involved and qualitative strength of the HBs observed in the docking for all compounds studied; G-scores obtained are also indicated for each ligand-oligonucleotide complex.

Compound	H bond distance (Å)	H bond angle (°)	HBA	HBD	Strength	Gscore (kcal mol^{-1})
**1**	2.26	154.11	DG22:04	Gua-NH	weak	-8.9
	2.38	170.59	DA6:O4	Gua-NH	weak	
	2.55	142.9	DT20:O4	Gua-NH	weak	
	2.76	136.61	DT8:O4	Gua-NH	weak	
**2**	2.36	141.53	DG22:O4	Gua-NH	weak	-8
	2.7	157.2	DA6:O4	Gua-NH	weak	
	1.88	150.81	DT7:O2	Gua-NH	medium	
**3**	2.3	127.71	DG22:O4	Gua-NH	weak	-8.7
	2.53	144.83	DA6:O4	Gua-NH	weak	
	2.84	137.17	DT20:O4	Gua-NH	weak	
	2.76	108.66	DT8:O2	Gua-NH	weak	
**4**	2.29	129.5	DT7:O2	Gua-NH	weak	-7.7
	2.45	130.83	DT8:O4	Gua-NH	weak	
**5**	2.29	136.59	DT20:O2	Gua-NH	weak	-6.8
	2.31	130.21	DT20:O2	Gua-NH	weak	
**6**	2.32	125.37	DT20:O4	Gua-NH	weak	-6.1
	2.4	158.58	DT7:O2	Gua-NH	weak	
**7**	2.26	124.97	DT20:O2	Gua-NH	weak	-6.3
**8**	2.04	143.2	DG22:O4	Gua-NH	medium	-7.3
	2.64	177.08	DA6:O4	Gua-NH	weak	
**9**	2.06	140.24	DG22:O4	Gua-NH	medium	-7.1
	2.63	176.68	DA6:O4	Gua-NH	weak	
**10**	2.15	141.92	DT20:O2	Gua-NH	medium	-6.8
	2.34	130.73	DT20:O2	Gua-NH	weak	
**11**	2.03	147.9	DT20:O2	Gua-NH	medium	-6.6
	2.28	129.8	DT20:O2	Gua-NH	weak	
**12**	2.69	161.34	DT20:O2	Gua-NH	weak	-7.3
	2.38	126.63	DT7:O2	Gua-NH	weak	
**13**	2.63	121.51	DT7:O2	Gua-NH	weak	-7.2
**14**	2.22	135.77	DT19:O4	Gua-NH	weak	-6.3

Schemes of the preparation of scaffolds and precursors needed

Scheme S1. Synthesis of pyridazinone derivatives **25-35**[a]

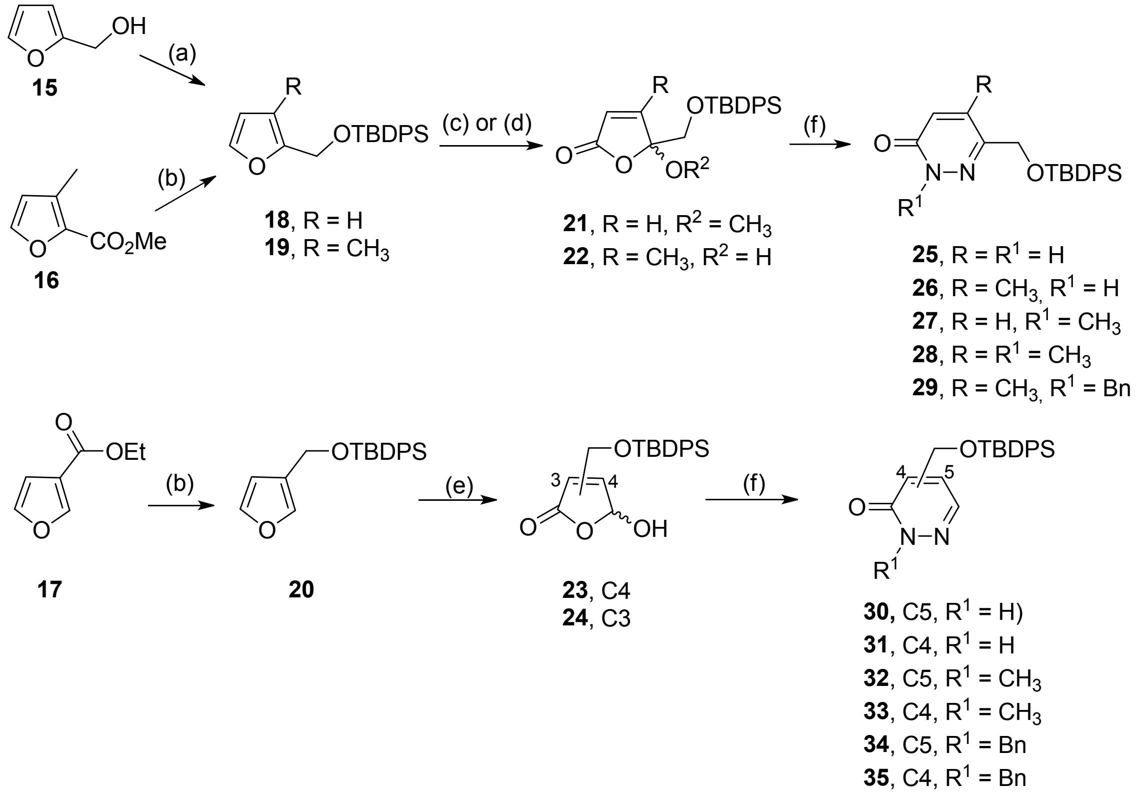

[a]Reagents and Conditions: (a) **15**,TBDPSCl, imidazole, DMF, r.t., 1.5 h (**18**); (b) **16** or **17** $LiAlH_4$, Et_2O, r.t., 45 min; TBDPSCl, imidazole, DMF, r.t., 1.5 h (**19** or **20**); (c) **18**, O_2, hv, rose bengal, MeOH, -78 °C, 5 h; Ac_2O, pyridine, DMAP, r.t., 20 h (**21**); (d) **19**, O_2, hv, rose bengal, MeOH, DIPEA, -78 °C, 4 h (**22**); (e) **20**, O_2, hv, rose bengal, MeOH, DBU, -78 °C, 2 h (**23** and **24**); (f) **21**, hydrazine monohydrate or methyl hydrazine, EtOH, reflux, 2 h (**25**, 14%), 3h (**27**); **22**, hydrazine monohydrate or methyl hydrazine or benzyl hydrazine dihydrochloride and TEA, ethanol, reflux, 2 h (**26**, 72%), 3 h (**28,** 52% or **29**, 66%); **23** and **24**, hydrazine monohydrate or methyl hydrazine or benzyl hydrazine dihydrochloride and TEA, ethanol, reflux, 4 h (**30** and **31**, 56% and 5% respectively from **20**), 7 h (**32**, 84% and **33**, 73%; **34** and **35**, 46% and 7% respectively from **20**).

Scheme S2. Synthesis of dibromo derivatives **48-51**[a]

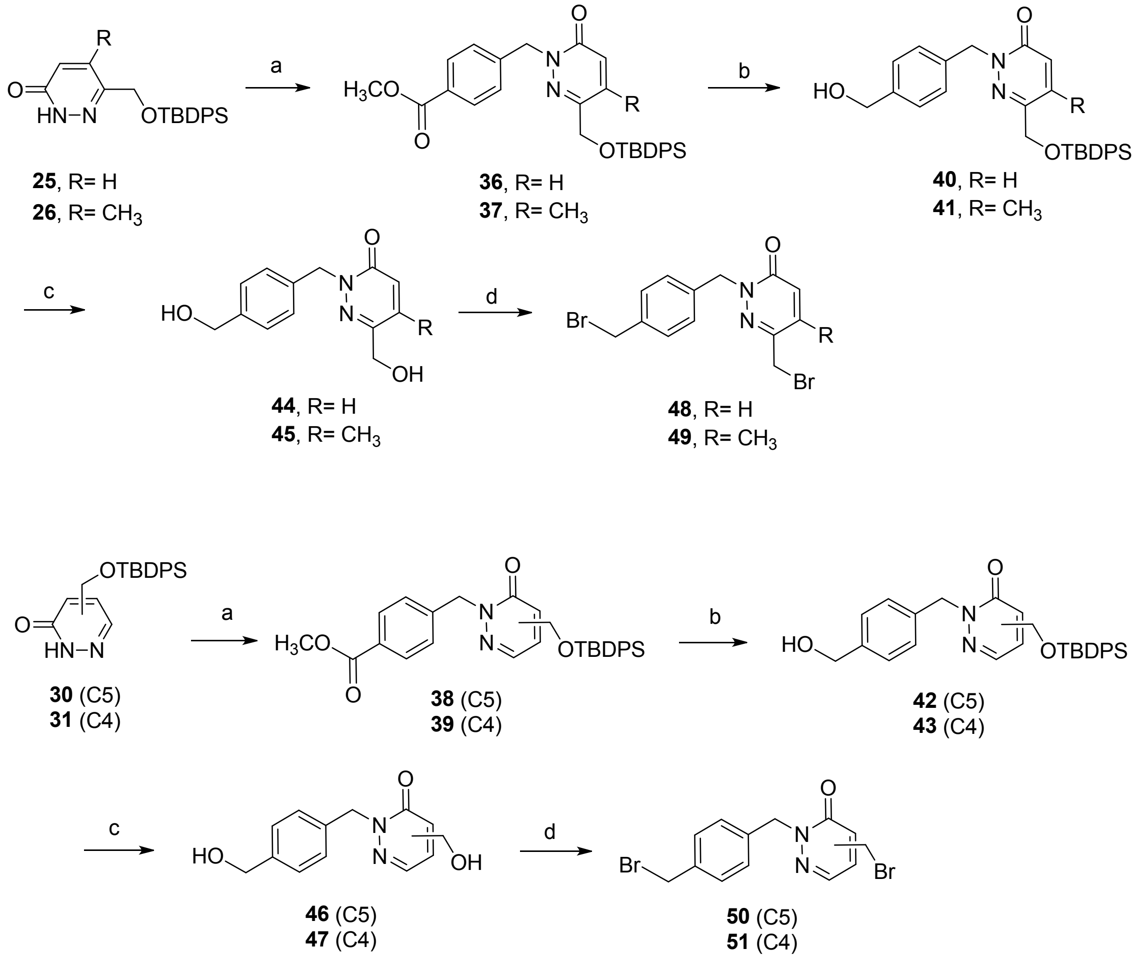

[a]Reagents and Conditions: (a) NaH 60% mineral oil, methyl 4-bromomethylbenzoate, DMF, r.t., 24 h; (b) DIBAL-H 1M hexane, DCM, -78 °C, 3 h; (c) TBAF 1M THF, r.t., 15 min.; (d) CBr_4, Ph_3P, DCM, reflux, 3 h, **48** (65%), **49** (67%), **51** (98%) and 6 h **50** (90%).

Scheme S3. Synthesis of bromo derivatives **63-69**[a]

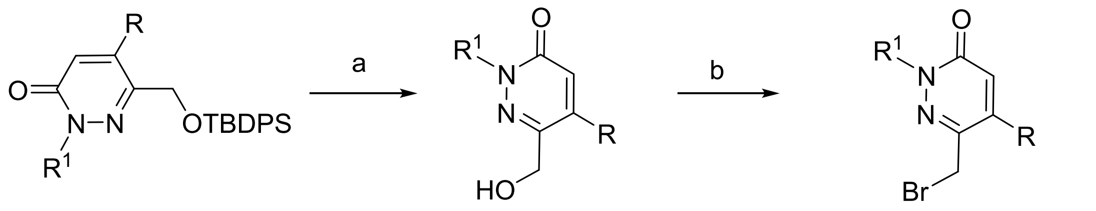

27, R= H, R^1= CH_3
28, R = R^1= CH_3
29, R = CH_3, R^1= Bn)

56, R= H, R^1= CH_3
57, R = R^1= CH_3
58, R = CH_3, R^1= Bn

63 R= H, R^1= CH_3
64 R = R^1= CH_3
65 R = CH_3, R^1= Bn

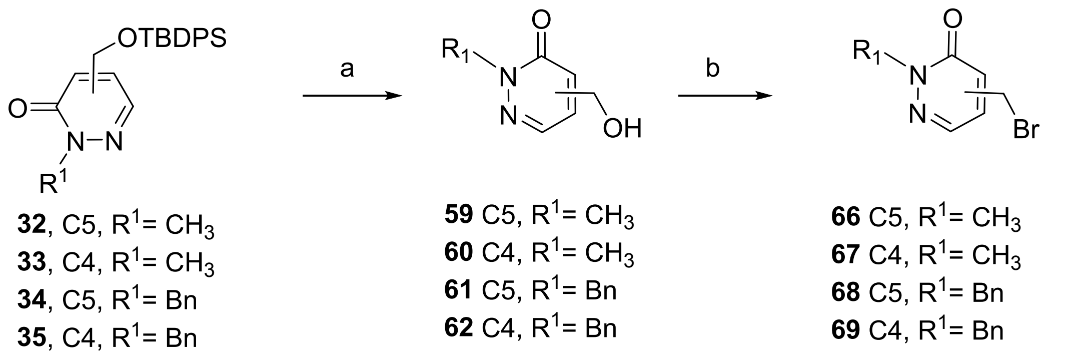

32, C5, R^1= CH_3
33, C4, R^1= CH_3
34, C5, R^1= Bn
35, C4, R^1= Bn

59 C5, R^1= CH_3
60 C4, R^1= CH_3
61 C5, R^1= Bn
62 C4, R^1= Bn

66 C5, R^1= CH_3
67 C4, R^1= CH_3
68 C5, R^1= Bn
69 C4, R^1= Bn

[a]Reagents and Conditions: (a) TBAF 1M THF, r.t., 15 min.; (b) CBr_4, Ph_3P, DCM, reflux, 1.5 h **63** (80%), **64** (96%), **65** (76%), **66** (82%), **67** (85%,), **68** (73%), **69** (84%).

Scheme S4. Synthesis of bromo derivatives **74-75**[a]

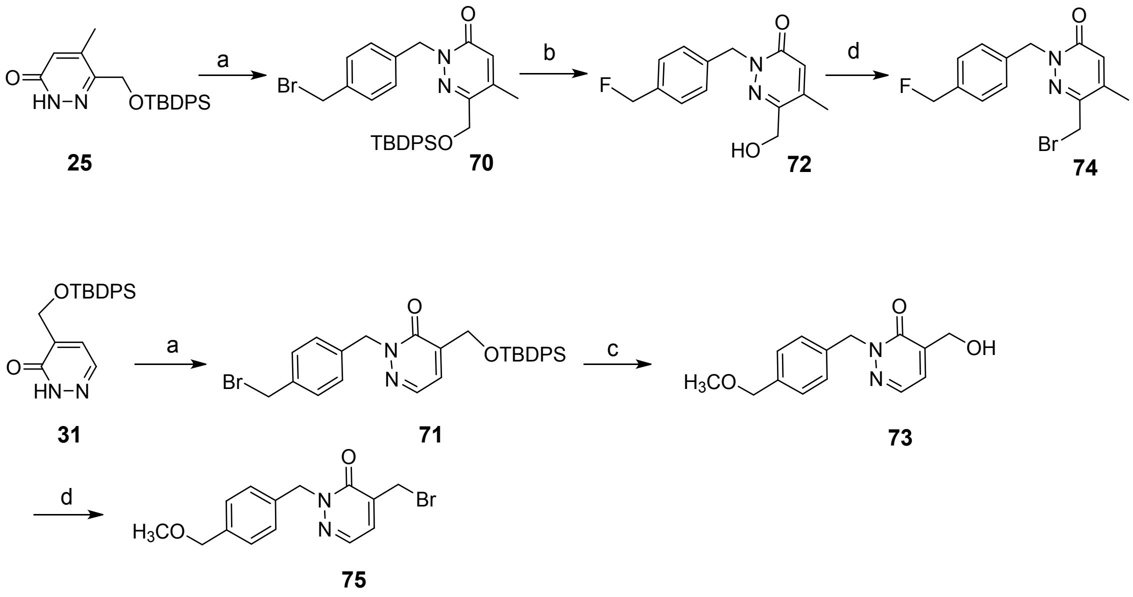

[a]Reagents and Conditions: (a) NaH 60% in mineral oil, α,α'-dibromo-*p*-xylene, Bu_4NI, THF, r.t., 5h; (b) TBAF 1M THF, r.t., 15 min; (c) TMSBr, MeOH, reflux, 9 h; (d) CBr_4, Ph_3P, DCM, reflux, 3 h **74** (70%), **75** (94%).

Synthesis of compounds 1-14

General methods

The starting chemical materials were of commercial sources and used as provided. The solvents were purified and dried following standard protocols. Air-sensitive reactions were performed under an argon atmosphere. Melting points were measured in a Stuart Scientific SMP3 or SMP10 instrument by means of capillary tubes. ^{1}H NMR, ^{13}C NMR and ^{19}F NMR spectra were registered on a Bruker (ARX400) spectrometer with TMS as internal reference. Chemical shifts (õ) were indicated in ppm and coupling constants in Hertz (Hz). COSY, DEPT and HSQC analysis were carried out to unequivocally assign the different signals. High resolution mass spectra were registered on a Bruker microTOF focus spectrometer using the electrospray ionization technique.

Analytical thin layer chromatography (TLC) was carried out on pre-coated aluminium sheets with silica gel (Merck 60 F254, 0.25 mm). Flash chromatography (FC) was performed on silica gel (Merck 60, 230–400 mesh). Reverse phase chromatography was performed by using reversed phase silica gel 100 C_8 (Sigma Aldrich, ≥400 mesh).
HPLC purity analysis of guanidine derivatives were carried out using a Jasco PU-980 system equipped with a UV-975 Intelligent UV/VIS detector and a manual injector (20 µL). For purity assessment, UV detection was performed at 284 nm and peak purity was confirmed by standard normalization. The stationary phase consisted of a Kinetex 5 µm C18 100Å column (250×4.6 mm), and the mobile phases used were 20% methanol buffered (aqueous formate buffer) and 40% methanol buffered eluting at 1 mL/min. Minimum requirement for purity was set at 94.0%. Synthesis of silyl protected hydroxyalkylpyridazin-3(2H)-ones **25**, **27**, **30-35** as well as of hydroxyalkyl (**56**, **57**, **59-62**) and bromoalkyl analogues (**63**, **64**, **66-69**) was performed as previously was described [5,6,7].

6-(*tert*-Butyldiphenylsylyloxymethyl)-5-methylpyridazin-3(2H)-one (26). A solution of **22** [7] (800 mg, 2.09 mmol) and hydrazine monohydrate (20 µL, 4.20 mmol) in EtOH (10 mL) was stirred at reflux for 2 h. After the solvent was removed, the residue was purified by column chromatography on silica gel (50% EtOAc/hexane) to afford **26** (570 mg, 72%, white solid). R_f = 0.4 (50% EtOAc/hexane); m.p. = 149 – 150 ºC; ^{1}H NMR ($CDCl_3$): δ = 10.81 (s, 1H, NH), 7.69 - 7.65 (m, 4H, H-Ph), 7.50 - 7.45 (m, 2H, H-Ph), 7.44 - 7.38 (m, 4H, H-Ph), 6.74 (d, 1H, J = 1.2 Hz, H4), 4.63 (s, 2H, CH_2), 2.38 (d, 3H, J

= 1.2 Hz, CH_3), 1.07 (s, 9H, $(CH_3)_3$); ^{13}C NMR ($CDCl_3$): δ = 162.7 (C3), 147.0 (C6), 145.4 (C5), 135.7 (CH-Ph), 132.8 (C-Ph), 130.0 (CH-Ph), 128.4 (C4), 127.9 (CH-Ph), 64.5 (CH_2), 26.9 ($(CH_3)_3$), 19.4 ($C(CH_3)_3$), 18.6 (CH_3); HRMS (ESI): *m/z* $[M+H]^+$ calcd for $C_{22}H_{27}N_2O_2Si$, 379.18363; found 379.18308.

6-(*tert*-Butyldiphenylsylyloxymethyl)-2,5-dimethylpyridazin-3(2H)-one (28). To a solution of **22** [7] (250 mg, 0.65 mmol) in EtOH (10 mL) was added methylhydrazine (52 µL, 0.98 mmol) and the reaction mixture was refluxed under stirring for 3 h. The solvent was removed and the residue was purified by column chromatography on silica gel (20% EtOAc/hexane) to yield **28** (133 mg, 52%) as a yellowish oil. R_f = 0.3 (50% EtOAc/hexane); ^{1}H NMR ($CDCl_3$): δ = 7.66 - 7.63 (m, 4H, H-Ph), 7.47 - 7.42 (m, 2H, H-Ph), 7.41 - 7.35 (m, 4H, H-Ph), 6.68 (d, 1H, J = 1.1 Hz, H4), 4.62 (s, 2H, CH_2), 3.60 (s, 3H, CH_3N), 2.31 (d, 3H, J = 1.1 Hz, CH_3), 1.05 (s, 9H, $(CH_3)_3$); ^{13}C NMR ($CDCl_3$): δ = 160.8 (C3), 145.7, 143.6, 135.7 (CH-Ph), 132.9 (C-Ph), 129.9 (CH-Ph), 128.0 (C4), 127.7 (CH-Ph), 64.4 (CH_2), 39.5 (CH_3N), 26.7 ($(CH_3)_3$), 19.3 ($C(CH_3)_3$), 17.9 (CH_3); HRMS (ESI): *m/z* $[M+H]^+$ calcd for $C_{23}H_{29}N_2O_2Si$, 393.19928; found 393.19770.

2-Benzyl-6-(*tert*-butyldiphenylsililoximethyl)-5-methylpyridazin-3(2*H*)-one (29). To a solution of **22** [7] (283 mg, 0.74 mmol) in EtOH (10 mL) was added benzylhydrazine dihydrochloride (216 mg, 1.11 mmol) and Et_3N (0.3 mL, 2.22 mmol) and the reaction mixture was stirred at reflux for 3 h. After the solvent was removed, CH_2Cl_2 (10 mL) and H_2O (10 mL) were added and the mixture was extracted with CH_2Cl_2 (3x10 mL). The combined organic phases were dried over Na_2SO_4, filtered and the solvent was evaporated to dryness. The residue was purified by column chromatography on silica gel (20% EtOAc/hexane) to afford **29** (146 mg, 66%) as a colourless oil. R_f = 0.5 (50% EtOAc/hexane); ^{1}H NMR ($CDCl_3$): δ = 7.66 – 7.61 (m, 4H, H-Ph), 7.45 – 7.40 (m, 2H, H-Ph), 7.37 – 7.32 (m, 6H, H-Ph), 7.30 – 7.21 (m, 3H, H-Ph), 6.69 (d, 1H, J = 1.1 Hz, H4), 5.17 (s, 2H, CH_2N), 4.62 (s, 2H, CH_2), 2.29 (d, 3H, J = 1.1 Hz, CH_3), 1.02 (s, 9H, $(CH_3)_3$); ^{13}C NMR ($CDCl_3$): δ = 160.4 (C3), 146.0, 143.6, 136.5 (C-Ph), 135.7 (CH-Ph), 133.0 (C-Ph), 129.9 (CH-Ph), 128.7, 128.6, 128.5, 127.8 (CH-Ph), 127.8 (CH-Ph), 64.5 (CH_2), 54.6 (CH_2N), 26.8 ($(CH_3)_3$), 19.3 ($C(CH_3)_3$), 18.0 (CH_3); HRMS (ESI): *m/z* $[M+H]^+$ calcd for $C_{29}H_{33}N_2O_2Si$, 469.23058; found 469.23051.

General procedure to synthesize methyl esters 36-39. A solution of compound **25-26** or **30-31** (0.25 mmol) in DMF (2 mL) was added, dropwise at 0 °C, to a suspension of NaH (0.38 mmol, 60% dispersion in mineral oil) in DMF (2 mL). After the mixture was stirred at room temperature for 1 h, methyl 4-(bromomethyl)benzoate (0.38 mmol) was added. The reaction mixture was stirred at room temperature for 24 h, followed by quenching with MeOH. The solvent was evaporated to dryness, and the residue was purified by column chromatography on silica gel to afford the desired compound.

2-[(4-methoxycarbonyl)benzyl]-6-(*tert*-butyldiphenylsililoximethyl)pyridazin-3(2*H*)-one (36). Compound **36** was purified by column chromatography on silica gel (40% EtOAc/hexane). Yellowish oil; yield 76%; R_f = 0.5 (50% EtOAc/hexane); ^{1}H NMR ($CDCl_3$): δ = 7.99 – 7.94 (m, 2H, H-Ph), 7.66 – 7.61 (m, 4H, H-Ph), 7.46 – 7.34 (m, 9H, H5, H-Ph), 6.95 (d, 1H, J = 9.5 Hz, H4), 5.25 (s, 2H, CH_2N), 4.60 (s, 2H, CH_2O), 3.89 (s, 3H, CH_3O), 1.08 (s, 9H, $(CH_3)_3$); ^{13}C NMR ($CDCl_3$): δ = 166.9 (*C*OOCH$_3$), 160.0 (C3), 147.1 (C6), 141.3 (C-Ph), 135.6 (CH-Ph), 132.8 (C-Ph), 131.2 (C5), 130.5 (C4), 130.1 (CH-Ph), 129.9 (CH-Ph), 129.7 (C-Ph), 128.6 (CH-Ph), 128.0 (CH-Ph), 64.5 (CH_2O), 54.8 (CH_2N), 52.2 (CH_3O), 26.9 (($CH_3)_3$), 19.3 (*C*($CH_3)_3$); HRMS (ESI): *m/z* $[M+H]^+$ calcd for $C_{30}H_{33}N_2O_4Si$, 513.22041; found 513.21841.

2-[(4-methoxycarbonyl)benzyl]-6-(*tert*-butyldiphenylsililoximethyl)-5-methylpyridazin-3(2*H*)-one (37). Compound **37** was purified by column chromatography on silica gel (50% EtOAc/hexane). Colourless oil; yield 96%; R_f = 0.3 (50% EtOAc/hexane); ^{1}H NMR ($CDCl_3$): δ = 7.98 – 7.93 (m, 2H, H-Ph), 7.65 – 7.60 (m, 4H, H-Ph), 7.47 – 7.31 (m, 8H, H-Ph), 6.71 (d, 1H, J = 1.1 Hz, H4), 5.21 (s, 2H, CH_2N), 4.63 (s, 2H, CH_2O), 3.89 (s, 3H, CH_3O), 2.33 (d, 3H, J = 1.1 Hz, CH_3), 1.04 (s, 9H, $(CH_3)_3$); ^{13}C NMR($CDCl_3$): δ = 166.9 (*C*OOCH$_3$), 160.3 (C3), 146.3, 143.8, 141.5 (C-Ph), 135.7 (CH-Ph), 133.0 (C-Ph), 130.0 (CH-Ph), 129.9 (CH-Ph), 129.6 (C-Ph), 128.7, 128.5, 127.8 (CH-Ph), 64.5(CH_2O), 54.3 (CH_2N), 52.2 (CH_3O) 26.9 (($CH_3)_3$), 19.3 (*C*($CH_3)_3$), 18.0 (CH_3); HRMS (ESI): *m/z* $[M+H]^+$ calcd for $C_{31}H_{35}N_2O_4Si$, 527.23606; found 527.23528.

2-[(4-methoxycarbonyl)benzyl]-5-(*tert*-butyldiphenylsililoximethyl)pyridazin-3(2*H*)-one (38). Compound **38** was purified by column chromatography on silica gel (20% EtOAc/hexane). Colourless oil; yield 80%; R_f = 0.4 (1:2 EtOAc/hexane); ^{1}H NMR

($CDCl_3$): δ = 8.02 – 7.98 (m, 2H, H-Ph), 7.67 – 7.60 (m, 5H, H6, H-Ph), 7.49 – 7.34 (m, 8H, H-Ph), 6.98 (dd, 1H, J = 3.6, 1.6 Hz, H4), 5.36 (s, 2H, CH_2N), 4.56 (d, 2H, J = 1.5 Hz, CH_2O), 3.90 (s, 3H, CH_3O), 1.09 (s, 9H, $(CH_3)_3$); ^{13}C NMR ($CDCl_3$): δ = 166.9 (*C*$OOCH_3$), 160.6 (C3), 145.9 (C5), 141.4 (C-Ph), 135.6 (C6, CH-Ph), 132.4 (C-Ph), 130.3 (CH-Ph), 130.0 (CH-Ph), 129.8 (C-Ph), 128.6 (CH-Ph), 128.1 (CH-Ph), 124.8 (C4), 61.9 (CH_2O), 54.6 (CH_2N), 52.2 (CH_3O), 26.9 ($(CH_3)_3$), 19.4 (*C*$(CH_3)_3$); HRMS (ESI): *m/z* $[M+H]^+$ calcd for $C_{30}H_{33}N_2O_4Si$, 513.22041; found 513.22058.

2-((4-Methoxycarbonyl)benzyl)-4-(*tert*-butyldiphenylsililoximethyl)pyridazin-3(2*H*)-one (39). Compound **39** was purified by column chromatography on silica gel (10% EtOAc/hexane). Colourless oil; yield 84%; R_f = 0.5 (50% EtOAc/hexane); ^1H NMR ($CDCl_3$): δ = 8.01 – 7.96 (m, 2H, H-Ph), 7.86 (d, 1H, J = 4.0 Hz, H6), 7.66 – 7.61 (m, 4H, H-Ph), 7.53 (dt, 1H, J = 4.0, 1.8 Hz, H5), 7.46 – 7.33 (m, 8H, H-Ph), 5.34 (s, 2H, CH_2N), 4.74 (d, 2H, J = 1.8 Hz, CH_2O), 3.89 (s, 3H, CH_3O), 1.13 (s, 9H, $(CH_3)_3$); ^{13}C NMR ($CDCl_3$): δ = 166.9 (*C*$OOCH_3$), 160.6 (C3), 145.9 (C4), 141.4 (C-Ph), 135.6 (C6), 135.6 (CH-Ph), 132.4 (C-Ph), 130.3 (CH-Ph), 130.0 (CH-Ph), 129.8 (C-Ph), 128.6 (CH-Ph), 128.1 (CH-Ph), 124.8 (C5), 61.9 (CH_2N), 54.6 (CH_2O), 52.2 (CH_3O), 26.9 ($(CH_3)_3$), 19.4 (*C*$(CH_3)_3$); HRMS (ESI): *m/z* $[M+H]^+$ calcd for $C_{30}H_{33}N_2O_4Si$, 513.22041; found 513.21980.

General procedure to synthesize compounds 40-43. To a solution of compound **36-39** (0.15 mmol) in CH_2Cl_2 (5 mL) at -78 °C was added dropwise a solution of DIBAL-H 1M in hexane (0.78 mmol). The reaction mixture was stirred at this temperature for 3 h, quenched with ^{t}BuOMe (0.40 mL), H_2O (60 µL) and NaOH 4M (30 µL) and stirred overnight at room temperature. The resulting white precipitate was filtered off, the solvent was evaporated under reduce pressure and the residue was purified by column chromatography to afford the desired compound.

2-[(4-hydroxymethyl)benzyl]-6-(*tert*-butyldiphenylsililoximethyl)pyridazin-3(2*H*)-one (40). Compound **40** was purified by column chromatography on silica gel (60% EtOAc/hexane). Colourless oil; yield 76%; R_f = 0.2 (50% EtOAc/hexane); ^1H NMR ($CDCl_3$): δ = 7.67 – 7.62 (m, 4H, H-Ph), 7.48 – 7.26 (m, 11H, H5, H-Ph), 6.91 (d, 1H, J = 9.5 Hz, H4), 5.20 (s, 2H, CH_2N), 4.68 – 4.61 (m, 3H, *CH_2*OH, OH), 4.60 (s, 2H, CH_2OSi), 1.08 (s, 9H, $(CH_3)_3$); ^{13}C NMR ($CDCl_3$): δ = 160.1 (C3), 147.0 (C6), 140.8 (C-

Ph), 135.6 (CH-Ph), 132.9 (C-Ph), 131.1 (C5), 130.4 (C4), 130.1 (CH-Ph), 129.0, 128.0, 127.2, 65.0 (CH_2OH), 64.5 (CH_2OSi), 55.0 (CH_2N), 26.9 ($(CH_3)_3$), 19.3 ($\mathit{C}(CH_3)_3$); HRMS (ESI): *m/z* $[M+H]^+$ calcd for $C_{29}H_{33}N_2O_3Si$, 485.22550; found 485.22381.

2-[(4-hydroxymethyl)benzyl]-6-(*tert*-butyldiphenylsililoximethyl)-5-methylpyridazin-3(2*H*)-one (41). Compound **41** was purified by column chromatography on silica gel (60% EtOAc/hexane). Colourless oil; yield 70%; $R_f = 0.2$ (50% EtOAc/hexane); ^1H NMR ($CDCl_3$): $\delta = 7.67 - 7.61$ (m, 4H, H-Ph), 7.48 – 7.41 (m, 2H, H-Ph), 7.41 – 7.23 (m, 8H, H-Ph), 6.67 (d, 1H, $J = 1.1$ Hz, H4), 5.15 (s, 2H, CH_2N), 4.65- 4.61 (m, 4H, $\mathit{CH_2}OH$, CH_2OSi), 2.39 (br s, 1H, OH), 2.30 (d, 3H, $J = 1.1$ Hz, CH_3), 1.04 (s, 9H, $(CH_3)_3$); ^{13}C NMR ($CDCl_3$): $\delta = 160.4$ (C3), 146.1, 143.6, 140.8 (C-Ph), 135.7 (CH-Ph), 135.7 (C-Ph), 133.0 (C-Ph), 130.0 (CH-Ph), 128.9 (CH-Ph), 128.6 (C4), 127.8 (CH-Ph), 127.2 (CH-Ph), 65.0 (CH_2OH), 64.5 (CH_2OSi), 54.5 (CH_2N), 26.9 ($(CH_3)_3$), 19.4 ($\mathit{C}(CH_3)_3$), 18.0 (CH_3); HRMS (ESI): *m/z* $[M+H]^+$ calcd for $C_{30}H_{35}N_2O_3Si$, 499.24115; found 499.23933.

2-[(4-Hydroxymethyl)benzyl]-5-(*tert*-butyldiphenylsililoximethyl)pyridazin-3(2*H*)-one (42). Compound **42** was purified by column chromatography on silica gel (50% EtOAc/hexane). Colourless oil; yield 71%; $R_f = 0.3$ (50% EtOAc/hexane); ^1H NMR ($CDCl_3$): $\delta = 7.68 - 7.63$ (m, 5H, H6, H-Ph), 7.48 – 7.43 (m, 2H, H-Ph), 7.42 – 7.36 (m, 6H, H-Ph), 7.33 – 7.29 (m, 2H, H-Ph), 6.95 (dd, 1H, $J = 3.5$, 1.5 Hz, H4), 5.29 (s, 2H, CH_2N), 4.65 (d, 2H, $J = 4.6$ Hz, $\mathit{CH_2}OH$), 4.55 (d, 2H, $J = 1.5$ Hz, CH_2OSi), 2.56 (br s, 1H, OH), 1.11 (s, 9H, $(CH_3)_3$); ^{13}C NMR ($CDCl_3$): $\delta = 160.6$ (C3), 145.7 (C5), 141.0 (C-Ph), 135.6 (C-Ph), 135.5 (CH-Ph), 135.5 (C6), 132.4 (C-Ph), 130.2 (CH-Ph), 129.0 (CH-Ph), 128.1 (CH-Ph), 127.2 (CH-Ph), 124.7 (C4), 64.9 (CH_2OH), 61.9 (CH_2OSi), 54.7 (CH_2N), 26.8 ($(CH_3)_3$), 19.3 ($\mathit{C}(CH_3)_3$); HRMS (ESI): *m/z* $[M+H]^+$ calcd for $C_{29}H_{33}N_2O_3Si$, 485.22550; found 485.22430.

2-[(4-Hydroxymethyl)benzyl]-4-(*tert*-butyldiphenylsililoximethyl)pyridazin-3(2*H*)-one (43). Compound **43** was purified by column chromatography on silica gel (30% EtOAc/hexane). Colourless oil; yield 56%; $R_f = 0.4$ (50% EtOAc/hexane); ^1H NMR ($CDCl_3$): $\delta = 7.84$ (d, 1H, $J = 4.0$ Hz, H6), 7.66 – 7.60 (m, 4H, H-Ph), 7.51 (dt, 1H, $J =$ 4.0, 1.8 Hz, H5), 7.45 – 7.28 (m, 10H, H-Ph), 5.28 (s, 2H, CH_2N), 4.72 (d, 2H, $J = 1.8$

Hz, CH_2OSi), 4.65 (d, 2H, $J = 4.4$ Hz, CH_2OH), 1.65 (br s, 1H, OH), 1.12 (s, 9H, $(CH_3)_3$); ^{13}C NMR ($CDCl_3$): $\delta = 159.4$ (C3), 143.4 (C4), 140.7 (C-Ph), 136.7 (C6), 135.8 (C-Ph), 135.6 (CH-Ph), 132.9 (C-Ph), 130.1 (CH-Ph), 129.2 (CH-Ph), 128.0 (CH-Ph), 127.3 (CH-Ph), 125.3 (C5), 65.2 (CH_2OH), 61.0 (CH_2OSi), 54.9 (CH_2N), 27.0 ($(CH_3)_3$), 19.5 ($C(CH_3)_3$); HRMS (ESI): *m/z* $[M+H]^+$ calcd for $C_{29}H_{33}N_2O_3Si$, 485.22550; found 485.22451.

General procedure to synthesize compounds 44-47. A solution of compound **40-43** (0.79 mmol) and TBAF (0.95 mmol, 1 M in THF) in THF (15 mL) was stirred at room temperature for 15 min. The solvent was evaporated to dryness and the residue was purified by column chromatography on silica gel to afford the proper compound.

6-Hydroxymethyl-2-[(4-hydroxymethyl)benzyl]pyridazin-3(2*H*)-one (44).
Compound **44** was purified by column chromatography on silica gel (5% MeOH/EtOAc). Yellowish oil; yield 87%; $R_f = 0.2$ (5% MeOH/EtOAc); ^{1}H NMR (CD_3OD): $\delta = 7.56$ (d, 1H, $J = 9.5$ Hz, H5), 7.39 – 7.30 (m, 4H, H-Ph), 7.01 (d, 1H, $J = 9.5$ Hz, H4), 5.31 (s, 2H, CH_2N), 4.59 (s, 2H, CH_2OH), 4.51 (s, 2H, CH_2OH-pyridazinone); ^{13}C NMR (CD_3OD): $\delta = 162.1$ (C3), 150.0 (C6), 142.5 (C-Ph), 136.7 (C-Ph), 133.5 (C5), 130.9 (C4), 129.4 (CH-Ph), 128.1 (CH-Ph), 64.8 (CH_2OH), 63.5 (CH_2OH-pyridazinone), 56.1 (CH_2N); HRMS (ESI): *m/z* $[M+H]^+$ calcd for $C_{13}H_{15}N_2O_3$, 247.10772; found 247.10715.

6-Hydroxymethyl-2-[(4-hydroxymethyl)benzyl]-5-methylpyridazin-3(2*H*)-one (45).
Compound **45** was purified by column chromatography on silica gel (5% MeOH/EtOAc). White solid; yield 97%; $R_f = 0.3$ (5% MeOH/EtOAc); m.p.: 142.3 – 142.7 °C; ^{1}H NMR (CD_3OD): $\delta = 7.38 - 7.29$ (m, 4H, H-Ph), 6.80 (d, 1H, $J = 1.2$ Hz, H4), 5.30 (s, 2H, CH_2N), 4.59 (s, 2H, CH_2OH), 4.57 (s, 2H, CH_2OH-pyridazinone), 2.35 (d, 3H, $J = 1.2$ Hz, CH_3); ^{13}C NMR (CD_3OD): $\delta = 162.4$ (C3), 149.2, 146.3, 142.4 (C-Ph), 136.8 (C-Ph), 129.4 (CH-Ph), 128.9 (C4), 128.1 (CH-Ph), 64.8 (CH_2OH), 62.9 (CH_2OH-pyridazinone), 55.7 (CH_2N), 17.7 (CH_3); HRMS (ESI): *m/z* $[M+H]^+$ calcd for $C_{14}H_{17}N_2O_3$, 261.12337; found 261.12260.

5-Hydroxymethyl-2-[(4-hydroxymethyl)benzyl]pyridazin-3(2*H*)-one (46).

Compound **46** was purified by column chromatography on silica gel (EtOAc). White solid; yield 96%; R_f = 0.2 (EtOAc); m.p. = 143.2 – 144.0 °C; ^{1}H NMR (CD_3OD): δ = 7.91 (d, 1H, J = 2.1 Hz, H6), 7.38 – 7.30 (m, 4H, H-Ph), 6.93 (dd, 1H, J = 3.4, 1.5 Hz, H4), 5.33 (s, 2H, CH_2N), 4.59 (s, 2H, CH_2OH), 4.55 (d, 2H, J = 1.5 Hz, CH_2OH-pyridazinone); ^{13}C NMR (CD_3OD): δ = 162.6 (C3), 149.6 (C5), 142.5 (C-Ph), 138.2 (C6), 136.7 (C-Ph), 129.4 (CH-Ph), 128.1 (CH-Ph), 125.0 (C4), 64.8 (CH_2OH), 61.0 (CH_2OH-pyridazinone), 55.9 (CH_2N); HRMS (ESI): *m/z* $[M+Na]^+$ calcd for $C_{13}H_{14}N_2NaO_3$, 269.08966; found 269.08950.

4-Hydroxymethyl-2-((4-hydroxymethyl)benzyl)pyridazin-3(2*H*)-one (47).
Compound **47** was purified by column chromatography on silica gel (EtOAc). White solid; yield 95%; R_f = 0.4 (5% MeOH/EtOAc); m.p: 137.4 – 138.0 °C; ^{1}H NMR (CD_3OD): δ = 7.97 (d, 1H, J = 4.0 Hz, H6), 7.48 (dt, 1H, J = 4.0, 1.7 Hz, H5), 7.39 – 7.30 (m, 4H, H-Ph), 5.36 (s, 2H, CH_2N), 4.60 (s, 2H, CH_2OH), 4.56 (d, 2H, J = 1.7 Hz, CH_2OH-pyridazinone); ^{13}C NMR (CD_3OD): δ = 161.4 (C3), 144.9 (C4), 142.5 (C-Ph), 138.7 (C6), 136.7 (C-Ph), 129.4 (CH-Ph), 128.1 (CH-Ph), 127.4 (C5), 64.8 (CH_2OH), 59.5 (CH_2OH-pyridazinone), 55.9 (CH_2N); HRMS (ESI): *m/z* $[M+Na]^+$ calcd for $C_{13}H_{14}N_2NaO_3$, 269.08966; found 269.08953.

General procedure to synthesize compounds 48-51. To a solution of compound **44-47** (0.10 mmol) in CH_2Cl_2 (5 mL) was added CBr_4 (0.60 mmol) and PPh_3 (0.60 mmol) and the reaction mixture was refluxed for 3 h (**48**, **49** and **51**) or 6 h (**50**). After quenching with saturated aq. $NaHCO_3$ (5 mL) the product was extracted with CH_2Cl_2 (3x5 mL), dried over Na_2SO_4 and the solvent was removed under reduced pressure. The residue was purified by column chromatography on silica gel (30% EtOAc/hexane) to afford the desired compound.

6-Bromomethyl-2-[(4-bromomethyl)benzyl]pyridazin-3(2*H*)-one (48). White solid; yield 65%; R_f = 0.5 (EtOAc); m.p.= 92.5 – 93.0 °C; ^{1}H NMR ($CDCl_3$): δ = 7.41 – 7.34 (m, 4H, H-Ph), 7.32 (d, 1H, J = 9.6 Hz, H5), 6.94 (d, 1H, J = 9.6 Hz, H4), 5.26 (s, 2H, CH_2N), 4.46 (s, 2H, CH_2Br), 4.33 (s, 2H, CH_2Br-pyridazinone); ^{13}C NMR ($CDCl_3$): δ = 159.4 (C3), 143.7 (C6), 137.7 (C-Ph), 136.3 (C-Ph), 132.4 (C5), 131.0 (C4), 129.5 (CH-Ph), 129.4 (CH-Ph), 55.1 (CH_2N), 33.2 (CH_2Br), 30.4 (CH_2Br-pyridazinone); HRMS (ESI): *m/z* $[M+H]^+$ calcd for $C_{13}H_{13}Br_2N_2O$, 370.93891; found 370.93806.

6-Bromomethyl-2-[(4-bromomethyl)benzyl]-5-methylpyridazin-3(2*H*)-one (49). Colourless oil; yield 67%; R_f = 0.6 (EtOAc); ^{1}H NMR ($CDCl_3$): δ = 7.36 (m, 4H, H-Ph), 6.72 (d, 1H, *J* = 1.1 Hz, H4), 5.25 (s, 2H, CH_2N), 4.45 (s, 2H, CH_2Br), 4.36 (s, 2H, CH_2Br-pyridazinone), 2.30 (d, 3H, *J* = 1.1 Hz, CH_3); ^{13}C NMR ($CDCl_3$): δ = 160.0 (C3), 143.6, 142.8, 137.6 (C-Ph), 136.4 (C-Ph), 129.4 (CH-Ph), 129.3, 129.2, 54.7 (CH_2N), 33.2 (CH_2Br), 29.3 (CH_2Br-pyridazinone), 18.1 (CH_3); HRMS (ESI): *m/z* $[M+H]^+$ calcd for $C_{14}H_{15}Br_2N_2O$, 384.95456; found 384.95333.

5-Bromomethyl-2-[(4-bromomethyl)benzyl]pyridazin-3(2*H*)-one (50). White solid; yield 90%; R_f = 0.4 (50% EtOAc/hexane); m.p. = 145.5 – 146.3 °C, ^{1}H NMR ($CDCl_3$): δ = 7.78 (d, 1H, *J* = 2.2 Hz, H6), 7.43 - 7.39 (m, 2H, H-Ph), 7.37 - 7.34 (m, 2H, H-Ph), 6.86 (dd, 1H, *J* = 2.2, 0.6 Hz, H4), 5.29 (s, 2H, CH_2N), 4.46 (s, 2H, CH_2Br), 4.18 (d, 2H, *J* = 0.6 Hz, CH_2Br-pyridazinone); ^{13}C NMR ($CDCl_3$): δ = 159.7 (C3), 141.9 (C5), 137.7 (C-Ph), 137.1 (C6), 136.3 (C-Ph), 129.5 (CH-Ph), 129.4 (CH-Ph), 128.0 (C4), 54.8 (CH_2N), 33.2 (CH_2Br), 26.9 (CH_2Br-pyridazinone); HRMS (ESI): *m/z* $[M+H]^+$ calcd for $C_{13}H_{13}Br_2N_2O$, 370.93891; found 370.93891.

4-Bromomethyl-2-[(4-bromomethyl)benzyl]pyridazin-3(2*H*)-one (51). White solid; yield 98%; R_f = 0.5 (50% EtOAc/hexane); m.p. = 124.3 – 124.6 °C, ^{1}H NMR ($CDCl_3$): δ = 7.77 (d, 1H, *J* = 4.1 Hz, H6), 7.42 (d, 2H, *J* = 8.2 Hz, H-Ph), 7.35 (d, 2H, *J* = 8.2 Hz, H-Ph), 7.32 (dt, 1H, *J* = 4.1, 0.8 Hz, H5), 5.33 (s, 2H, CH_2N), 4.46 (s, 2H, CH_2Br), 4.39 (d, 2H, *J* = 0.8 Hz, CH_2Br-pyridazinone); ^{13}C NMR ($CDCl_3$): δ = 159.5 (C3), 139.1 (C4), 137.8 (C-Ph), 136.2 (C-Ph), 136.2 (C6), 129.8 (C5), 129.5 (2xCH-Ph), 55.4 (CH_2N), 33.2 (CH_2Br), 26.5 (CH_2Br-pyridazinone); HRMS (ESI): *m/z* $[M+H]^+$ calcd for $C_{13}H_{13}Br_2N_2O$, 370.93891; found 370.93875.

General procedure to synthesize compounds 52-55. To a solution of compound **48-51** (0.05 mmol) in DMF (2 mL) was added 1,3-bis-(*tert*-butoxycarbonyl)guanidine (0.28 mmol) and K_2CO_3 (0.11 mmol) and the reaction was stirred at 50 °C for 2 h. The reaction mixture was diluted with EtOAc (40 mL), washed with water (10 mL), brine (2x10 mL) and dried over anhydrous $MgSO_4$. The solvent was evaporated to dryness and the residue was purified by column chromatography on silica gel (50% EtOAc/hexane for **52-54** and 20% EtOAc/hexane for **55**) to afford the desired compound.

2-[4-((*N,N'*-di(*tert*-Butoxycarbonyl)guanidine)methyl)benzyl]-6-[(*N,N'*-di(*tert*-butoxycarbonyl)guanidine]methylpyridazin-3(2*H*)-one (52). White solid; yield 60%; R_f = 0.3 (50% EtOAc/hexane); m.p.= 175.7 – 176.2 °C; ^{1}H NMR ($CDCl_3$): δ = 9.54 – 9.18 (m, 4H, 4xNH), 7.29 (d, 2H, J = 8.2 Hz, H-Ph), 7.23 – 7.15 (m, 3H, H5, H-Ph), 6.89 (d, 1H, J = 9.5 Hz, H4), 5.24 (s, 2H, CH_2N), 5.13 (s, 2H, CH_2NH), 5.06 (s, 2H, NHCH_2-pyridazinone), 1.47 (s, 18H, 2x(CH_3)$_3$), 1.34 (s, 9H, (CH_3)$_3$), 1.28 (s, 9H, (CH_3)$_3$); ^{13}C NMR ($CDCl_3$): δ =163.9 (CO), 163.6 (CO), 161.0 (CO), 160.5 (CO), 160.1 (C3), 155.1 (C=N), 154.6 (C=N), 144.7 (C6), 138.6 (C-Ph), 135.1 (C-Ph), 131.2 (C5), 130.4 (C4), 128.6 (CH-Ph), 127.5 (CH-Ph), 84.8 (C(CH_3)$_3$), 84.3 (C(CH_3)$_3$), 79.3 (C(CH_3)$_3$), 78.9 (C(CH_3)$_3$), 55.0 (CH_2N), 47.3 (CH_2NH), 46.6 (NHCH_2-pyridazinone), 28.5 ((CH_3)$_3$), 28.4 ((CH_3)$_3$), 28.0 ((CH_3)$_3$), 27.9 ((CH_3)$_3$); HRMS (ESI): *m/z* [M+H]$^+$ calcd for $C_{35}H_{53}N_8O_9$, 729.39300; found 729.39207.

2-[4-((*N,N'*-di(*tert*-Butoxycarbonyl)guanidine)methyl)benzyl]-6-[(*N,N'*-di(*tert*-butoxycarbonyl)guanidine]methyl-5-methylpyridazin-3(2*H*)-one (53). Colourless oil; yield 60%; R_f = 0.2 (50% EtOAc/hexane); ^{1}H NMR ($CDCl_3$): δ = 9.53 – 9.19 (m, 4H, 4xNH), 7.26 (d, 2H, J = 8.2 Hz, H-Ph), 7.14 (d, 2H, J = 8.2 Hz, H-Ph), 6.66 (d, 1H, J = 1.2 Hz, H4), 5.17 (s, 2H, CH_2N), 5.11 (s, 2H, CH_2NH), 5.03 (s, 2H, NHCH_2-pyridazinone), 2.17 (d, 3H, J = 1.2 Hz, CH_3), 1.46 (s, 9H, (CH_3)$_3$), 1.45 (s, 9H, (CH_3)$_3$), 1.32 (s, 9H, (CH_3)$_3$), 1.25 (s, 9H, (CH_3)$_3$); ^{13}C NMR ($CDCl_3$): δ = 163.9 (CO), 163.6 (CO), 160.9 (CO), 160.6 (CO), 160.2 (C3), 155.1 (C=N), 154.6 (C=N), 143.7, 141.7, 138.5 (C-Ph), 135.0 (C-Ph), 128.9 (CH-Ph), 128.4 (C4), 127.3 (CH-Ph), 84.2 (C(CH_3)$_3$), 84.2 (C(CH_3)$_3$), 79.0 (C(CH_3)$_3$), 78.9 (C(CH_3)$_3$), 54.5 (CH_2N), 47.3 (CH_2NH), 44.2 (NHCH_2-pyridazinone), 28.4 ((CH_3)$_3$), 28.4 ((CH_3)$_3$), 28.0 ((CH_3)$_3$), 27.8 ((CH_3)$_3$), 17.7 (CH_3); HRMS (ESI): *m/z* [M+H]$^+$ calcd for $C_{36}H_{55}N_8O_9$, 743.40865; found 743.40521.

2-[4-((*N,N'*-di(*tert*-Butoxycarbonyl)guanidine)methyl)benzyl]-5-[(*N,N'*-di(*tert*-butoxycarbonyl)guanidine]methylpyridazin-3(2*H*)-one (54). White solid; yield 61%; R_f = 0.4 (50% EtOAc/hexane); m.p. = 166.9 –167.5 °C; ^{1}H NMR ($CDCl_3$): δ =9.64 - 8.94 (m, 4H, 4xNH), 7.75 (d, 2H, J = 2.2 Hz, H6), 7.31 (d, 2H, J = 8.2 Hz, H-Ph), 7.20 (d, 2H, J = 8.2 Hz, H-Ph), 6.74 - 6.71 (m, 1H, H4), 5.29 (s, 2H, CH_2N), 5.14 (s, 2H, CH_2NH), 4.99 (s, 2H, NHCH_2-pyridazinone), 1.47 (s, 9H, (CH_3)$_3$), 1.46 (s, 9H, (CH_3)$_3$), 1.41 (s,

9H, $(CH_3)_3$), 1.32 (s, 9H, $(CH_3)_3$); ^{13}C NMR ($CDCl_3$): δ =163.9 (CO), 163.4 (CO), 160.9 (CO), 160.3 (CO), 160.0 (C3), 155.1 (C=N), 154.2 (C=N), 143.5 (C5), 138.7 (C-Ph), 136.7 (C6), 135.1 (C-Ph), 128.5 (CH-Ph), 127.5 (CH-Ph), 126.3 (C4), 85.5 (*C*$(CH_3)_3$), 84.2 (*C*$(CH_3)_3$), 79.4 (*C*$(CH_3)_3$), 79.0 (*C*$(CH_3)_3$), 54.6 (CH_2N), 47.4 (CH_2NH), 44.5 ($NHCH_2$-pyridazinone), 28.4 ($(CH_3)_3$), 28.4 ($(CH_3)_3$), 28.1 ($(CH_3)_3$), 28.0 ($(CH_3)_3$); HRMS (ESI): *m/z* $[M+H]^+$ calcd for $C_{35}H_{53}N_8O_9$, 729.39172; found 729.39300.

2-[4-((*N,N'*-di(*tert*-Butoxycarbonyl)guanidine)methyl)benzyl]-4-[(*N,N'*-di(*tert*-butoxycarbonyl)guanidine]methylpyridazin-3(2*H*)-one (55). Colourless oil; yield 70%; R_f = 0.6 (50% EtOAc/hexane); ^1H NMR ($CDCl_3$): δ =9.55 – 9.16 (m, 4H, 4xNH), 7.71 (d, 1H, J = 4.1 Hz, H6), 7.33 (d, 2H, J = 8.2 Hz, H-Ph), 7.20 (d, 2H, J = 8.2 Hz, H-Ph), 6.85 (dt, 1H, J = 4.1, 1.3 Hz, H5), 5.31 (s, 2H, CH_2N), 5.17 – 5.11 (m, 4H, NHCH_2-pyridazinone, CH_2NH), 1.47 (s, 9H, $(CH_3)_3$), 1.44 (s, 9H, $(CH_3)_3$), 1.33 (s, 9H, $(CH_3)_3$), 1.29 (s, 9H, $(CH_3)_3$); ^{13}C NMR ($CDCl_3$): δ = 163.9 (CO), 163.8 (CO), 161.0 (CO), 160.4 (CO), 159.8 (C3), 155.1 (C=N), 154.5 (C=N), 141.1 (C4), 138.7 (C-Ph), 136.1 (C6), 135.1 (C-Ph), 128.7 (CH-Ph), 127.5 (CH-Ph), 124.9 (C5), 84.8 (*C*$(CH_3)_3$), 84.3 (*C*$(CH_3)_3$), 79.3 (*C*$(CH_3)_3$), 79.0 (*C*$(CH_3)_3$), 55.0 (CH_2N), 47.4 (CH_2NH), 43.2 ($NHCH_2$-pyridazinone), 28.5 ($(CH_3)_3$), 28.4 ($(CH_3)_3$), 28.0 ($(CH_3)_3$), 27.9 ($(CH_3)_3$); HRMS (ESI): *m/z* $[M+H]^+$ calcd for $C_{35}H_{53}N_8O_9$, 729.39300; found 729.39231.

2-Benzyl-6-(hydroximethyl)-5-methylpyridazin-3(2*H*)-one (58). Following a similar procedure as that used for the synthesis of **44-46**, compound **58** was obtained as a white solid (145 mg, 83%) from **29** (370 mg. 0.79 mmol) and TBAF (0.95 mmol, 1M in THF) in THF (15 mL) after purification by column chromatography on silica gel (EtOAc). R_f = 0.4 (EtOAc); m.p. = 102.7 – 103.2 °C; ^1H NMR ($CDCl_3$): δ = 7.38 - 7.35 (m, 2H, H-Ph), 7.32 – 7.24 (m, 3H, H-Ph), 6.69 (s, 1H, H4), 5.26 (s, 2H, CH_2N), 4.54 (s, 2H, CH_2O), 3.55 (br s, 1H, OH), 2.17 (s, 3H, CH_3); ^{13}C NMR ($CDCl_3$): δ = 160.4 (C3), 145.4, 142.0, 136.2 (C-Ph), 128.9, 128.7, 128.0 (CH-Ph), 61.6 (CH_2O), 54.7 (CH_2N), 17.0 (CH_3); HRMS (ESI): *m/z* $[M+H]^+$ calcd for $C_{13}H_{15}N_2O_2$, 231.11280; found 231.11237.

2-Benzyl-6-bromomethyl-5-methylpyridazin-3(2*H*)-one (65). Following a similar procedure as that used for the synthesis of **48-51**, compound **65** was obtained as a white solid (117 mg, 67%) from **58** (138 mg, 0.60 mmol) CBr_4 (396 mg, 1.20 mmol) and Ph_3P

(316 mg, 1.20 mmol) in CH_2Cl_2 (10 mL) after purification by column chromatography on silica gel (40% EtOAc/hexane). $R_f = 0.5$ (50% EtOAc/hexane); m.p. = 99.8 – 100.2 ^oC; ^1H NMR ($CDCl_3$): $\delta = 7.44$ - 7.35 (m, 2H, H-Ph), 7.36 - 7.22 (m, 3H, H-Ph), 6.72 (d, 1H, $J = 1.1$ Hz, H4), 5.27 (s, 2H, CH_2N), 4.36 (s, 2H, CH_2Br), 2.29 (d, 3H, $J = 1.1$ Hz, CH_3); ^{13}C NMR ($CDCl_3$): $\delta = 160.0$ (C3), 143.4, 142.7, 136.1 (C-Ph), 129.0 (CH-Ph), 128.7, 128.6, 128.0 (CH-Ph), 55.0 (CH_2N), 29.3 (CH_2Br), 18.0 (CH_3); HRMS (ESI): *m/z* $[M+H]^+$ calcd for $C_{13}H_{14}BrN_2O$, 293.02840; found 293.02851.

General procedure to synthesize compounds 70-71. A solution of compound **25** or **31** (0.11 mmol) in THF (3 mL) was added, dropwise at 0 ^oC, to a suspension of NaH (0.17 mmol, 60% dispersion in mineral oil) in THF (2 mL). After the mixture was stirred at room temperature for 1 h, α,α'-dibromo-*p*-xylene (0.12 mmol) and Bu_4NI (0.01 mmol) were added. The reaction mixture was stirred at room temperature for 5 h, followed by quenching with MeOH. The solvent was evaporated to dryness, and the residue was purified by column chromatography on silica gel (20% EtOAc/hexane for **70** and 10% EtOAc/hexane for **71**) to afford the desired compound.

2-((4-bromomethyl)benzyl)-6-(*tert*-butyldiphenylsililoximethyl)-5-methylpyridazin-3(2*H*)-one (70). Colourless oil; yield 52%; $R_f = 0.5$ (50% EtOAc/hexane); ^1H NMR ($CDCl_3$): $\delta = 7.69$ - 7.63 (m, 4H, H-Ph), 7.49 - 7.41 (m, 2H, H-Ph), 7.41 - 7.29 (m, 8H, H-Ph), 6.72 (d, 1H, $J = 1.1$ Hz, H4), 5.18 (s, 2H, CH_2N), 4.65 (s, 2H, CH_2O), 4.45 (s, 2H, CH_2Br), 2.33 (d, 3H, $J = 1.1$ Hz, CH_3), 1.07 (s, 9H, $(CH_3)_3$); ^{13}C NMR ($CDCl_3$): $\delta = 160.2$ (C3), 146.0, 143.5, 137.2 (C-Ph), 136.8 (C-Ph), 135.6 (CH-Ph), 132.9 (C-Ph), 129.9 (CH-Ph), 129.2 (CH-Ph), 129.1 (CH-Ph), 128.6 (C4), 127.8 (CH-Ph), 64.5 (CH_2O), 54.2 (CH_2N), 33.2 (CH_2Br), 26.8 ($(CH_3)_3$), 19.3 ($C(CH_3)_3$), 17.9 (CH_3); HRMS (ESI): *m/z* $[M+H]^+$ calcd for $C_{30}H_{34}BrN_2O_2Si$, 561.15674; found 561.15645.

2-((4-bromomethyl)benzyl)-4-(*tert*-butyldiphenylsililoximethyl)pyridazin-3(2*H*)-one (71). Colourless oil; yield 50%; $R_f = 0.3$ (10% EtOAc/hexane); ^1H NMR ($CDCl_3$): $\delta = 7.85$ (d, 1H, $J = 4.0$ Hz, H6), 7.66 - 7.61 (m, 4H, H-Ph), 7.53 - 7.49 (m, 1H, H5), 7.45 - 7.31 (m, 10H, H-Ph), 5.28 (s, 2H, CH_2N), 4.73 (d, 2H, $J = 1.8$ Hz, CH_2O), 4.45 (s, 2H, CH_2Br), 1.12 (s, 9H, $(CH_3)_3$); ^{13}C NMR ($CDCl_3$): $\delta = 159.4$ (C3), 143.5 (C4), 137.6 (C-Ph), 136.8 (C-Ph), 136.6 (C6), 135.6 (CH-Ph), 132.8 (C-Ph), 130.1 (CH-Ph), 129.4 (CH-Ph), 129.4 (CH-Ph), 128.0 (CH-Ph), 125.4 (C5), 60.9 (CH_2O), 54.8 (CH_2N), 33.2

(CH_2Br), 27.0 (($CH_3)_3$), 19.5 ($C(CH_3)_3$); HRMS (ESI): *m/z* $[M+H]^+$ calcd for $C_{29}H_{32}BrN_2O_2Si$, 547.14109; found 547.14111.

2-((4-Fluoromethyl)benzyl)-6-hydroxymethyl-5-methylpyridazin-3(2*H*)-one (72). Following a similar procedure as that used for the synthesis of **44-46**, compound **72** was obtained as a white solid (141 mg, 68%) from **70** (207 mg. 0.79 mmol) and TBAF (0.95 mmol, 1 M in THF) in THF (15 mL) after purification by column chromatography on silica gel (70% EtOAc/hexane). R_f = 0.3 (EtOAc); m.p.: 118.8 – 120.0 °C; ^{1}H NMR ($CDCl_3$): δ = 7.40 – 7.34 (m, 2H, H-Ph), 7.31 – 7.22 (m, 2H, H-Ph), 6.67 (d, 1H, J = 1.2 Hz, H4), 5.30 (d, 2H, J_{HF} = 47.7 Hz, CH_2F), 5.25 – 5.21 (m, 2H, CH_2N), 4.53 (s, 2H, CH_2O), 3.46 (br s, 1H, OH), 2.16 (d, 3H, J = 1.2 Hz, CH_3); ^{13}C NMR ($CDCl_3$): δ = 160.3 (C3), 146.2, 142.5, 136.8 (C-Ph, $^5J_{CF}$ = 3.4 Hz), 135.9 (C-Ph, $^2J_{CF}$ = 15.5 Hz), 128.8 (CH-Ph), 128.7 (C4), 127.7 (CH-Ph, $^3J_{CF}$ = 5.9 Hz), 84.2 (CH_2F, $^1J_{CF}$ = 166.1 Hz), 61.7 (CH_2O), 54.3 (CH_2N), 17.1 (CH_3); ^{19}F NMR ($CDCl_3$): δ = -207.1 (CH_2F); HRMS (ESI): *m/z* $[M+H]^+$ calcd for $C_{14}H_{16}FN_2O_2$, 263.11903; found 263.11913.

4-hydroxymethyl-2-(4-methoxymethyl)benzylpyridazin-3(2*H*)-one (73). A mixture of compound **71** (33 mg, 0.06 mmol) and TMSBr (18 mg, 0.12 mmol) in MeOH (2 mL) was stirred at reflux for 9 h followed by quenching with saturated aq. $NaHCO_3$ (0.5 mL) and H_2O (5 mL). The mixture was extracted with EtOAc (3x5 mL) and the combined organic phases were dried over Na_2SO_4, filtered and the solvent was evaporated to dryness. The residue was purified by column chromatography on silica gel (50% EtOAc/hexane) to afford compound **73** (12.1 mg, 75%) as a white solid. R_f = 0.3 (50% EtOAc/hexane); m.p.= 84.3 – 84.9 °C; ^{1}H NMR ($CDCl_3$): δ = 7.79 (d, 1H, J = 4.0 Hz, H6), 7.40 (d, 2H, J = 8.1 Hz, H-Ph), 7.29 (d, 2H, J = 8.1 Hz, H-Ph), 7.19 (dt, 1H, J = 4.0, 1.3 Hz, H5), 5.32 (s, 2H, CH_2N), 4.62 (s, 2H, $\mathit{CH_2}OH$), 4.42 (s, 2H, $\mathit{CH_2}OCH_3$), 3.36 (s, 3H, CH_3), 3.13 (br s, 1H, OH); ^{13}C NMR ($CDCl_3$): δ = 160.5 (C3), 142.0 (C4), 138.2 (C-Ph), 136.7 (C6), 135.5 (C-Ph), 129.0 (CH-Ph), 128.1 (CH-Ph), 126.4 (C5), 74.5 ($\mathit{C}H_2OCH_3$), 61.0 (CH_2OH), 58.3 (CH_3), 55.1 (CH_2N); HRMS (ESI): m/z $[M+H]^+$ calcd for $C_{14}H_{17}N_2O_3$, 261.12337; found 261.12284.

6-Bromomethyl-2-((4-fluoromethyl)benzyl)-5-methylpyridazin-3(2*H*)-one (74).

Following a similar procedure as that used for the synthesis of **48-51**, compound **74** (23 mg, 70%) was obtained as a colourless oil from **72** (26 mg, 0.10 mmol) CBr_4 (199 mg, 0.60 mmol) and PPh_3 (158 mg, 0.60 mmol) in CH_2Cl_2 (5 mL) after purification by column chromatography on silica gel (30% EtOAc/hexane). R_f = 0.5 (EtOAc); ^{1}H NMR ($CDCl_3$): δ = 7.45 – 7.37 (m, 2H, H-Ph), 7.36 – 7.29 (m, 2H, H-Ph), 6.71 (d, 1H, J = 1.1 Hz, H4), 5.33 (d, 2H, J_{HF} = 47.7 Hz, CH_2F), 5.27 (s, 2H, CH_2N), 4.35 (s, 2H, CH_2Br), 2.29 (d, 3H, J = 1.1 Hz, CH_3); ^{13}C NMR ($CDCl_3$): δ = 160.0 (C3), 143.6, 142.7, 136.7 (C-Ph, $^5J_{CF}$ = 3.0 Hz), 136.0 (C-Ph, $^2J_{CF}$ = 16.9 Hz), 129.1, 129.0, 127.8 (CH-Ph, $^3J_{CF}$ = 6.1 Hz), 84.3 (CH_2F, $^1J_{CF}$ = 166.3 Hz), 54.7 (CH_2N), 29.3 (CH_2Br), 18.0 (CH_3); ^{19}F NMR ($CDCl_3$): δ = -207.3 (CH_2F); HRMS (ESI): *m/z* $[M+H]^+$ calcd for $C_{14}H_{15}BrFN_2O$, 325.02743; found 325.02685.

4-Bromomethyl-2-(4-methoxylmethyl)benzylpyridazin-3(2*H*)-one (75). Following a similar procedure as that used for the synthesis of **48-51**, compound **75** (30 mg, 94%) was obtained as a colourless oil from **73** (26 mg, 0.10 mmol) CBr_4 (199 mg, 0.60 mmol) and PPh_3 (158 mg, 0.60 mmol) in CH_2Cl_2 (5 mL) after purification by column chromatography on silica gel (30% EtOAc/hexane)Colourless oil; yield 94%; R_f = 0.5 (50% EtOAc/hexane); ^{1}H NMR ($CDCl_3$): δ = 7.76 (d, 1H, J = 4.1 Hz, H6), 7.42 (d, 2H, J = 8.1 Hz, H-Ph), 7.32 – 7.27 (m, 3H, H5, H-Ph), 5.34 (s, 2H, CH_2N), 4.42 (s, 2H, CH_2O), 4.39 (d, 2H, J = 0.7 Hz, CH_2Br), 3.36 (s, 3H, CH_3); ^{13}C NMR ($CDCl_3$): δ = 159.5 (C3), 139.0 (C4), 138.3 (C-Ph), 136.0 (C6), 135.4 (C-Ph), 129.7 (C5), 129.1 (CH-Ph), 128.1 (CH-Ph), 74.5 (CH_2O), 58.3 (CH_3), 55.5 (CH_2N), 26.5 (CH_2Br); HRMS (ESI): *m/z* $[M+H]^+$ calcd for $C_{14}H_{16}BrN_2O_2$, 323.03897; found 323.03847.

General procedure to synthesize compounds 76-78 and 80-83. To a solution of compound **63-69** (0.25 mmol) in DMF (1.5 mL) was added 1,3-bis(*tert*-butoxycarbonyl)guanidine (0.28 mmol) and K_2CO_3 (0.38 mmol) and the reaction was stirred at 50 °C for 2 h. The reaction mixture was diluted with EtOAc (40 mL), washed with water (10 mL), brine (2x10 mL) and dried over anhydrous $MgSO_4$. The solvent was evaporated to dryness and the residue was purified by column chromatography on silica gel (50% EtOAc/hexane for **76-78** and **80-82** and 20% EtOAc/hexane for **83**) to afford the desired compound.

2-Methyl-6-[(*N,N'*-di(*tert*-butoxycarbonyl)guanidine]methylpyridazin-3(2*H*)-one (76). White solid; yield 57%; R_f = 0.4 (EtOAc); m.p. = 162.0 – 164.0 °C; ^{1}H NMR ($CDCl_3$): δ = 9.50 - 9.10 (m, 2H, 2xNH), 7.21 (d, 1H, *J* = 9.5 Hz, H5), 6.85 (d, 1H, *J* = 9.5 Hz, H4), 5.04 (s, 2H, CH_2), 3.69 (s, 3H, CH_3N), 1.43 (s, 9H, $(CH_3)_3$), 1.36 (s, 9H, $(CH_3)_3$); ^{13}C NMR ($CDCl_3$): δ = 163.3 (CO), 160.3, 160.2, 154.4 (C=N), 144.1 (C6), 131.2 (C5), 129.5 (C4), 84.5 (*C*$(CH_3)_3$), 79.1 (*C*$(CH_3)_3$), 46.3 (CH_2), 40.1 (CH_3N), 28.2 ($(CH_3)_3$), 27.8 ($(CH_3)_3$); HRMS (ESI): *m/z* $[M+H]^+$ calcd for $C_{17}H_{28}N_5O_5$, 382.2090; found 382.2072.

2,5-di-Methyl-6-[(*N,N'*-di(*tert*-butoxycarbonyl)guanidine]methylpyridazin-3(2*H*)-one (77). White solid; yield 55%; R_f = 0.6 (EtOAc); m.p. = 148.0 – 150.0 °C; ^{1}H NMR ($CDCl_3$): δ = 9.51 - 9.23 (m, 2H, 2xNH), 6.66 (s, 1H, H4), 5.06 (s, 2H, CH_2), 3.66 (s, 3H, CH_3N), 2.19 (s, 3H, CH_3), 1.44 (s, 9H, $(CH_3)_3$), 1.36 (s, 9H, $(CH_3)_3$); ^{13}C NMR ($CDCl_3$): δ = 163.5 (CO), 160.6, 160.4, 154.6 (C=N), 143.3, 141.7, 127.7 (C4), 84.0 (*C*$(CH_3)_3$), 78.9 (*C*$(CH_3)_3$), 44.1 (CH_2), 39.8 (CH_3N), 28.2 ($(CH_3)_3$), 27.7 ($(CH_3)_3$), 17.6 (CH_3); HRMS (ESI): *m/z* $[M+Na]^+$ calcd for $C_{18}H_{29}N_5O_5Na$, 418.2066; found 418.2065.

2-Benzyl-5-methyl-6-[(*N,N'*-di(*tert*-butoxycarbonyl)guanidine]methylpyridazin-3(2*H*)-one (78). Yellow oil; yield 59%; R_f = 0.5 (EtOAc); ^{1}H NMR ($CDCl_3$): δ = 9.57 - 9.20 (m, 2H, 2xNH), 7.35 - 7.30 (m, 2H, H-Ph), 7.27 - 7.18 (m, 3H, H-Ph), 6.66 (s, 1H, H4), 5.18 (s, 2H, CH_2N), 5.03 (s, 2H, *CH_2*NH), 2.16 (s, 3H, CH_3), 1.44 (s, 9H, $(CH_3)_3$), 1.25 (s, 9H, $(CH_3)_3$); ^{13}C NMR ($CDCl_3$): δ = 163.3 (CO), 160.5 (CO), 160.0 (C3), 154.4 (C=N), 143.4, 141.4, 136.2 (C-Ph), 129.0 (CH-Ph), 128.4, 128.3, 127.7 (CH-Ph), 84.0 (*C*$(CH_3)_3$), 78.9 (*C*$(CH_3)_3$), 54.4 (CH_2N), 44.1 (CH_2NH), 28.2 ($(CH_3)_3$), 27.7 ($(CH_3)_3$), 17.6 (CH_3); HRMS (ESI): *m/z* $[M+Na]^+$ calcd for $C_{24}H_{33}N_5O_5Na$, 494.2379; found 494.2380.

2-Methyl-5-[(*N,N'*-di(*tert*-butoxycarbonyl)guanidine]methylpyridazin-3(2*H*)-one (80). White solid; yield 94%; R_f = 0.3 (50% EtOAc/hexane); m.p. = 143.0 – 145.0 °C; ^{1}H NMR ($CDCl_3$): δ =9.47 – 9.01 (m, 2H, 2xNH), 7.68 (d, 1H, *J* = 2.2 Hz, H6), 6.67 – 6.63 (m, 1H, H4), 4.95 (s, 2H, CH_2), 3.70 (s, 3H, CH_3N), 1.41 (s, 9H, $(CH_3)_3$), 1.38 (s, 9H, $(CH_3)_3$); ^{13}C NMR ($CDCl_3$): δ = 163.1 (CO), 160.6 (C3), 159.8 (CO), 154.0 (C=N), 143.4 (C5), 136.1 (C6), 125.4 (C4), 85.3 (*C*$(CH_3)_3$), 79.3 (*C*$(CH_3)_3$), 44.3 (CH_2), 39.9 (CH_3N),

28.2 (($(CH_3)_3$), 27.9 (($(CH_3)_3$); HRMS (ESI): *m/z* $[M+H]^+$ calcd for $C_{17}H_{28}N_5O_5$, 382.2090; found 382.2088.

2-Methyl-4-[(*N,N'*-di(*tert*-butoxycarbonyl)guanidine]methylpyridazin-3(2*H*)-one (81). White solid; yield 84%; R_f = 0.5 (50% EtOAc/hexane); m.p. = 115.0 – 118.0 °C; ^{1}H NMR ($CDCl_3$): δ = 9.54 - 9.06 (m, 2H, 2xNH), 7.67 (d, 1H, *J* = 4.1 Hz, H6), 6.85 (d, 1H, *J* = 4.1 Hz, H5), 5.13 (s, 2H, CH_2), 3.79 (s, 3H, CH_3N), 1.42 (s, 9H, $(CH_3)_3$), 1.36 (s, 9H, $(CH_3)_3$); ^{13}C NMR ($CDCl_3$): δ = 163.6 (CO), 160.2 (CO), 160.0 (C3), 154.5 (C=N), 140.1 (C4), 136.6 (C6), 124.8 (C5), 84.6 (*C*$(CH_3)_3$), 79.1 (*C*$(CH_3)_3$), 43.2 (CH_2), 40.0 (CH_3N), 28.2 ($(CH_3)_3$), 27.8 ($(CH_3)_3$); HRMS (ESI): *m/z* $[M+H]^+$ calcd for $C_{17}H_{28}N_5O_5$, 382.2090; found 382.2100.

2-Benzyl-5-[(*N,N'*-di(*tert*-butoxycarbonyl)guanidine]methylpyridazin-3(2*H*)-one (82). White solid; yield 90%; R_f = 0.5 (50% EtOAc/hexane); m.p. = 145.0 – 148.0 °C; ^{1}H NMR ($CDCl_3$): δ = 9.56 – 9.11 (m, 2H, 2xNH), 7.73 (d, 1H, *J* = 2.1 Hz, H6), 7.39 - 7.34 (m, 2H, H-Ph), 7.32 - 7.21 (m, 3H, H-Ph) 6.76 – 6.73 (m, 1H, H4), 5.29 (s, 2H, CH_2N), 4.94 (s, 2H, *CH_2*NH), 1.42 (s, 9H, $(CH_3)_3$), 1.39 (s, 9H, $(CH_3)_3$); ^{13}C NMR ($CDCl_3$): δ = 163.2 (CO), 160.1 (C3), 159.8 (CO), 154.0 (C=N), 143.3 (C5), 136.6 (C6), 136.2 (C-Ph), 128.6 (CH-Ph), 128.5 (CH-Ph), 127.9 (CH-Ph), 126.1 (C4), 85.3 (*C*$(CH_3)_3$), 79.2 (*C*$(CH_3)_3$), 54.8 (CH_2N), 44.3 (CH_2NH), 28.2 ($(CH_3)_3$), 27.9 ($(CH_3)_3$); HRMS (ESI): *m/z* $[M+H]^+$ calcd for $C_{23}H_{32}N_5O_5$, 458.2403; found 458.2402.

2-Benzyl-4-[(*N,N'*-di(*tert*-butoxycarbonyl)guanidine]methylpyridazin-3(2*H*)-one (83). White solid; yield 83%; R_f = 0.2 (20% EtOAc/hexane); m.p. = 128.0 – 129.0 °C; ^{1}H NMR ($CDCl_3$): δ = 9.51 - 9.11 (m, 2H, 2xNH), 7.70 (d, 1H, *J* = 4.1 Hz, H6), 7.42 - 7.38 (m, 2H, H-Ph), 7.32 - 7.23 (m, 3H, H-Ph), 6.84 (d, 1H, *J* = 4.1 Hz, H5), 5.32 (s, 2H, CH_2N), 5.13 (s, 2H, *CH_2*NH), 1.50 (s, 6H, 2xCH_3), 1.42 (s, 6H, 2xCH_3), 1.27 (s, 6H, 2xCH_3); ^{13}C NMR ($CDCl_3$): δ = 163.5 (CO), 160.2 (CO), 159.6 (C3), 154.3 (C=N), 140.9 (C4), 136.2 (C-Ph), 135.9 (C6), 128.7 (CH-Ph), 128.5 (CH-Ph), 127.8 (CH-Ph), 124.8 (C5), 84.6 (*C*$(CH_3)_3$), 79.1 (*C*$(CH_3)_3$), 55.3 (CH_2N), 43.0 (CH_2NH), 28.2 ($(CH_3)_3$), 28.0 ($(CH_3)_3$), 27.7 ($(CH_3)_3$); HRMS (ESI): *m/z* $[M+Na]^+$ calcd for $C_{23}H_{31}N_5O_5Na$, 480.2223; found 480.2218.

2-[4-(Fluoromethyl)benzyl]-6-[(*N,N'*-di(*tert*-butoxycarbonyl)guanidine]methyl-5-methylpyridazin-3(2*H*)-one (79). Following a similar procedure as that used for the synthesis of **52-55**, compound **79** (15 mg, 60%) was obtained as a white solid from **74** (16 mg, 0.05 mmol) 1,3-bis(*tert*-butoxycarbonyl)guanidine (73 mg, 0.28 mmol) and K_2CO_3 (15 mg, 0.11 mmol) in DMF (2 mL) after purification by column chromatography on silica gel (30% EtOAc/hexane). R_f = 0.2 (50% EtOAc/hexane); m.p.= 64.1 – 65.0 °C; ^{1}H NMR ($CDCl_3$): δ = 9.56 – 9.21 (m, 2H, 2xNH), 7.36 (d, 2H, J = 7.9 Hz, H-Ph), 7.25 (d, 2H, J = 7.9 Hz, H-Ph), 6.65 (d, 1H, J = 1.0 Hz, H4), 5.28 (d, 2H, J_{HF} = 47.8 Hz, CH_2F), 5.18 (s, 2H, CH_2N), 5.02 (s, 2H, *CH_2*NH), 2.16 (d, 3H, J = 1.0 Hz, CH_3), 1.43 (s, 9H, $(CH_3)_3$), 1.25 (s, 9H, $(CH_3)_3$); ^{13}C NMR ($CDCl_3$): δ =163.5 (CO), 160.6 (CO), 160.1 (C3), 154.6 (C=N), 143.7, 141.6, 136.9 (C-Ph, $^5J_{CF}$ = 3.5 Hz), 135.9 (C-Ph, $^2J_{CF}$ = 17.4 Hz), 129.4 (CH-Ph), 128.4 (C4), 127.8 (CH-Ph, $^3J_{CF}$ = 5.9 Hz), 84.3 (CH_2F, $^1J_{CF}$ = 166.6 Hz), 84.1 (*C*$(CH_3)_3$), 79.0 (*C*$(CH_3)_3$), 54.2 (CH_2N), 44.2 (CH_2NH), 28.3 ($(CH_3)_3$), 27.8 ($(CH_3)_3$), 17.7 (CH_3); ^{19}F NMR ($CDCl_3$): δ = -207.1 (CH_2F); HRMS (ESI): *m/z* $[M+H]^+$ calcd for $C_{25}H_{35}FN_5O_5$, 504.26167; found 504.26110.

4-[4-(*N,N'*-di(*tert*-Butoxycarbonyl)guanidine)methyl]-2-(4-methoxymethyl)benzylpyridazin-3(2*H*)-one (84). Following a similar procedure as that used for the synthesis of **52-55**, compound **84** (15 mg, 62%) was obtained as a white solid from **75** (16 mg, 0.05 mmol), 1,3-bis(*tert*-butoxycarbonyl)guanidine (73 mg, 0.28 mmol) and K_2CO_3 (15 mg, 0.11 mmol) in DMF (2 mL) after purification by column chromatography on silica gel (20% EtOAc/hexane). R_f = 0.6 (50% EtOAc/hexane); m.p.= 134.3 – 135.0 °C; ^{1}H NMR ($CDCl_3$): δ =9.54 – 9.17 (m, 2H, 2xNH), 7.72 (d, 1H, J = 4.1 Hz, H6), 7.40 (d, 2H, J = 8.1 Hz, H-Ph), 7.27 (d, 2H, J = 8.1 Hz, H-Ph), 6.85 (d, 1H, J = 4.1 Hz, H5), 5.33 (s, 2H, CH_2N), 5.14 (s, 2H, *CH_2*NH), 4.42 (s, 2H, CH_2O), 3.35 (s, 3H, CH_3O), 1.43 (s, 9H, $(CH_3)_3$), 1.29 (s, 9H, $(CH_3)_3$); ^{13}C NMR ($CDCl_3$): δ = 163.7 (CO), 160.4 (CO), 159.7 (C3), 154.5 (C=N), 141.0 (C4), 138.1 (C-Ph), 136.1 (C6), 135.8 (C-Ph), 129.0 (CH-Ph), 128.0 (CH-Ph), 124.9 (C5), 84.8 (*C*$(CH_3)_3$), 79.3 (*C*$(CH_3)_3$), 74.4 (CH_2O), 58.2 (CH_3O), 55.1 (CH_2N), 43.2 (CH_2NH), 28.4 ($(CH_3)_3$), 27.9 ($(CH_3)_3$); HRMS (ESI): *m/z* $[M+H]^+$ calcd for $C_{25}H_{36}N_5O_6$, 502.26601; found 502.26617.

General procedure to synthesize guanidinium salts 1-4 and 5-14. To a solution of compound **52-55** or **76-84** (0.13 mmol) in 1,4-dioxane, a solution HCl 4M in 1,4-dioxane

(0.78 mmol HCl per Boc group) was added to reach a final concentration of 0.2 M. The reaction mixture was stirred at 55 °C for 5 h. After the solvent was removed, the residue was purified by reverse phase chromatography (H_2O) to afford the corresponding hydrochloride.

Dihydrochloride salt of 2-(4-guanidinomethyl)benzyl-6-guanidinomethylpyridazin-3(2*H*)-one (1). White solid; yield 80%; m.p. = 159.6 – 159.9 °C; ^{1}H NMR (CD_3OD): δ = 7.46 (d, 1H, J = 9.5 Hz, H5), 7.44 (d, 2H, J = 8.2 Hz, H-Ph), 7.34 (d, 2H, J = 8.2 Hz, H-Ph), 7.05 (d, 1H, J = 9.5 Hz, H4), 5.35 (s, 2H, CH_2N), 4.45 (s, 2H, NHCH_2-pyridazinone), 4.42 (s, 2H, CH_2NH); ^{13}C NMR (CD_3OD): δ = 161.7 (C3), 159.3 (C=N), 158.7 (C=N), 145.0 (C6), 137.5 (C-Ph), 137.3 (C-Ph), 133.3 (C5), 131.4 (C4), 130.0 (CH-Ph), 128.6 (CH-Ph), 56.2 (CH_2N), 45.6 (CH_2NH), 44.4 ($NHCH_2$-pyridazinone); HRMS (ESI): *m/z* $[M+H]^+$ calcd for $C_{15}H_{21}N_8O$, 329.18328; found 329.18293; HPLC: 94.2% (*t*R: 2.78 min).

Dihydrochloride salt of 2-(4-guanidinomethyl)benzyl-6-guanidinomethyl-5-methylpyridazin-3(2*H*)-one (2). White solid; yield 83%; m.p. = 163.0 – 163.5 °C; ^{1}H NMR (CD_3OD): δ = 7.44 (d, 2H, J = 8.1 Hz, H-Ph), 7.34 (d, 2H, J = 8.1 Hz, H-Ph), 6.84 (d, 1H, J = 1.0 Hz, H4), 5.33 (s, 2H, CH_2N), 4.47 (s, 2H, NHCH_2-pyridazinone), 4.42 (s, 2H, CH_2NH), 2.27 (d, 3H, J = 1.0 Hz, CH_3); ^{13}C NMR (CD_3OD): δ = 162.1 (C3), 159.3 (C=N), 158.7 (C=N), 145.0, 144.4, 137.4 (2xC-Ph), 130.0 (CH-Ph), 129.3 (C4), 128.6 (CH-Ph), 55.7 (CH_2N), 45.6 (CH_2NH), 43.1 ($NHCH_2$-pyridazinone), 17.3 (CH_3); HRMS (ESI): *m/z* $[M+H]^+$ calcd for $C_{16}H_{23}N_8O$, 343.19893; found 343.19848; HPLC: 99.0% (*t*R: 2.95 min).

Dihydrochloride salt of 2-(4-guanidinomethyl)benzyl-5-guanidinomethylpyridazin-3(2*H*)-one (3). White solid; yield 85%; m.p. = 151.0 – 151.7 °C; ^{1}H NMR (CD_3OD): δ = 7.94 (d, 1H, J = 2.1 Hz, H6), 7.43 (d, 2H, J = 8.2 Hz, H-Ph), 7.33 (d, 2H, J = 8.2 Hz, H-Ph), 6.84 (dt, 1H, J = 2.1, 1.1 Hz, H4), 5.36 (s, 2H, CH_2N), 4.44 (d, 2H, J = 1.1 Hz, NHCH_2-pyridazinone), 4.42 (s, 2H, CH_2NH); ^{13}C NMR (CD_3OD): δ = 162.0 (C3), 159.0 (C=N), 158.7 (C=N), 144.8 (C5), 138.2 (C6), 137.5 (C-Ph), 137.4 (C-Ph), 130.0 (CH-Ph), 128.6 (CH-Ph), 125.9 (C4), 55.9 (CH_2N), 45.6 (CH_2NH), 42.3 ($NHCH_2$-

pyridazinone); HRMS (ESI): *m/z* $[M+H]^+$ calcd for $C_{15}H_{21}N_8O$, 329.18296; found 329.18328; HPLC: 99.5% (*t*R: 3.02 min).

Dihydrochloride salt of 2-(4-guanidinomethyl)benzyl-4-guanidinomethylpyridazin-3(2*H*)-one (4). White solid; yield 94%; m.p. = 160.0 – 160.7 °C; ^{1}H NMR (CD_3OD): δ = 7.99 (d, 1H, J = 4.1 Hz, H6), 7.44 (d, 2H, J = 8.2 Hz, H-Ph), 7.36 (dt, 1H, J = 4.1, 1.1 Hz, H5), 7.33 (d, 2H, J = 8.2 Hz, H-Ph), 5.39 (s, 2H, CH_2N), 4.41 (s, 2H, CH_2NH), 4.35 (d, 2H, J = 1.1 Hz, NHCH_2-pyridazinone); ^{13}C NMR (CD_3OD): δ = 161.7 (C3), 159.3 (C=N), 158.7 (C=N), 139.4 (C4), 138.5 (C6), 137.5 (C-Ph), 137.4 (C-Ph), 130.1 (CH-Ph), 129.3 (C5), 128.6 (CH-Ph), 56.1 (CH_2N), 45.6 (CH_2NH), 41.5($NHCH_2$-pyridazinone); HRMS (ESI): *m/z* $[M+H]^+$ calcd for $C_{15}H_{21}N_8O$, 329.18328; found 329.18292; HPLC: 99.6% (*t*R: 3.40 min).

Hydrochloride salt of 6-guanidinomethyl-2-methylpyridazin-3(2*H*)-one (5). White solid; yield 77%; m.p. = 150.0 – 150.5 °C; ^{1}H NMR (D_2O): δ = 7.56 (d, 1H, J = 9.5 Hz, H5), 7.12 (d, 1H, J = 9.5 Hz, H4), 4.48 (s, 2H, CH_2), 3.81 (s, 3H, CH_3); ^{13}C NMR (D_2O): δ = 162.1 (C3), 157.2 (C=N), 144.7 (C6), 132.6 (C5), 129.6 (C4), 43.1 (CH_2), 40.3 (CH_3); HRMS (ESI): *m/z* $[M+H]^+$ calcd for $C_7H_{12}N_5O$, 182.1042; found 182.1038; HPLC: 99.2% (*t*R: 3.2 min).

Hydrochloride salt of 6-guanidinomethyl-2,5-dimethylpyridazin-3(2*H*)-one (6). White solid; yield 92%; m.p. = 160.0 – 162.0 °C; ^{1}H NMR (D_2O): δ = 6.94 (s, 1H, H4), 4.48 (s, 2H, CH_2), 3.78 (s, 3H, CH_3N), 2.27 (s, 3H, CH_3); ^{13}C NMR (D_2O): δ = 162.4 (C3), 157.3 (C=N), 144.6, 144.1, 127.5 (C4), 41.9 (CH_2), 39.9 (CH_3N), 16.5 (CH_3); HRMS (ESI): *m/z* $[M+H]^+$ calcd for $C_8H_{14}N_5O$, 196.1198; found 196.1205; HPLC: 95% (*t*R: 8.4 min).

Hydrochloride salt of 2-benzyl-6-guanidinomethyl-5-methylpyridazin-3(2*H*)-one (7). White solid; yield 86%; m.p. = 140.0 – 140.4 °C; ^{1}H NMR (D_2O): δ = 7.44 - 7.36 (m, 3H, H-Ph), 7.35 - 7.30 (m, 2H, H-Ph), 6.93 (s, 1H, H4), 5.33 (s, 2H, CH_2N), 4.44 (s, 2H, CH_2NH), 2.25 (s, 3H, CH_3); ^{13}C NMR (D_2O): δ = 161.9 (C3), 157.4 (C=N), 144.5, 144.4, 135.7 (C-Ph), 128.7 (CH-Ph), 128.0, 127.9, 127.8 (CH-Ph), 54.7 (CH_2N), 41.7 (CH_2NH),

16.4 (CH_3); HRMS (ESI): *m/z* $[M+H]^+$ calcd for $C_{14}H_{18}N_5O$, 272.1511; found 272.1514; HPLC: 95.6% (*t*R: 22.7 min).

Hydrochloride salt of 2-(4-fluoromethyl)benzyl-6-guanidinomethyl-5-methylpyridazin-3(2*H*)-one (8). White solid; yield 33%; m.p. = 131.7 – 132.0 °C; ^1H NMR (CD_3OD): δ = 7.46 – 7.36 (m, 4H, H-Ph), 6.86 (d, 1H, J = 1.2 Hz, H4), 5.36 (s, 2H, J_{HF} = 47.9 Hz, CH_2F), 5.35 (s, 2H, CH_2N), 4.46 (s, 2H, *CH_2*NH), 2.27 (d, 3H, J = 1.2 Hz, CH_3); ^{13}C NMR (CD_3OD): δ = 162.1 (C3), 159.4 (C=N), 144.9, 144.4, 138.1 (C-Ph, $^5J_{CF}$ = 2.8 Hz), 137.8 (C-Ph, $^2J_{CF}$ = 17.2 Hz), 129.7 (CH-Ph, $^4J_{CF}$ = 1.4 Hz), 129.3 (C4), 128.9 (CH-Ph, $^3J_{CF}$ = 5.6 Hz), 85.1 (CH_2F, $^1J_{CF}$ = 164.9 Hz), 55.7 (CH_2N), 43.0 (CH_2NH), 17.3 (CH_3); ^{19}F NMR (CD_3OD): δ = -208.7 (CH_2F); HRMS (ESI): *m/z* $[M+H]^+$ calcd for $C_{15}H_{19}FN_5O$, 304.15681; found 304.15664; HPLC: 94.0% (*t*R: 3.90 min).

Hydrochloride salt of 2-(4-chloromethyl)benzyl-6-guanidinomethyl-5-methylpyridazin-3(2*H*)-one (9). White solid; yield 46%; m.p. = 128.9 – 129.3 °C; ^1H NMR (CD_3OD): δ = 7.40 (s, 4H, H-Ph), 6.86 (d, 1H, J = 1.2 Hz, H4), 5.33 (s, 2H, CH_2N), 4.64 (s, 2H, CH_2Cl), 4.46 (s, 2H, *CH_2*NH), 2.27 (d, 3H, J = 1.2 Hz, CH_3); ^{13}C NMR (CD_3OD): δ = 160.7 (C3), 158.0 (C=N), 143.5, 143.0, 137.8 (C-Ph), 136.4 (C-Ph), 128.6 (CH-Ph), 128.4 (CH-Ph), 127.9 (C4), 54.2 (CH_2N), 45.1 (CH_2Cl), 41.6 (CH_2NH), 15.9 (CH_3); HRMS (ESI): *m/z* $[M+H]^+$ calcd for $C_{15}H_{19}ClN_5O$, 320.12726; found 320.12648; HPLC: 98.2% (*t*R: 6.25 min).

Hydrochloride salt of 5-guanidinomethyl-2-methylpyridazin-3(2*H*)-one (10). White solid; yield 92%; m.p. = 160.0 – 160.5 °C; ^1H NMR (D_2O): δ = 8.01 (d, 1H, J = 2.1 Hz, H6), 6.98 (d, 1H, J = 2.1 Hz, H4), 4.49 (s, 2H, CH_2), 3.80 (s, 3H, CH_3); ^{13}C NMR (D_2O): δ = 162.2 (C3), 157.1 (C=N), 143.6 (C5), 137.7 (C6), 124.6 (C4), 41.0 (CH_2), 39.9 (CH_3); HRMS (ESI): *m/z* $[M+H]^+$ calcd for $C_7H_{12}N_5O$, 182.1042; found 182.1047; HPLC: 96.1% (*t*R: 3.4 min).

Hydrochloride salt of 4-guanidinomethyl-2-methylpyridazin-3(2*H*)-one (11). White solid; yield 89%; m.p. = 170.0 – 170.6 °C; ^1H NMR (D_2O): δ = 8.04 (d, 1H, J = 4.3 Hz, H6), 7.47 (dt, 1H, J = 4.3, 1.2 Hz, H5), 4.39 (d, 2H, J = 1.2 Hz, CH_2), 3.83 (s, 3H, CH_3); ^{13}C NMR (D_2O): δ = 161.4 (C3), 157.2 (C=N), 138.3 (C6), 137.6 (C4), 128.6 (C5), 40.0

(CH_3), 39.9 (CH_2); HRMS (ESI): *m/z* $[M+H]^+$ calcd for $C_7H_{12}N_5O$, 182.1042; found 182.1038; HPLC: 97.2% (*t*R: 4.7 min).

Hydrochloride salt of 2-benzyl-5-guanidinomethylpyridazin-3(2*H*)-one (12). White solid; yield 92%; m.p. = 139.0 – 140.0 °C; ^1H NMR (D_2O): δ = 8.03 (d, 1H, *J* = 2.1 Hz, H6), 7.45 – 7.36 (m, 3H, H-Ph), 7.35 – 7.30 (m, 2H, H-Ph), 7.02 – 6.98 (m, 1H, H4), 5.37 (s, 2H, CH_2N), 4.48 (d, 2H, *J* = 1.2 Hz, CH_2NH); ^{13}C NMR (D_2O): δ = 161.7 (C3), 157.1 (C=N), 143.7 (C5), 138.2 (C6), 135.4 (C-Ph), 128.8 (CH-Ph), 128.1 (CH-Ph), 127.6 (CH-Ph), 125.3 (C4), 55.1 (CH_2N), 41.0 (CH_2NH); HRMS (ESI): *m/z* $[M+H]^+$ calcd for for $C_{13}H_{16}N_5O$, 258.1355; found 258.1360; HPLC: 99% (*t*R: 22.4 min).

Hydrochloride salt of 2-benzyl-4-guanidinomethylpyridazin-3(2*H*)-one (13). White solid; yield 90%; m.p. = 136.0 – 137.1 °C; ^1H NMR (D_2O): δ = 8.07 (d, 1H, *J* = 4.2 Hz, H6), 7.49 - 7.46 (m, 1H, H5), 7.45 - 7.33 (m, 5H, H-Ph), 5.41 (s, 2H, CH_2N), 4.36 (d, 2H, *J* = 1.2 Hz, CH_2NH); ^{13}C NMR (D_2O): δ = 161.0 (C3), 157.2 (C=N), 138.7 (C6), 138.4 (C4), 135.5 (C-Ph), 128.8 (CH-Ph), 128.5 (C5), 128.1 (CH-Ph), 127.6 (CH-Ph), 55.4 (CH_2N), 40.0 (CH_2NH); HRMS (ESI): *m/z* $[M+H]^+$ calcd for $C_{13}H_{16}N_5O$, 258.1355; found 258.1366; HPLC: 95.3% (*t*R: 24.1 min).

Hydrochloride salt of 4-guanidinomethyl-2-(4-methoxymethyl)benzylpyridazin-3(2*H*)-one (14). White solid; yield 99%; m.p. = 143.2 – 143.8 °C; ^1H NMR (CD_3OD): δ = 7.98 (d, 1H, *J* = 4.1 Hz, H6), 7.40 (d, 2H, *J* = 8.2 Hz, H-Ph), 7.34 (dt, 1H, *J* = 4.1, 1.2 Hz, H5), 7.31 (d, 2H, *J* = 8.2 Hz, H-Ph), 5.38 (s, 2H, CH_2N), 4.44 (s, 2H, CH_2O), 4.34 (d, 2H, *J* = 1.2 Hz, CH_2NH), 3.37 (s, 3H, CH_3); ^{13}C NMR (CD_3OD): δ = 161.7 (C3), 159.3 (C=N), 139.4 (C4), 139.4 (C-Ph), 138.4 (C6), 136.9 (C-Ph), 129.6 (CH-Ph), 129.3(C5), 129.1 (CH-Ph), 75.2 (CH_2O), 58.3 (CH_3), 56.3 (CH_2N), 41.5 (CH_2NH); HRMS (ESI): *m/z* $[M+H]^+$ calcd for $C_{15}H_{20}N_5O_2$, 302.16115; found 302.16018; HPLC: 98.4% (*t*R: 5.28 min).

Biophysical Studies

DNA thermal denaturation assays.

Thermal melting experiments were conducted with a Varian Cary 300 Bio spectrophotometer equipped with a 6 × 6 multicell temperature-controlled block. Temperature was monitored with a thermistor inserted into a 1 mL quartz cuvette containing the same volume of buffer as in the sample cells. Absorbance changes at 295 nm for st-DNA were monitored from a range of 30 °C to 90 °C with a heating rate of 1 °C/minute and a data collection rate of five points per °C. The stock solution of st-DNA was prepared in phosphate buffer solutions contained 10 mM Na_2HPO_4/NaH_2PO_4 adjusted to pH 7. The stock solution of ligands was prepared in EtOH (1 mM). A quartz cell with a 1 cm path length was filled with a 1 mL solution of DNA (150 µM base) and compound solutions (15 µM) in phosphate buffer, adjusted to pH 7 so that a ligand to DNA base ratio of 0.1 was obtained.

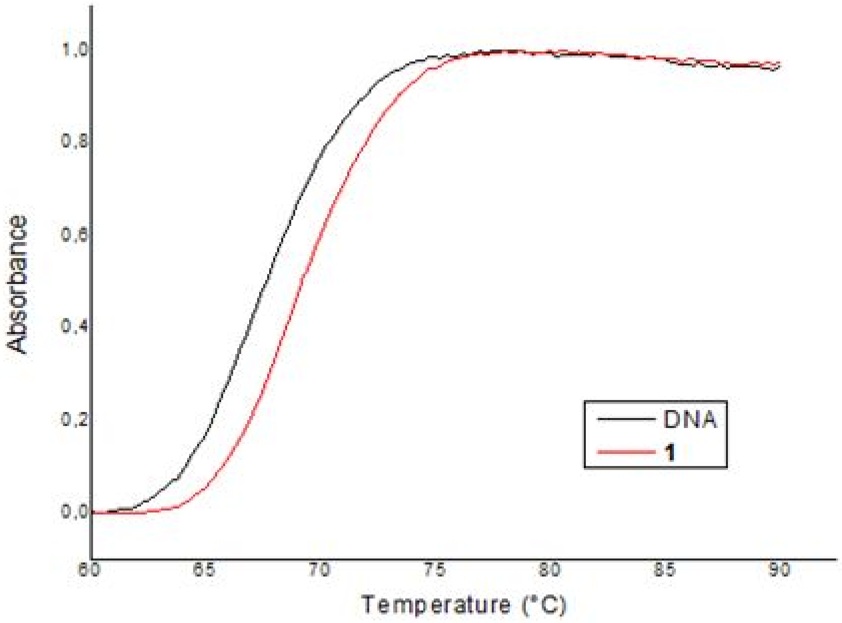

Figure S15. Graph showing the DNA thermal denaturation results of st-DNA alone and after adding compound **1**. X-axis represents temperature (ºC) and Y axis represents absorbance (cm^{-1}).

Cytotoxicity assays

Cytotoxicity studies on NCI-H460, A2780 and MCF-7 cells were performed by using a colorimetric MTT (3-(4,5-dimethyl-2-thiazolyl)-2,5-diphenyl-2H-tetrazolium bromide) assay [8,9,10]. In brief, cells were seeded in 96-well plates (15000 cells per well for NCI-H460 cell line, 4000 for A2780 cell line and 10000 for MCF-7 cell line), incubated for 24 h in the culture medium and treated at 37 ^{o}C for 48 h (NCI-H460) and 96 h (A2780

and MCF-7) with varying doses of evaluated compounds and the reference drug, cisplatin, dissolved in DMSO. Three wells were used for each of the variants tested. Aliquots of MTT solution in phosphate buffered saline (10 μL) were added to each well and incubated for 4 h. The colour formed was quantified by a spectrophotometric plate reader (Tecan Ultra evolution) at 595 nm wavelength. In all experiments, DMSO controls were included. The percentage of inhibition of cell viability was calculated by the formula % inhibiton=100-((AO*100)/AT) where AO is the absorbance observed in the treated wells and AT is the absorbance observed in the DMSO control wells.

The cytotoxic potency of compounds, measured as 50% inhibitory concentrations (IC_{50}), was calculated from concentration-effect curves by using GraphPad Prism software, version 2.01. Correlation coefficients (r^2) were higher than 0.995 for the compounds tested.

^{1}H and ^{13}C NMR spectra of pyridazin-3(2H)-one-based guanidine derivatives 1-14 and ^{19}F NMR spectrum of compound 8

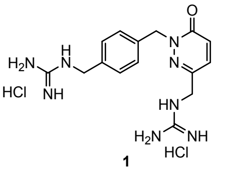

1

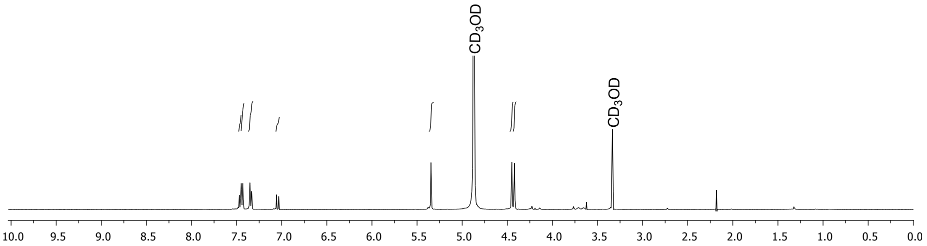

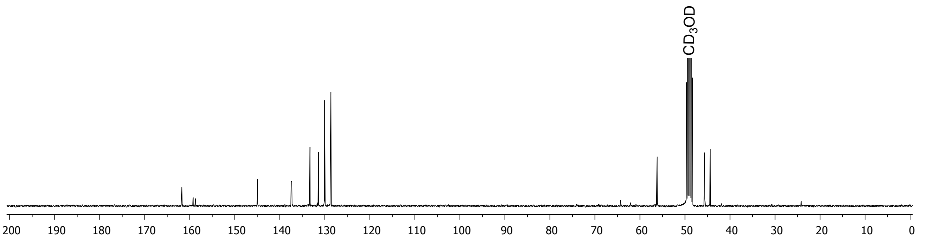

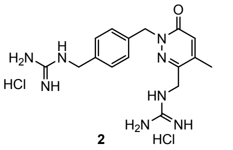

2

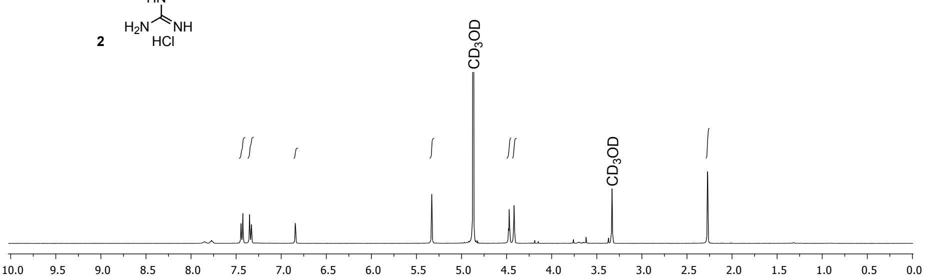

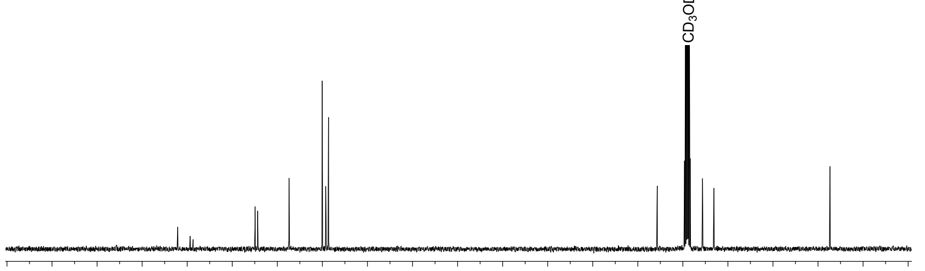

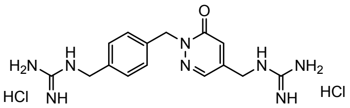

O
H2N
H
N
N
N
N
H
N
NH2
HCl
NH
NH
HCl

3

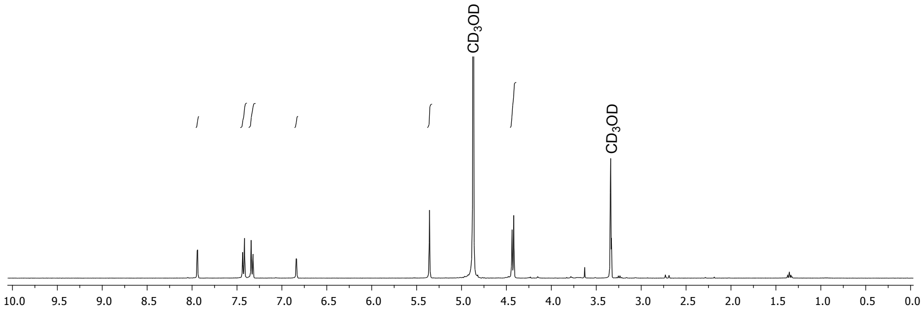

CD3OD
CD3OD
10.0 9.5 9.0 8.5 8.0 7.5 7.0 6.5 6.0 5.5 5.0 4.5 4.0 3.5 3.0 2.5 2.0 1.5 1.0 0.5 0.0

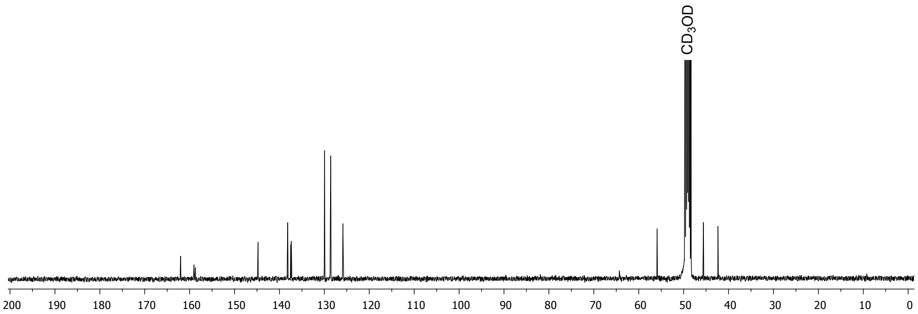

CD3OD
200 190 180 170 160 150 140 130 120 110 100 90 80 70 60 50 40 30 20 10 0

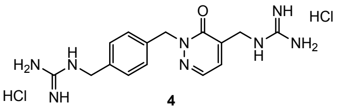

O
NH
HCl
H2N
H
N
N
N
N
H
NH2
HCl
NH

4

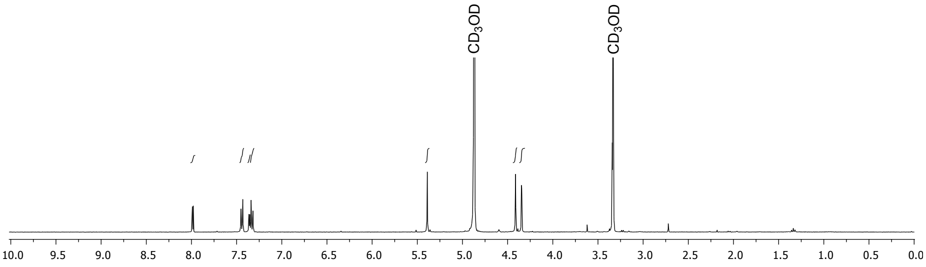

CD3OD
CD3OD
10.0 9.5 9.0 8.5 8.0 7.5 7.0 6.5 6.0 5.5 5.0 4.5 4.0 3.5 3.0 2.5 2.0 1.5 1.0 0.5 0.0

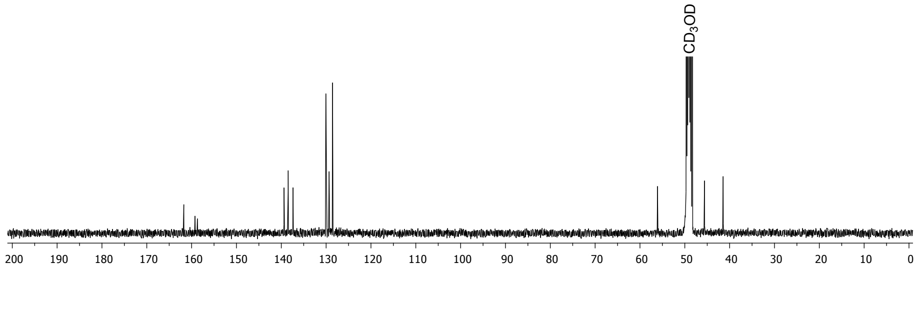

CD3OD
200 190 180 170 160 150 140 130 120 110 100 90 80 70 60 50 40 30 20 10 0

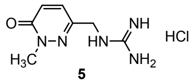

5

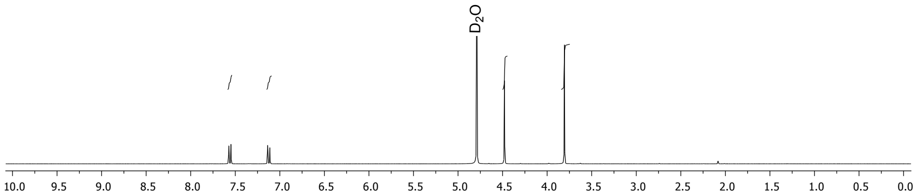

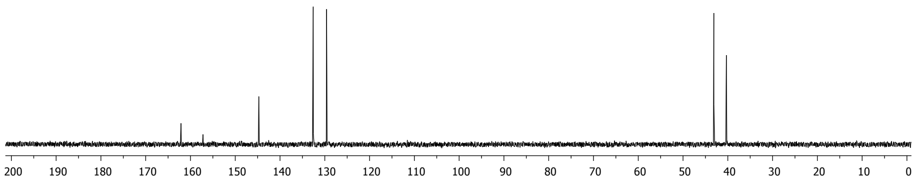

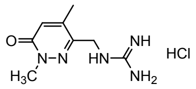

6

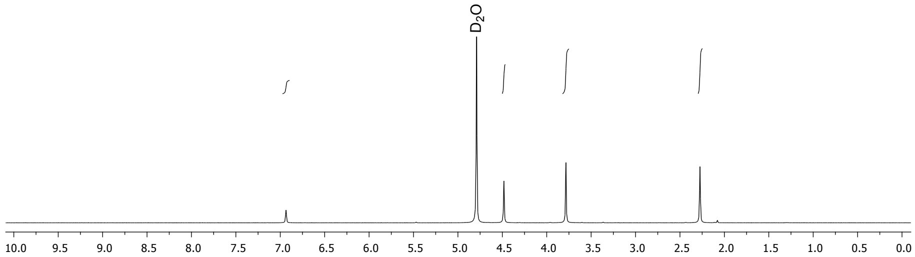

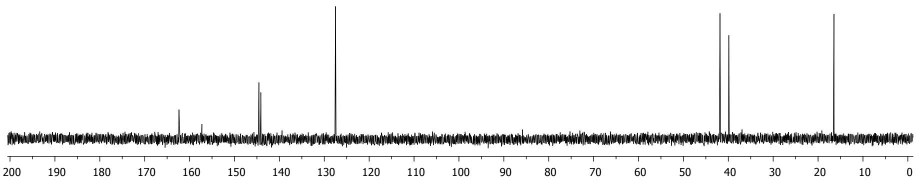

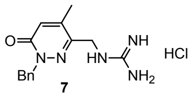

7

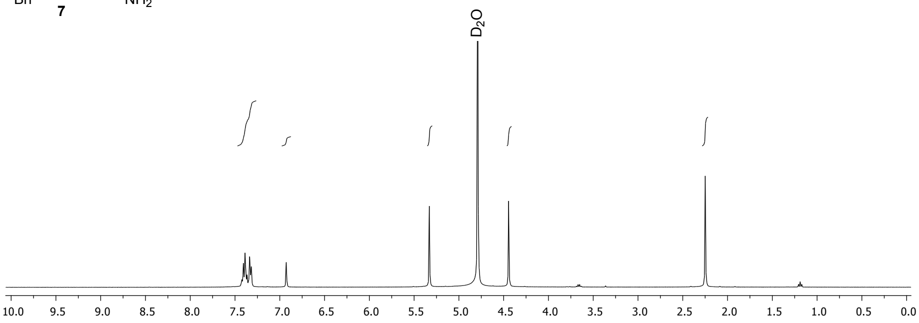

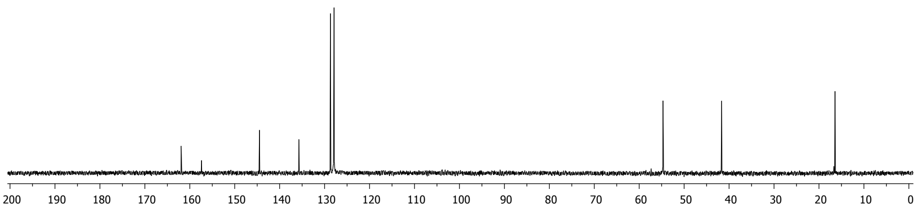

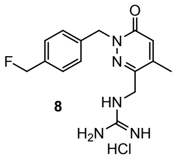

8

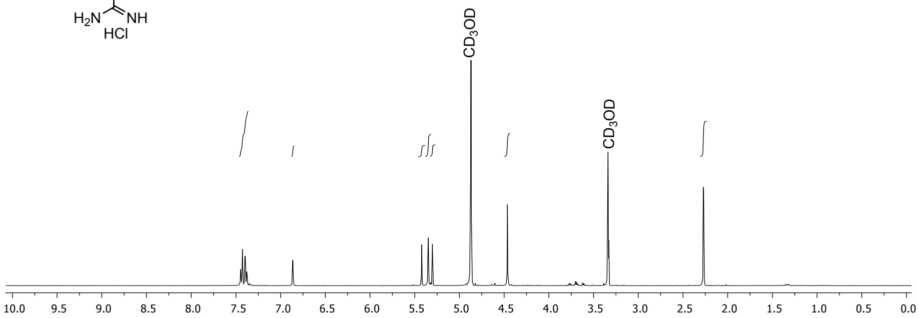

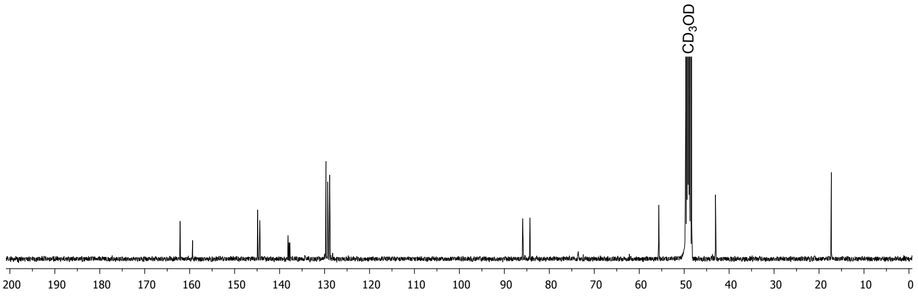

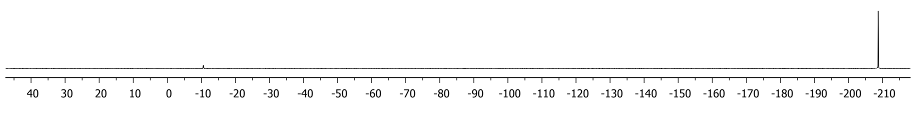

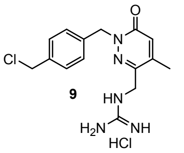

9

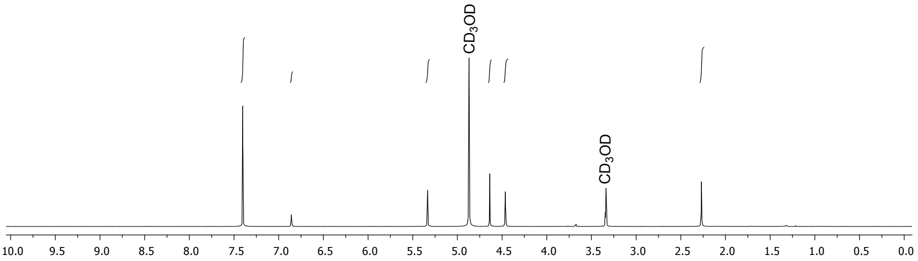

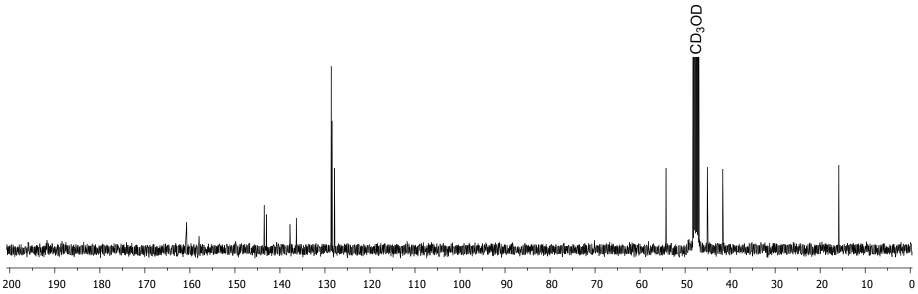

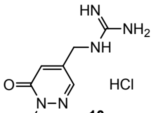

10

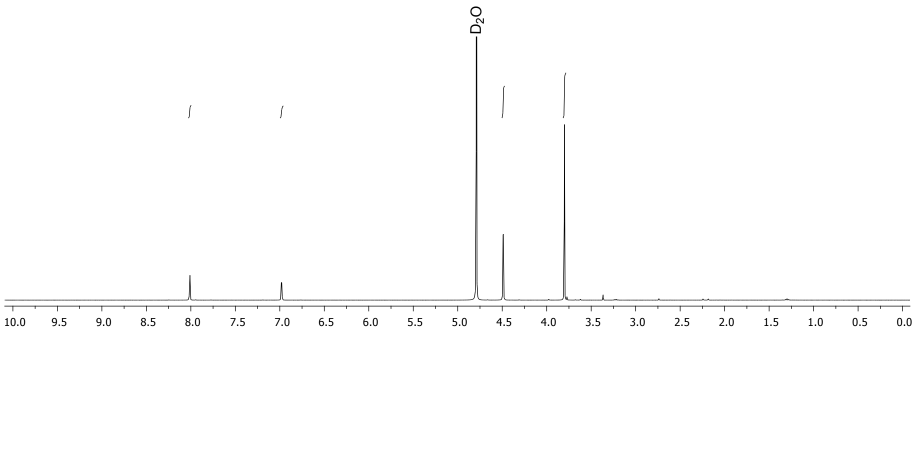

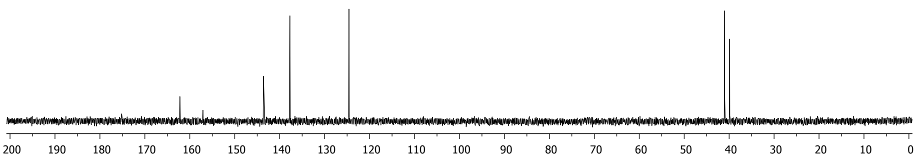

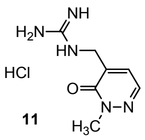

11

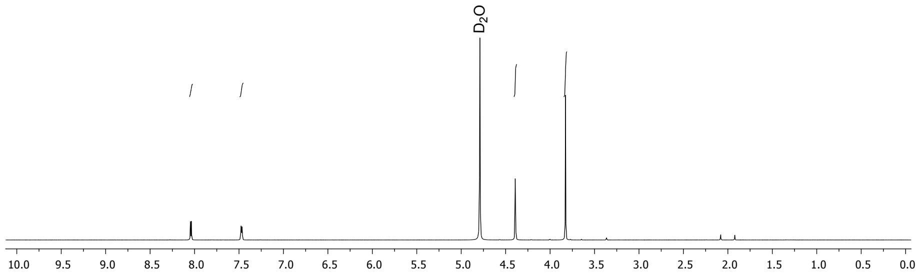

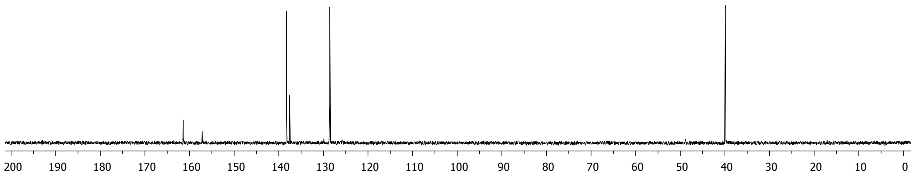

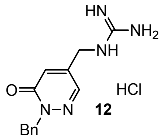

12

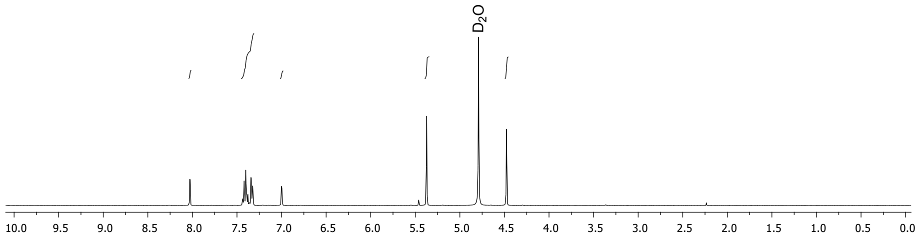

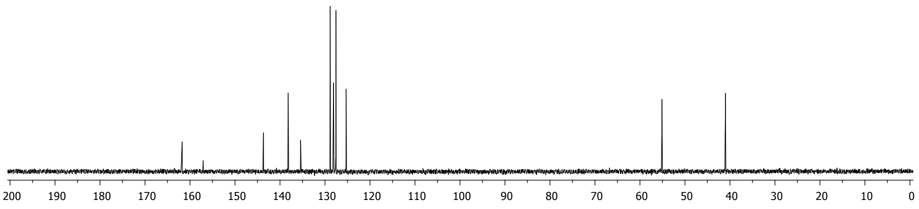

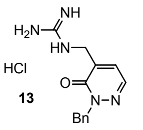
NH
H2N
HN
HCl
O
N
N
Bn

13

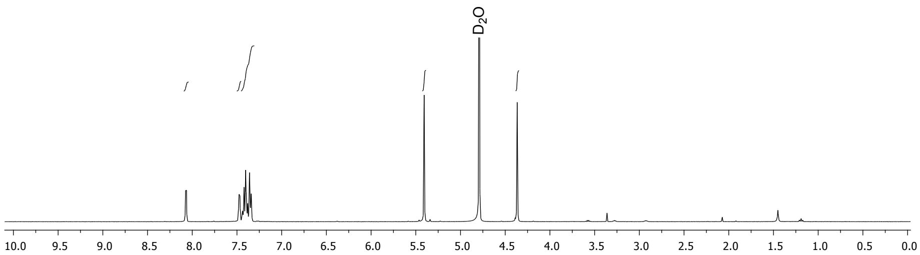
D2O
10.0 9.5 9.0 8.5 8.0 7.5 7.0 6.5 6.0 5.5 5.0 4.5 4.0 3.5 3.0 2.5 2.0 1.5 1.0 0.5 0.0

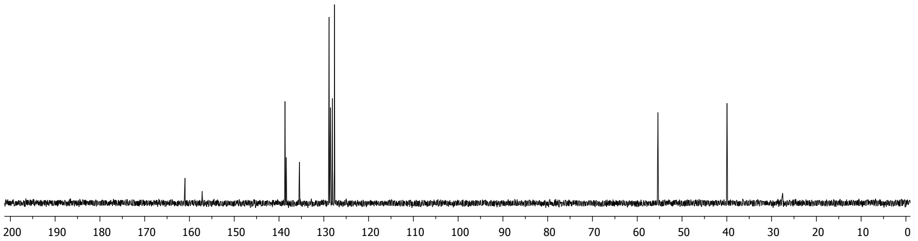
200 190 180 170 160 150 140 130 120 110 100 90 80 70 60 50 40 30 20 10 0

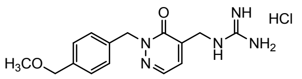
O
N
N
NH
H
NH2
HCl
H3CO

14

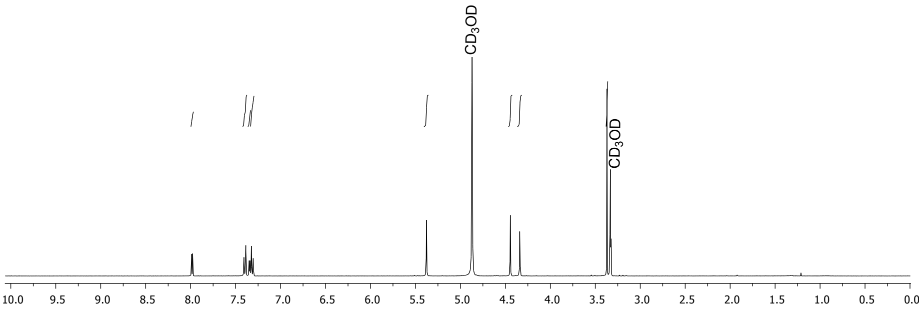
CD3OD
CD3OD
10.0 9.5 9.0 8.5 8.0 7.5 7.0 6.5 6.0 5.5 5.0 4.5 4.0 3.5 3.0 2.5 2.0 1.5 1.0 0.5 0.0

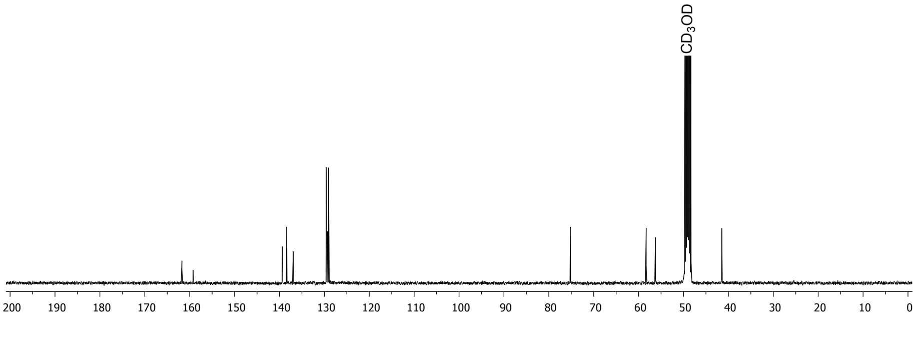
CD3OD
200 190 180 170 160 150 140 130 120 110 100 90 80 70 60 50 40 30 20 10 0

References.

1. Gaussian 16, Revision C.01, Frisch, M. J.; Trucks, G. W.; Schlegel, H. B.; Scuseria, G. E.; Robb, M. A.; Cheeseman, J. R.; Scalmani, G.; Barone, V.; Petersson, G. A.; Nakatsuji, H.; Li, X.; Caricato, M.; Marenich, A. V.; Bloino, J.; Janesko, B. G.; Gomperts, R.; Mennucci, B.; Hratchian, H. P.; Ortiz, J. V.; Izmaylov, A. F.; Sonnenberg, J. L.; Williams-Young, D.; Ding, F.; Lipparini, F.; Egidi, F.; Goings, J.; Peng, B.; Petrone, A.; Henderson, T.; Ranasinghe, D.; Zakrzewski, V. G.; Gao, J.; Rega, N.; Zheng, G.; Liang, W.; Hada, M.; Ehara, M.; Toyota, K.; Fukuda, R.; Hasegawa, J.; Ishida, M.; Nakajima, T.; Honda, Y.; Kitao, O.; Nakai, H.; Vreven, T.; Throssell, K.; Montgomery, J. A., Jr.; Peralta, J. E.; Ogliaro, F.; Bearpark, M. J.; Heyd, J. J.; Brothers, E. N.; Kudin, K. N.; Staroverov, V. N.; Keith, T. A.; Kobayashi, R.; Normand, J.; Raghavachari, K.; Rendell, A. P.; Burant, J. C.; Iyengar, S. S.; Tomasi, J.; Cossi, M.; Millam, J. M.; Klene, M.; Adamo, C.; Cammi, R.; Ochterski, J. W.; Martin, R. L.; Morokuma, K.; Farkas, O.; Foresman, J. B.; Fox, D. J. Gaussian, Inc., Wallingford CT, 2016.

2. Trott, O.; Olson, A. J. AutoDock Vina: improving the speed and accuracy of docking with a new scoring function, efficient optimization and multithreading, *J. Comput. Chem*. **2010**, *31*, 455-461. DOI: 10.1002/jcc.21334.

3. Edwards, K.J., Jenkins, T.C., Neidle, S. Crystal structure of a pentamidine-oligonucleotide complex: implications for DNA-binding properties. Biochemistry 1992, 31, 7104-7109.

4. Humphrey, W.; Dalke, A.; Schulten, K. VMD - Visual Molecular Dynamics, *J. Molec. Graphics*, **1996**, *14*, 33-38. DOI: 10.1016/0263-7855(96)00018-5.

5. Costas, T.; Besada, P.; Piras, A.; Acevedo, L.; Yañez, M.; Orallo, F.; Lagua, R.; Terán, C. Bioorg. Med. Chem. Lett 2010, 20, 6624-6627.

6. Costas, T.; Costas-Lago, M. C.; Vila, N.; Besada, P.; Cano, E.; Terán, C. Eur. J. Med. Chem. 2015, 94, 113-122.

7. Besada, P.; Viña, D.; Costas, T.; Costas-Lago, M. C.; Vila, N.; Torres-Terán, I.; Sturlese, M.; Moro, S.; Terán, C. Bioorg. Chem. 2021, 105203.

8. Descôteaux, C.; Provencher-Mandeville, J.; Mathieu, I.; Perron, V.; Mandal, S.K.; Asselin, E.; Berube, G. Bioorg. Med. Chem. Lett. 2003, 13, 3927-3931.

9. Reithofer, M.R.; Valiahdi, S.M.; Jakupec, M.A.; Arion, V.B.; Egger, A. Galanski, M.; Keppler, B.K. J. Med. Chem. 2007, 50, 6692-6699.

10. Martinez, A.; Lorenzo, J.; Prieto, M.J.; Llorens, R.; Font-Bardia, M.; Solans, X.; Avilés, F.X.; Moreno, V. ChemBioChem 2005, 6, 2068-2077.
